# Data-Driven Discovery of Composition–Structure–Property Relationship in Novel Wave-Transparent High-Entropy Rare Earth Disilicate

**DOI:** 10.34133/research.1308

**Published:** 2026-06-01

**Authors:** Shuping Wen, Zhilin Tian, Yuhong Du, Lin Chi, Zhilin Chen, Liya Zheng, Bin Li

**Affiliations:** School of Materials, Shenzhen Campus of Sun Yat-sen University, Shenzhen 518107, China.

## Abstract

Developing advanced materials with simultaneously excellent wave transparency and efficient thermal insulation is critical for hypersonic vehicles. While rare earth disilicates (RE_2_Si_2_O_7_) are promising candidates, their vast chemical space and complex polymorphism hinder precise property modulation. Herein, we establish an integrated high-throughput experimental and machine learning strategy to systematically investigate the composition–structure–property relationship of high-entropy RE_2_Si_2_O_7_. The results demonstrate that the average RE^3+^ ionic radius determines the phase boundary. Notably, Sc incorporation jointly reduces both the dielectric constant and thermal conductivity. Specifically, the small size and strong electron localization of Sc minimize the polarizability, while its severe size and mass mismatch with other RE elements intensify phonon scattering. The model’s generalization is further validated by designing a series of high-entropy RE_2_Si_2_O_7_ containing 5 to 9 distinct RE elements. Ultimately, the (Ho_1/5_Tm_1/5_Yb_1/5_Lu_1/5_Sc_1/5_)_2_Si_2_O_7_ high-entropy ceramic achieves a low dielectric constant (*ε* = 5.4) and a low thermal conductivity (*κ* = 1.3 W·m^−1^·K^−1^). This data-driven strategy provides a new pathway for the rational design of advanced high-entropy wave-transparent materials for extreme environments.

## Introduction

Antenna windows are key components in aerospace vehicles, protecting antenna systems from environmental factors while ensuring the efficient transmission of electromagnetic waves [1,2]. With the rapid development of hypersonic vehicles, antenna windows face severe challenges caused by aerodynamic heating. Extremely harsh environments place greater demands on antenna window materials, known as wave-transparent materials. Low dielectric constant and low dielectric loss tangent are necessary to maintain the stable transmission of electromagnetic waves [3,4]. Additionally, wave-transparent materials should possess excellent thermal insulation properties to effectively block the external heat flux [5,6]. However, traditional candidates (e.g., SiO_2_, Al_2_O_3_, Si_3_N_4_, and BN) suffer from inherent performance trade-offs, struggling to simultaneously meet the stringent thermal, mechanical, and dielectric demands in high-temperature environments [7–14]. The scarcity of high-temperature wave-transparent materials severely restricts the development of hypersonic aircraft. Therefore, it is urgent to expand the existing system of high-temperature wave-transparent materials and elucidate the key factors governing the dielectric and thermal properties of these materials, which is of great importance for the development of high-performance antenna windows.

Rare earth (RE) elements play an important role in high-temperature structural materials [15,16]. Introducing RE to improve the thermal resistance offers new perspectives for the design of high-temperature wave-transparent materials. Recent studies have shown that some RE silicates exhibit low and stable dielectric constants at elevated temperatures, primarily due to the predominance of strong Si–O covalent bonds in the silicate framework, with the presence of RE cations further restricting ionic and electronic polarization [17,18]. Additionally, some RE disilicates (RE_2_Si_2_O_7_) feature weakly bonded atomic planes that facilitate deformation mechanisms (e.g., deformation twinning, dislocation glide, and climb), which imparts them with good damage tolerance, further enhancing their potential for use in harsh environments [19,20].

Generally, high-entropy ceramics are defined as solid solutions of 5 or more cations or anions (5 to 35 at. %) with a high configurational entropy (Δ*S*_mix_ ≥ 1.5R), typically forming 1 or 2 primary phases [21,22]. Notably, high-entropy ceramics have been demonstrated to exhibit superior overall properties exceeding those of their individual counterparts, originating from the 4 core effects of high-entropy materials: high entropy, lattice distortion, sluggish diffusion, and the cocktail effect. The formation of multicomponent solid solutions endows RE_2_Si_2_O_7_ with diverse crystal structures and tailorable properties [23–25]. Thus, applying the high-entropy strategy to RE_2_Si_2_O_7_ ceramic design provides new horizons for developing high-performance wave-transparent materials [26–28]. However, the underlying mechanisms for the phase formation and property modulation of high-entropy RE_2_Si_2_O_7_ remain unclear. Furthermore, the design of high-entropy RE_2_Si_2_O_7_ ceramics involves a vast compositional space, posing considerable challenges to traditional trial-and-error research methodologies.

High-throughput experimentation offers marked advantages in research by enabling the rapid execution of numerous experiments and generating large amounts of data, leading to faster discovery and optimization of materials. Meanwhile, machine learning can analyze vast amounts of data to identify the key parameters that determine the material’s performance [29–31]. It can uncover hidden correlations and underlying physical mechanisms that might be overlooked by traditional analysis methods. Therefore, the large amount of intrinsic property data of materials obtained from high-throughput experiments and the accurate models constructed by machine learning will facilitate the clarification of the impact of RE elements and high-entropy effects on the properties of RE_2_Si_2_O_7_ and the discovery of high-performance wave-transparent RE silicate materials.

In this work, an integrated research framework that combines high-throughput experimental synthesis with machine learning analysis was developed to systematically explore the composition–structure–property relationships in high-entropy RE_2_Si_2_O_7_. Through the independent construction of a high-throughput material synthesis platform, we rapidly prepared and characterized a vast library of (5RE_1/5_)_2_Si_2_O_7_ compositions. Subsequently, a machine learning workflow was developed to extract critical descriptors from multiscale features, identifying the primary factors influencing phase stability, dielectric constant, and thermal conductivity. Furthermore, first-principles calculations were employed to elucidate the underlying mechanisms by which RE cations modulate these properties. Eventually, a 5-RE-principal-component (Ho_1/5_Tm_1/5_Yb_1/5_Lu_1/5_Sc_1/5_)_2_Si_2_O_7_ ceramic was identified, which simultaneously achieves low dielectric constant and low thermal conductivity. This work provides a broader perspective for accelerating the discovery of next-generation wave-transparent ceramics tailored for extreme aerospace environments.

## Results

### High-throughput synthesis and machine learning framework

**Fig. 1.**
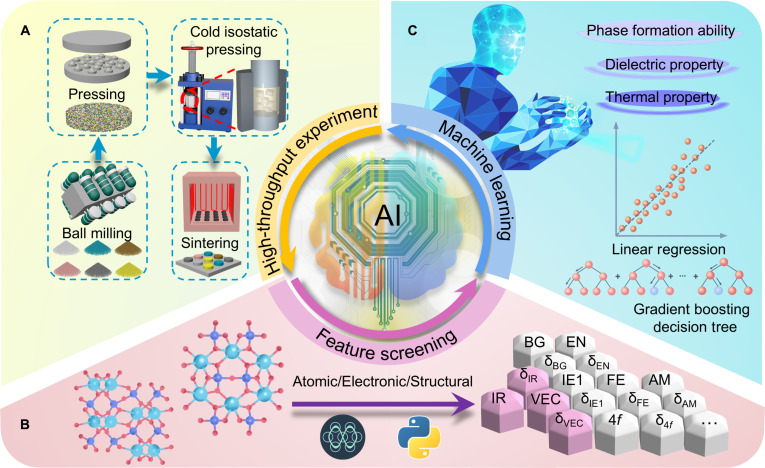
Schematic diagram of collaborative analysis combining high-throughput experiments and machine learning approaches of (5RE_1/5_)_2_Si_2_O_7_. (A) High-throughput synthesis includes high-throughput mixing, pressing, cold isostatic pressing, and sintering. (B) Combining multiscale descriptors to screen out a refined feature set. (C) Machine learning models applied to phase formation ability, dielectric property, and thermal property.

To achieve high-entropy design of RE disilicates (RE_2_Si_2_O_7_), we integrate high-throughput experiments with machine learning techniques to identify promising high-temperature wave-transparent materials. The workflow comprises 3 components: high-throughput experiments, feature screening, and machine learning. In the high-throughput experiments (Fig. 1A), 66 compositions of powders were initially prepared for constructing the phase diagram. Subsequently, based on the phase diagram, 24 β- and γ-phase RE_2_Si_2_O_7_ were selected and fabricated into bulk ceramics. Their comprehensive properties were measured, with dielectric constant and thermal conductivity being used in the machine learning model. Finally, 5 compositions comprising 5, 6, 7, 8, and 9 elements, respectively, were designed to validate the model’s generalization capabilities. All the samples were prepared through a self-built high-throughput platform (Fig. S1). During feature screening (Fig. 1B), 28 descriptors spanning atomic, electronic, and structural scales were selected (Table 1). To ensure feature independence and validity, Pearson correlation analysis was performed for preliminary self-correlation screening. It is particularly noteworthy that descriptors with Pearson correlation coefficients greater than 0.8 are strictly considered highly correlated, a criterion that aids in identifying and excluding features that may lead to multicollinearity issues. Furthermore, Pearson analysis was utilized to evaluate the relationships between these descriptors and target properties (phase composition, dielectric constant, and thermal conductivity), thereby eliminating low-correlation descriptors and refining the feature set. In the machine learning framework (Fig. 1C), leave-one-out cross-validation (LOOCV) was applied to guarantee reliability. The final models implemented include decision tree and linear regression models. Through feature importance analysis within these models, the most strongly correlated descriptors were successfully identified, and their underlying mechanisms are thoroughly investigated.

**Table 1.** Detailed 28 phase formation descriptors of (5RE_1/5_)_2_Si_2_O_7_

Descriptors	Abbreviations	Descriptors	Abbreviations
Average ionic radius	IR	Deviation of ionic radii	δ_IR_
Average valence electron concentration	VEC	Deviation of valence electron concentration	δ_VEC_
Average electronegativity	EN	Deviation of electronegativity	δ_EN_
Average atomic number	AN	Deviation of atomic numbers	δ_AN_
Average atomic mass	AM	Deviation of atomic mass	δ_AM_
Average 4*f* electrons	4*f*	Deviation of 4*f* electrons	δ_4*f*_
Average first ionization energy	IE1	Deviation of first ionization energy	δ_IE1_
Average second ionization energy	IE2	Deviation of second ionization energy	δ_IE2_
Average third ionization energy	IE3	Deviation of third ionization energy	δ_IE3_
Average density	D	Deviation of density	δ_D_
Average bulk modulus	B	Deviation of bulk modulus	δ_B_
Average Fermi energy	FE	Deviation of Fermi energy	δ_FE_
Average band gap	BG	Deviation of band gap	δ_BG_
Average polarizability	P	Deviation of polarizability	δ_P_

### Machine-learning-driven phase diagram construction of (5RE_1/5_)_2_Si_2_O_7_

**Fig. 2.**
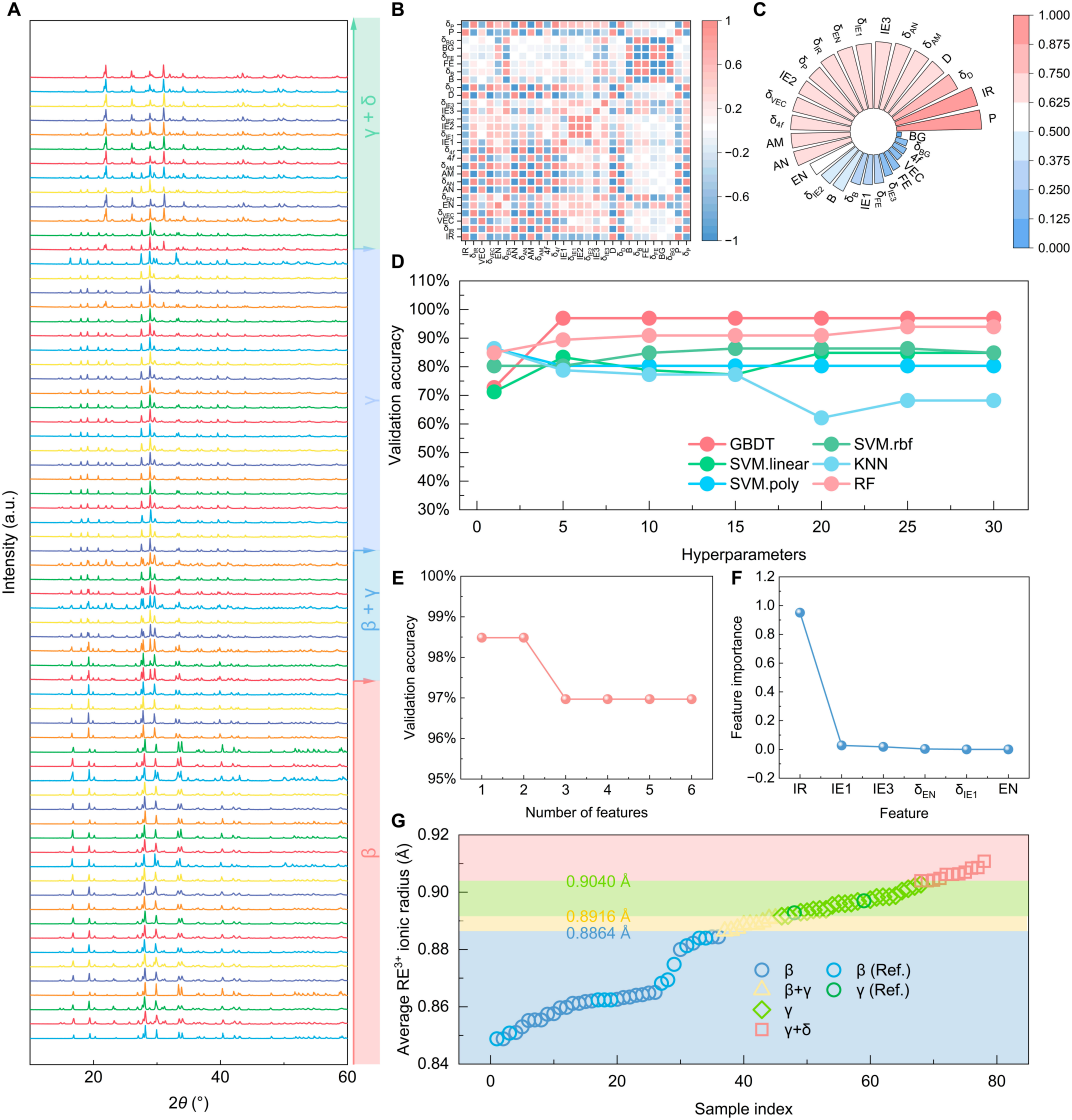
Phase formation ability of (5RE_1/5_)_2_Si_2_O_7_. (A) X-ray diffraction (XRD) patterns of (5RE_1/5_)_2_Si_2_O_7_. (B) Pearson correlation coefficient map of the initial 28 features. (C) The correlation analysis between descriptors and phase composition. (D) Best validation accuracy of 6 machine learning models as a function of hyperparameters. (E) Validation accuracy as a function of feature number. (F) Feature importance of the Gradient Boosting Decision Tree model. (G) The relationship between phase composition and average RE^3+^ ionic radius.

RE_2_Si_2_O_7_ exhibits 7 different crystal polymorphs (β-, α-, γ-, δ-, F-, G-, and A-phases), with β- and γ-phases showing particular promise for high-temperature applications due to their stable structures with no phase transformation. However, the key parameters that govern polymorphic structures remain unclear. A comprehensive sample library consisting of 66 different compositions of (5RE_1/5_)_2_Si_2_O_7_ (RE = Sc, Gd, Tb, Dy, Ho, Er, Tm, Yb, and Lu), primarily focusing on heavy RE elements with a wide range of ionic radii from 0.74 to 0.94 Å, was systematically designed to investigate the polymorphic structures. Multiple sample sets were synthesized using high-throughput experimental methods. The XRD patterns for all prepared (5RE_1/5_)_2_Si_2_O_7_ samples are shown in Fig. 2A. The (5RE_1/5_)_2_Si_2_O_7_ crystallized into β-, γ-, and δ-phases, with some compositions exhibiting multiphase coexistence.

Pearson correlation analysis was employed to thoroughly examine the self-correlation among these descriptors and the correlation between descriptors and phase formation, ensuring their independence and effectiveness. The analysis results are presented in the form of a correlation matrix in Fig. 2B. The specific results of the correlation analysis between descriptors and phase composition can be found in Fig. 2C. By combining the results of self-correlation analysis with the correlations between descriptors and phase composition, a comprehensive evaluation and comparison were conducted, ultimately selecting and identifying the 6 most critical primary descriptors. These 6 descriptors (IR, EN, δ_EN_, IE1, δ_IE1_, and IE3) were selected as input features for the subsequent models. Detailed information about these 6 descriptors for the 66 compositions can be found in Table S1. In order to classify the phase compositions of RE_2_Si_2_O_7_, 6 machine learning models were employed, namely, Gradient Boosting Decision Tree (GBDT), Random Forest (RF), Support Vector Machine (SVM) with different kernels (linear, polynomial [poly], and radial basis function [rbf]), and k-Nearest Neighbors (KNN). Figure 2D illustrates the relationship between prediction accuracy and hyperparameter settings for different machine learning models. By comparing the prediction accuracies of these models under various hyperparameter configurations, GBDT was found to perform the best among all models, achieving the highest accuracy of 96.97%. Further analysis of the optimal GBDT model yielded an excellent weighted precision of 98.54%, listed in Table S2. Figure 2E illustrates the relationship between the number of features and validation accuracy in the GBDT model. The results demonstrate that when the number of descriptors is reduced to only 1 or 2, the validation accuracy can reach up to 98.48%. This indicates that a particular descriptor possesses exceptionally high importance. Figure 2F presents the feature importance analysis results of the GBDT model, further indicating that IR has the most dominant impact on phase composition, with an importance of 95.08%. Figure 2G displays the relationship between phase composition and ionic radius for the samples, including those from this work and those from the referenced literature [32–37]. The results show that (5RE_1/5_)_2_Si_2_O_7_ tends to form the β-phase when the average RE^3+^ ionic radius is below 0.8864 Å and tends to form a mixture of β-phase and γ-phase when the average RE^3+^ ionic radius ranges from 0.8864 to 0.8916 Å; as the average RE^3+^ ionic radius increases to between 0.8916 and 0.9040 Å, the γ-phase becomes more dominant; and when the average RE^3+^ ionic radius exceeds 0.9040 Å, a mixture of γ-phase and δ-phase is more likely to form. It is worth noting that a narrow gap of 0.0052 Å exists between the β-phase boundary (<0.8864 Å) and the γ-phase boundary (>0.8916 Å). The polymorphic phase transformation in high-entropy ceramics is intricately governed by the interplay between thermodynamic stability and diffusion kinetics. As an intrinsic characteristic of these multicomponent systems, the size variations and chemical mismatches among diverse RE^3+^ cations collectively induce severe lattice distortion, which markedly elevates the migration energy. When the average RE^3+^ ionic radius falls precisely within this critical thermodynamic boundary region, the driving force for phase transformation becomes exceptionally weak. The coupling of this marginal thermodynamic driving force with the inherent sluggish diffusion kinetics prevents the system from completely overcoming the energy barrier to transform into a purely single-phase structure under conventional synthesis conditions, thereby trapping it in a multiphase β+γ coexistence [38]. Based on the above analysis, it is evident that the average RE^3+^ ionic radius is a key factor in determining the phase composition of high-entropy RE_2_Si_2_O_7_, thereby laying a solid foundation for further exploring the relationship between material composition and performance.

### Phase composition and microstructure of (5RE_1/5_)_2_Si_2_O_7_

**Fig. 3.**
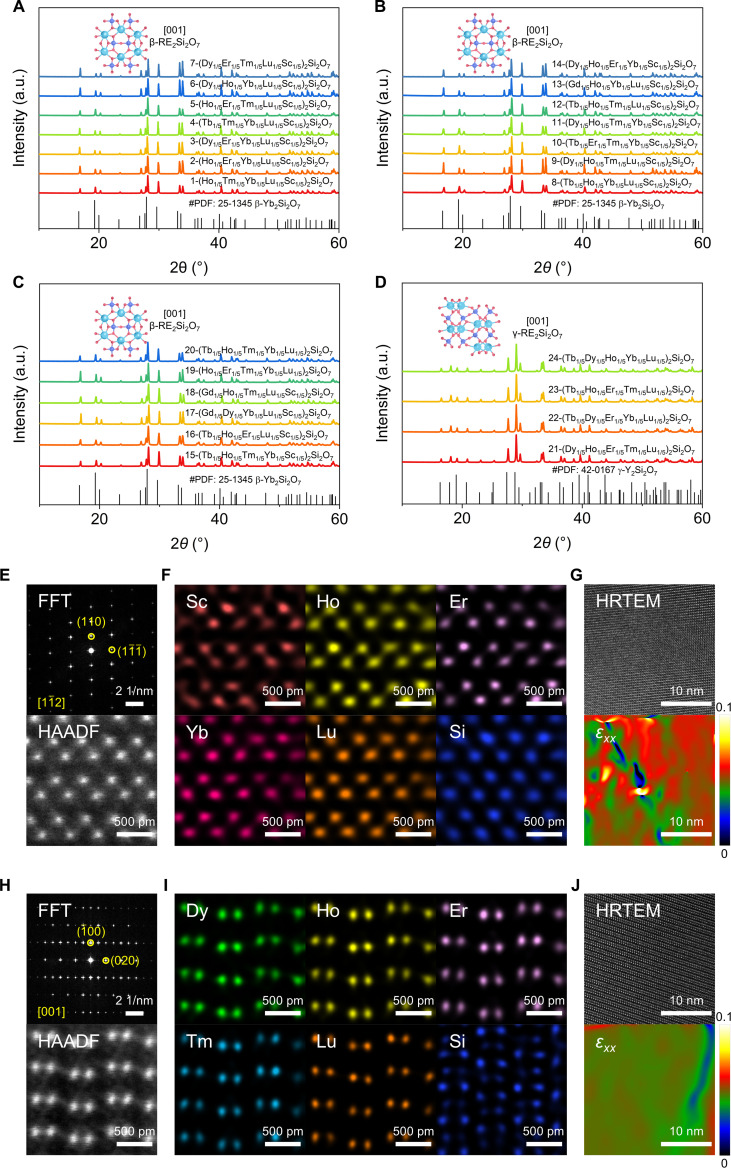
Phase composition and microstructure of (5RE_1/5_)_2_Si_2_O_7_. (A–D) XRD patterns of bulk (5RE_1/5_)_2_Si_2_O_7_. (E and H) Fast Fourier transform (FFT) of the high-angle annular dark-field–scanning transmission electron microscopy (HAADF-STEM) images and HAADF-STEM images of β-(Ho_1/5_Er_1/5_Yb_1/5_Lu_1/5_Sc_1/5_)_2_Si_2_O_7_ and γ-(Dy_1/5_Ho_1/5_Er_1/5_Tm_1/5_Lu_1/5_)_2_Si_2_O_7_. (F and I) Corresponding atomic-resolution energy dispersive spectroscopy (EDS) elemental maps. (G and J) High-resolution transmission electron microscopy (HRTEM) images with corresponding geometric phase analysis (GPA) maps.

Owing to the superior high-temperature stability of β- and γ-phase RE_2_Si_2_O_7_, we selected 24 compositions and fabricated bulk samples based on the above results. The x-ray diffraction (XRD) patterns of these samples are presented in Fig. 3A to D. Rietveld refinements of XRD are provided in Figs. S2 to S4, where the well-refined lattice parameters are compared with density functional theory (DFT) calculation results listed in Table S3, confirming that single-phase β- and γ-RE_2_Si_2_O_7_ have been successfully synthesized and validating the reliability of the subsequent theoretical calculations. Figure S5 presents the fracture morphology of the bulk samples. The micrographs indicate a high densification, evidenced by the presence of few pores. Remarkably, the fracture surfaces display step-like patterns characteristic of cleavage fracture, which can be attributed to the heterogeneous bond energy distribution within the [REO_6_] polyhedra of RE_2_Si_2_O_7_. Under applied stress, the weaker RE–O bonds preferentially rupture, leading to transgranular fracture along specific crystallographic planes. This process results in the formation of flat platforms, while the simultaneous occurrence of cleavage on different crystal planes with identical Miller indices manifests as the observed step-like patterns.

To further verify the atomic structures of the as-synthesized bulk (5RE_1/5_)_2_Si_2_O_7_, we conducted high-angle annular dark-field (HAADF) imaging using scanning transmission electron microscopy (STEM). Figure 3E and H present atomic-resolution HAADF-STEM images of β-(Ho_1/5_Er_1/5_Yb_1/5_Lu_1/5_Sc_1/5_)_2_Si_2_O_7_ viewed along the [$1\;\bar{1}\;2$] zone axis and the γ-(Dy_1/5_Ho_1/5_Er_1/5_Tm_1/5_Lu_1/5_)_2_Si_2_O_7_ along [001], with fast Fourier transform (FFT) patterns matching the respective β and γ polymorphs. Both β- and γ-phase RE_2_Si_2_O_7_ share a skeleton of corner-sharing double [SiO_4_] tetrahedra ([Si_2_O_7_] units) alternating with layers of 6-coordinated RE cations that form cage-like [REO_6_] polyhedra. In both polymorphs, the bridging Si–O–Si angle is linear (180°). Their principal difference is the stacking orientation of the [Si_2_O_7_] units: β-RE_2_Si_2_O_7_ adopts a sandwich-like arrangement with Si–O–Si chains lying in the (0 0 1) planes, whereas γ-RE_2_Si_2_O_7_ shows a wave-like succession of [REO_6_] and [Si_2_O_7_] slabs with these chains parallel to the (1 0 1) planes. The energy dispersive spectroscopy (EDS) maps in Fig. 3F and I show that the selected elements are uniformly distributed in an equimolar (1:1:1:1:1) ratio, as listed in Table S4. These results confirm the successful production of β- and γ-(5RE_1/5_)_2_Si_2_O_7_. Figure 3G and J show high-resolution transmission electron microscopy (HRTEM) images and geometric phase analysis (GPA) maps of β-(Ho_1/5_Er_1/5_Yb_1/5_Lu_1/5_Sc_1/5_)_2_Si_2_O_7_ and γ-(Dy_1/5_Ho_1/5_Er_1/5_Tm_1/5_Lu_1/5_)_2_Si_2_O_7_. Compared with γ-(Dy_1/5_Ho_1/5_Er_1/5_Tm_1/5_Lu_1/5_)_2_Si_2_O_7_, the elastic strains of β-(Ho_1/5_Er_1/5_Yb_1/5_Lu_1/5_Sc_1/5_)_2_Si_2_O_7_ are greater, indicating the occurrence of severe lattice distortion.

### Machine-learning-driven optimization of dielectric and thermal properties of (5RE_1/5_)_2_Si_2_O_7_

**Fig. 4.**
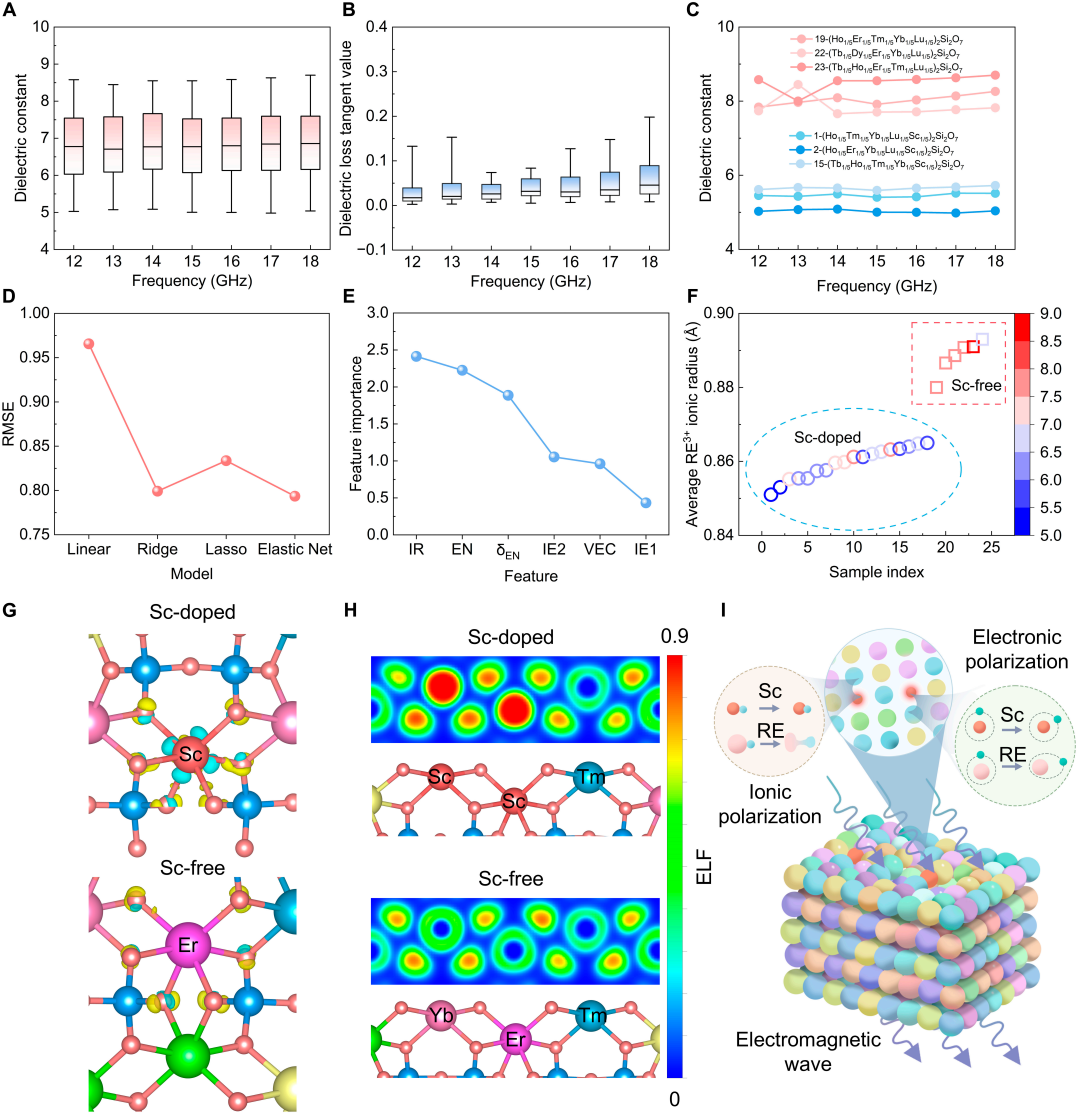
Dielectric properties of (5RE_1/5_)_2_Si_2_O_7_. (A) Dielectric constants of 24 β- and γ-phase samples. (B) Dielectric loss tangent values of 24 β- and γ-phase samples. (C) Dielectric constants of sample 1-(Ho_1/5_Tm_1/5_Yb_1/5_Lu_1/5_Sc_1/5_)_2_Si_2_O_7_, 2-(Ho_1/5_Er_1/5_Yb_1/5_Lu_1/5_Sc_1/5_)_2_Si_2_O_7_, 15-(Tb_1/5_Ho_1/5_Tm_1/5_Yb_1/5_Sc_1/5_)_2_Si_2_O_7_, 19-(Ho_1/5_Er_1/5_Tm_1/5_Yb_1/5_Lu_1/5_)_2_Si_2_O_7_, 22-(Tb_1/5_Dy_1/5_Er_1/5_Yb_1/5_Lu_1/5_)_2_Si_2_O_7_, and 23-(Tb_1/5_Ho_1/5_Er_1/5_Tm_1/5_Lu_1/5_)_2_Si_2_O_7_. (D) RMSE of the Linear, Ridge, Lasso, and Elastic Net Regression models. (E) Feature importance of Elastic Net model. (F) Relationship between dielectric constant and average RE^3+^ ionic radius. (G) Charge density difference (CDD) of Sc-doped and Sc-free samples. (H) Electron localization function (ELF) of Sc-doped and Sc-free samples. (I) Schematic diagram of how Sc reduces the dielectric constant.

The electromagnetic wave transmission characteristics of materials are fundamentally determined by their dielectric properties, primarily the dielectric constant and dielectric loss tangent value. Ideal wave-transparent materials require both low dielectric constant and low dielectric loss tangent value. Figure 4A and B show the dielectric constant and the dielectric loss tangent values of (5RE_1/5_)_2_Si_2_O_7_ between 12 and 18 GHz. The dielectric constants of (5RE_1/5_)_2_Si_2_O_7_ remain stable and below 9, and the dielectric loss tangent values consistently fall within the 10^−2^ order. Detailed dielectric constants and dielectric loss tangent values for the 24 β- and γ-phase samples are presented in Figs. S6 to S8. Among these samples, 1-(Ho_1/5_Tm_1/5_Yb_1/5_Lu_1/5_Sc_1/5_)_2_Si_2_O_7_, 2-(Ho_1/5_Er_1/5_Yb_1/5_Lu_1/5_Sc_1/5_)_2_Si_2_O_7_, and 15-(Tb_1/5_Ho_1/5_Tm_1/5_Yb_1/5_Sc_1/5_)_2_Si_2_O_7_ exhibit relatively low dielectric constants, while samples 19-(Ho_1/5_Er_1/5_Tm_1/5_Yb_1/5_Lu_1/5_)_2_Si_2_O_7_, 22-(Tb_1/5_Dy_1/5_Er_1/5_Yb_1/5_Lu_1/5_)_2_Si_2_O_7_, and 23-(Tb_1/5_Ho_1/5_Er_1/5_Tm_1/5_Lu_1/5_)_2_Si_2_O_7_ exhibit higher dielectric constants. The dielectric constants of 6 samples are presented in Fig. 4C. To identify the primary factors influencing the dielectric properties of materials, machine learning techniques were employed. Pearson correlation analysis was employed to evaluate the self-correlation among the descriptors as well as the correlation between the descriptors and the dielectric constant. The results of correlation analysis between descriptors, as well as between descriptors and dielectric constants, are presented in Figs. S9 and S10, respectively. Ultimately, 6 descriptors (IR, VEC, EN, δ_EN_, IE1, and IE2) were selected as input features for the subsequent models. Detailed information about these 6 descriptors for the 24 compositions can be found in Table S5. Models including Linear Regression (Linear), Ridge Regression (Ridge), Lasso Regression (Lasso), and Elastic Net Regression (Elastic Net) were utilized. The reliability of the models was validated by comparing the root mean squared error (RMSE), as demonstrated in Fig. 4D. The results indicate that the Elastic Net model exhibited the lowest RMSE and was therefore selected as the final model. Feature importance analysis of the Elastic Net model reveals that IR (average RE^3+^ ionic radius) holds a dominant position in dielectric constants, as shown in Fig. 4E. Subsequently, the relationship between the average RE^3+^ ionic radius and dielectric properties is plotted in Fig. 4F, showing that compositions containing Sc with the lowest ionic radius exhibit relatively lower dielectric constants, while those without Sc possess higher dielectric constants. Based on the above findings, a further investigation was conducted into the pivotal role of Sc in the dielectric properties.

Although categorized as a RE element, Sc is distinguished by its lack of 4*f* electrons and its smallest ionic radius. The incorporation of Sc substantially reduces the dielectric constant because its unique electronic configuration and small size synergistically suppress both ionic and electronic polarization. To elucidate this mechanism, charge density difference (CDD) and electron localization function (ELF) analyses are demonstrated in Fig. 4G and H. The CDD mapping reveals a more pronounced charge transfer within the Sc–O bond compared to the Er–O bond, indicating a stronger covalent character. This is further corroborated by first-principles calculations, which confirm a shorter Sc–O bond length, as detailed in Table S6. This enhanced bond strength inherently restricts the off-center displacement of ions under an external alternating electric field, thereby severely mitigating ionic polarization. Furthermore, the ELF reveals a markedly higher degree of electron localization tightly bound around the Sc atomic cores compared to other RE elements. This robust confinement restrains electron cloud deformation, thereby drastically reducing electronic polarization. The wave-transparent mechanism is schematically depicted in Fig. 4I. To sum up, the synergistic effect of Sc doping effectively minimizes the overall polarizability [39,40]. While the relationship between dielectric loss tangent value and RE species is not obvious, the overall dielectric loss tangent values of (5RE_1/5_)_2_Si_2_O_7_ remain highly stable. In general, Sc-doped compositions demonstrate dielectric constants superior to both Al_2_O_3_ (*ε* ~ 9.6) and Si_3_N_4_ (*ε* ~ 7.9) ceramics, and can approach those of BN (*ε* ~ 4) and SiO_2_ (*ε* ~ 3.8) ceramics. Among these, (Ho_1/5_Tm_1/5_Yb_1/5_Lu_1/5_Sc_1/5_)_2_Si_2_O_7_ and (Ho_1/5_Er_1/5_Yb_1/5_Lu_1/5_Sc_1/5_)_2_Si_2_O_7_—which have the 2 smallest average RE^3+^ ionic radii—exhibit the 2 lowest dielectric constants, 5.4 and 5.0, respectively. Interestingly, our analysis reveals that both the phase structure and the dielectric constant are intrinsically governed by this average RE^3+^ ionic radius. Fundamentally, a smaller average radius leads to globally shorter RE–O bonds. Structurally, these shorter bonds drive the transition toward the more densely packed β phase, restricting local ionic polarizability. Consequently, coupled with the unique suppression of electronic polarization by Sc discussed above, the Sc-doped β-phase compositions exhibit a more pronounced advantage in dielectric performance. These results indicate that Sc-doped high-entropy ceramics show great promise for wave-transparent applications.

**Fig. 5.**
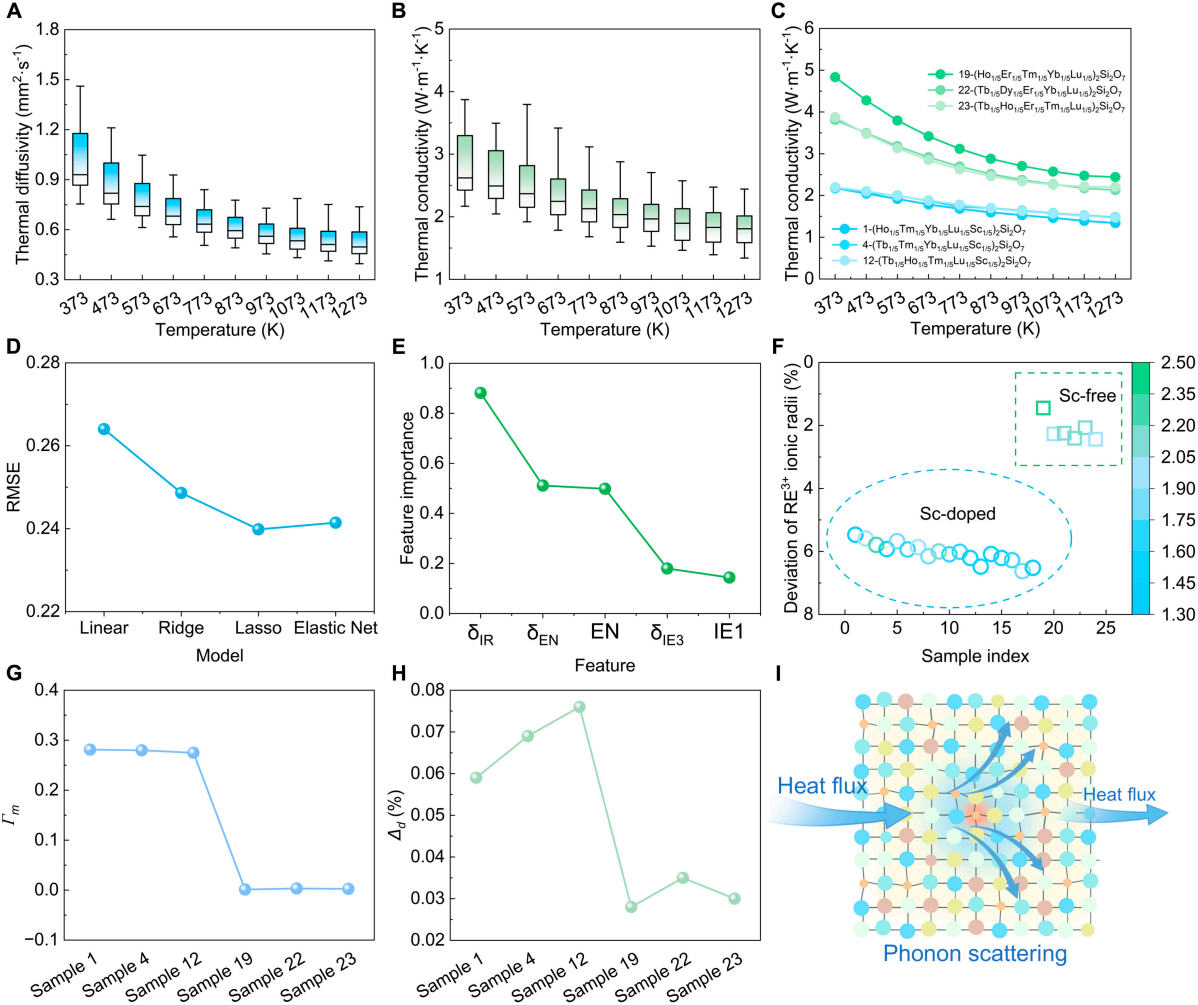
Thermal properties of (5RE_1/5_)_2_Si_2_O_7_. (A) Thermal diffusivities of 24 samples. (B) Thermal conductivities of 24 samples. (C) Thermal conductivities of sample 1-(Ho_1/5_Tm_1/5_Yb_1/5_Lu_1/5_Sc_1/5_)_2_Si_2_O_7_, 4-(Tb_1/5_Tm_1/5_Yb_1/5_Lu_1/5_Sc_1/5_)_2_Si_2_O_7_, 12-(Tb_1/5_Ho_1/5_Tm_1/5_Lu_1/5_Sc_1/5_)_2_Si_2_O_7_, 19-(Ho_1/5_Er_1/5_Tm_1/5_Yb_1/5_Lu_1/5_)_2_Si_2_O_7_, 22-(Tb_1/5_Dy_1/5_Er_1/5_Yb_1/5_Lu_1/5_)_2_Si_2_O_7_, and 23-(Tb_1/5_Ho_1/5_Er_1/5_Tm_1/5_Lu_1/5_)_2_Si_2_O_7_. (D) RMSE of the Linear, Ridge, Lasso, and Elastic Net Regression models. (E) Feature importance of the Lasso model. (F) Relationship between thermal conductivities and deviation of RE^3+^ ionic radii. (G) Mass fluctuation parameters (*Γ*_m_) of sample 1-(Ho_1/5_Tm_1/5_Yb_1/5_Lu_1/5_Sc_1/5_)_2_Si_2_O_7_, 4-(Tb_1/5_Tm_1/5_Yb_1/5_Lu_1/5_Sc_1/5_)_2_Si_2_O_7_, 12-(Tb_1/5_Ho_1/5_Tm_1/5_Lu_1/5_Sc_1/5_)_2_Si_2_O_7_, 19-(Ho_1/5_Er_1/5_Tm_1/5_Yb_1/5_Lu_1/5_)_2_Si_2_O_7_, 22-(Tb_1/5_Dy_1/5_Er_1/5_Yb_1/5_Lu_1/5_)_2_Si_2_O_7_, and 23-(Tb_1/5_Ho_1/5_Er_1/5_Tm_1/5_Lu_1/5_)_2_Si_2_O_7_. (H) Lattice-distortion parameter (Δ*_d_*) of sample 1-(Ho_1/5_Tm_1/5_Yb_1/5_Lu_1/5_Sc_1/5_)_2_Si_2_O_7_, 4-(Tb_1/5_Tm_1/5_Yb_1/5_Lu_1/5_Sc_1/5_)_2_Si_2_O_7_, 12-(Tb_1/5_Ho_1/5_Tm_1/5_Lu_1/5_Sc_1/5_)_2_Si_2_O_7_, 19-(Ho_1/5_Er_1/5_Tm_1/5_Yb_1/5_Lu_1/5_)_2_Si_2_O_7_, 22-(Tb_1/5_Dy_1/5_Er_1/5_Yb_1/5_Lu_1/5_)_2_Si_2_O_7_, and 23-(Tb_1/5_Ho_1/5_Er_1/5_Tm_1/5_Lu_1/5_)_2_Si_2_O_7_. (I) Schematic diagram of mass fluctuation and lattice distortion on enhancing phonon scattering.

To effectively block external heat flux and protect the antenna from thermal damage while ensuring stable transmission of electromagnetic waves, wave-transparent materials should possess low thermal conductivity. Figure 5A and B illustrate the thermal diffusivities and thermal conductivities of (5RE_1/5_)_2_Si_2_O_7_ at various temperatures. At 323 K, the thermal conductivity of (5RE_1/5_)_2_Si_2_O_7_ ranges from approximately 2.0 to 5.1 W·m^−1^·K^−1^. At 1,273 K, the thermal conductivity of (5RE_1/5_)_2_Si_2_O_7_ ranges from approximately 1.3 to 2.5 W·m^−1^·K^−1^. Detailed thermal diffusivities and thermal conductivities for the 24 samples are presented in Figs. S11 to S13. Among these samples, 1-(Ho_1/5_Tm_1/5_Yb_1/5_Lu_1/5_Sc_1/5_)_2_Si_2_O_7_, 4-(Tb_1/5_Tm_1/5_Yb_1/5_Lu_1/5_Sc_1/5_)_2_Si_2_O_7_, and 12-(Tb_1/5_Ho_1/5_Tm_1/5_Lu_1/5_Sc_1/5_)_2_Si_2_O_7_ exhibit relatively low thermal conductivities, while samples 19-(Ho_1/5_Er_1/5_Tm_1/5_Yb_1/5_Lu_1/5_)_2_Si_2_O_7_, 22-(Tb_1/5_Dy_1/5_Er_1/5_Yb_1/5_Lu_1/5_)_2_Si_2_O_7_, and 23-(Tb_1/5_Ho_1/5_Er_1/5_Tm_1/5_Lu_1/5_)_2_Si_2_O_7_ exhibit higher thermal conductivities, as shown in Fig. 5C. Notably, the lowest experimental value of 1.3 W·m^−1^·K^−1^ closely approaches the theoretical minimum thermal conductivity (*κ*_min_) of 1.25 W·m^−1^·K^−1^, which was calculated via the classical Clarke model, with the detailed calculation parameters summarized in Table S7. Machine learning techniques were employed to identify the key factors impacting the thermal properties of materials. Figure S14 presents the Pearson correlation analysis results between descriptors and thermal conductivities. In the end, 5 key descriptors are identified: δ_IR_, EN, δ_EN_, IE1, and IE3. Detailed information about these 5 descriptors for the 24 compositions can be found in Table S8. The same 4 machine learning models as above were adopted (Linear, Ridge, Lasso, and Elastic Net). Figure 5D demonstrates the validation of model reliability through a comparison of RMSE values. Among the models evaluated, the Lasso model achieves the lowest RMSE and is thus chosen as the final model. As shown in Fig. 5E, feature importance analysis of the Lasso model reveals that δ_IR_ (the deviation of RE^3+^ ionic radii) played a dominant role in thermal conductivity.

Subsequently, the relationship between the deviation of RE^3+^ ionic radii and thermal conductivities is presented in Fig. 5F, demonstrating that compositions containing Sc exhibit notably lower thermal conductivities. An intrinsic feature of high-entropy structures is the local chemical fluctuation arising from the mutual interaction of constituent elements. Because Sc possesses a drastically smaller atomic mass and ionic radius compared to other RE elements, its incorporation remarkably amplifies such local chemical fluctuations. To quantitatively elucidate this mechanism, the mass fluctuation (*Γ*_m_) and lattice distortion (Δ*_d_*) of the 6 representative samples in Fig. 5C were calculated, as displayed in Fig. 5G and H, confirming that Sc doping substantially elevates both parameters. These theoretical calculations are further experimentally supported by the Raman spectra shown in Fig. S15, where the Sc-doped composition shows a remarkable peak broadening and continuous overlapping compared to the Sc-free composition. Generally, ideal harmonic crystals exhibit extremely sharp Raman peaks. Although the Sc-free composition displays a certain degree of peak broadening, the incorporation of Sc induces a more pronounced broadening. This phenomenon reveals that the Sc-induced extreme mass fluctuations and severe lattice distortion profoundly disrupt the lattice harmonicity, thereby leading to markedly intensified phonon scattering. Based on the phonon transport theory, the phonon lifetime (*τ*) is inversely proportional to the full width at half maximum (FWHM) of the Raman peak, expressed as [41]:

$$\tau =\frac{1}{2\mathrm{\pi c\Gamma}}$$ (1)

where *c* is the speed of light and *Γ* is the FWHM. Consequently, the observed extensive peak broadening and continuous overlapping provide robust spectroscopic evidence for a substantially shortened phonon lifetime, fully confirming that these pronounced local chemical fluctuations disrupt the long-range periodic arrangement of atoms and induce strong scattering of heat-carrying phonons, ultimately leading to a minimized lattice thermal conductivity in the Sc-doped high-entropy ceramics, as shown in Fig. 5I. It should be clarified that the phase structure has no direct correlation with the thermal conductivity. Crystallographically, despite their distinct stacking modes, both the β- and γ-phases feature identical cage-like [REO_6_] coordination environments and analogous [SiO_4_] structural backbones, which leads to fundamentally equivalent intrinsic phonon scattering mechanisms. As reported in a previous study [42], the thermal conductivities of the β- and γ-phases in RE_2_Si_2_O_7_ are comparable, given identical elemental compositions. Instead, the exceptionally low thermal conductivity achieved in our materials is dominated by the severe lattice distortions and mass fluctuations inherently introduced by the high-entropy compositional design.

To validate the generalization capability of the aforementioned machine learning model, we designed 5 groups of high-entropy RE_2_Si_2_O_7_ compositions comprising 5 [(Er_1/5_Tm_1/5_Yb_1/5_Lu_1/5_Sc_1/5_)_2_Si_2_O_7_], 6 [(Ho_1/6_Er_1/6_Tm_1/6_Yb_1/6_Lu_1/6_Sc_1/6_)_2_Si_2_O_7_], 7 [(Dy_1/7_Ho_1/7_Er_1/7_Tm_1/7_Yb_1/7_Lu_1/7_Sc_1/7_)_2_Si_2_O_7_], 8 [(Tb_1/8_Dy_1/8_Ho_1/8_Er_1/8_Tm_1/8_Yb_1/8_Lu_1/8_Sc_1/8_)_2_Si_2_O_7_], and 9 [(Gd_1/9_Tb_1/9_Dy_1/9_Ho_1/9_Er_1/9_Tm_1/9_Yb_1/9_Lu_1/9_Sc_1/9_)_2_Si_2_O_7_] components, respectively, based on the previously identified key descriptors. Specifically, the dielectric properties are governed by the average RE^3+^ ionic radius, and the thermal conductivity is primarily influenced by the deviation of RE^3+^ ionic radii. The experimental dielectric constants and thermal conductivities, together with the predicted values, are shown in Fig. S16, thereby confirming the model’s generalization ability. This successful validation demonstrates that the machine learning framework, built upon the key descriptors of average ionic radius and deviation of ionic radii, is not only accurate for the 5-component systems but also effectively extendable to more complex compositions with up to 9 distinct RE cations. The establishment of the model facilitates the development of high-entropy RE_2_Si_2_O_7_ wave-transparent materials with superior wave-transparency and thermal-insulation performance.

### Comprehensive properties of (5RE_1/5_)_2_Si_2_O_7_

For wave-transparent materials, the coefficient of thermal expansion plays a crucial role in maintaining structural stability across varying temperature conditions. Figure S17 displays the coefficients of thermal expansion of (5RE_1/5_)_2_Si_2_O_7_, derived from linear fitting of the thermal expansion curves, presented in Figs. S18 to S20. The coefficients of thermal expansion range between 3.3 × 10^−6^ K^−1^ and 4.4 × 10^−6^ K^−1^. The coefficients of thermal expansion of (5RE_1/5_)_2_Si_2_O_7_ show insensitivity to RE species. Previous research [43] has reported that [SiO_4_] tetrahedra in RE_2_Si_2_O_7_ have near-zero thermal expansion coefficients, while [REO_6_] polyhedra dominate thermal expansion behavior. Hazen and Prewitt [44] found that thermal expansion of different crystal structures is primarily determined by the coordination number of RE–O. The RE–O coordination number is identical in both β- and γ-phases, resulting in similar thermal expansion coefficients for these 2 phases and insensitivity to RE species. Becerro et al. used high-temperature x-ray methods to measure the thermal expansion coefficients of several pure-phase RE_2_Si_2_O_7_. The thermal expansion coefficients of β-Lu_2_Si_2_O_7_, β-Yb_2_Si_2_O_7_, β-Er_2_Si_2_O_7_, β-Y_2_Si_2_O_7_, γ-Y_2_Si_2_O_7_, and γ-Ho_2_Si_2_O_7_ were approximately 4 × 10^−6^ K^−1^, while that of β-Sc_2_Si_2_O_7_ was 5.5 × 10^−6^ K^−1^. The (5RE_1/5_)_2_Si_2_O_7_ exhibits lower thermal expansion coefficients than β-Sc_2_Si_2_O_7_ due to marked lattice distortion caused by multi-RE-principal-component doping. Such distortion increases low-frequency vibration modes of RE atoms, enhancing the proportion of phonons contributing to negative expansion within the lattice, thus diminishing the thermal expansion coefficient [45].

Hardness is a crucial property for wave-transparent materials, as it quantifies their resistance to external damage. Figure S21 illustrates the Vickers hardness of (5RE_1/5_)_2_Si_2_O_7_, which range from approximately 5 to 10 GPa under higher loads. Compared to single-component RE_2_Si_2_O_7_ such as Yb_2_Si_2_O_7_ and Lu_2_Si_2_O_7_, the hardness exhibits an enhancement. The improved hardness in high-entropy RE_2_Si_2_O_7_ may arise from strain field fluctuations induced by lattice distortion, thereby enhancing the resistance to deformation. Notably, the Vickers hardness exhibits an inverse relationship with applied load, with smaller loads yielding higher hardness values due to the indentation size effect. Analysis of the Vickers hardness against the RE species under various loads reveals no clear correlation. The hardness of the 24 samples under different loads is specifically plotted in Figs. S22 to S24.

The radar chart facilitates easy comparison of all of the attributes of interest. Detailed comprehensive properties of the 24 samples are presented in Figs. S25 to S27. Aided by radar chart analysis, the (Ho_1/5_Tm_1/5_Yb_1/5_Lu_1/5_Sc_1/5_)_2_Si_2_O_7_ high-entropy ceramic was identified as the optimal candidate. As illustrated in Fig. S28, a direct comparison with mainstream wave-transparent materials (e.g., Al_2_O_3_, Si_3_N_4_, BN, and SiO_2_) demonstrates that our design effectively mitigates the traditional trade-off inherent in structural–functional integration. Specifically, Al_2_O_3_ and Si_3_N_4_ are limited by their high dielectric constants, BN suffers from poor mechanical properties, and SiO_2_ exhibits an excessively low coefficient of thermal expansion that severely hinders structural matching. In contrast, this Sc-doped ceramic exhibits a low dielectric constant, a low dielectric loss tangent value, low thermal conductivity, good hardness, and moderate thermal expansion coefficients, making it a highly competitive candidate for demanding high-temperature wave-transparent applications. Overall, the introduction of Sc markedly reduces both the dielectric constant and thermal conductivity by decreasing ionic and electronic polarization and enhancing phonon scattering through mass fluctuation and lattice distortion. Therefore, high-throughput experimental methods combined with machine learning approaches facilitate the systematic investigation of the synthesis and properties of RE-based high-entropy wave-transparent ceramics.

## Conclusion

In summary, we combined high-throughput methods with machine learning approaches to investigate the composition–structure–property relationships of high-entropy (5RE_1/5_)_2_Si_2_O_7_. The relationship between the average RE^3+^ ionic radius and phase composition was constructed, determining that (5RE_1/5_)_2_Si_2_O_7_ predominantly forms a stable β-phase when the average RE^3+^ ionic radius is below 0.8864 Å; as the average RE^3+^ ionic radius exceeds 0.8916 Å, it gradually transitions to a pure γ-phase. In addition, Sc incorporation markedly reduced the dielectric constant (as low as 5 at 15 GHz) and the thermal conductivity (as low as 1.3 W·m^−1^·K^−1^ at 1,273 K). Further first-principles calculations confirm that the underlying mechanisms include the following: (a) the small ionic radius and electron localization of Sc reduce both ionic and electronic polarization, thereby lowering the dielectric constant; (b) the large ionic radius and mass mismatch between Sc and other RE atoms induce considerable lattice distortion and mass fluctuation, which enhance phonon scattering and consequently reduce the thermal conductivity. Furthermore, the machine learning model can be extended to 9-RE-principal-component RE_2_Si_2_O_7_. Although the dielectric loss tangent value, Vickers hardness, and thermal expansion coefficient show no strict correlation with specific RE species, several important conclusions can be drawn through experiments: dielectric loss remains relatively stable across compositions and high-entropy compositions demonstrate lower coefficients of thermal expansion than their single-component counterparts. Overall, the (Ho_1/5_Tm_1/5_Yb_1/5_Lu_1/5_Sc_1/5_)_2_Si_2_O_7_ high-entropy ceramic that satisfies the above criterion jointly achieves low dielectric constant and low thermal conductivity, and exhibits excellent comprehensive properties, underscoring its potential for wave-transparent applications. The introduction of machine learning methods successfully and efficiently analyzed the large volumes of data generated by high-throughput experiments, deepening the understanding of intrinsic mechanisms and providing a broader range of material options and more comprehensive theoretical guidance for the development of novel high-performance wave-transparent materials.

## Methods

### Sample preparation

A high-throughput method was employed to prepare high-entropy RE_2_Si_2_O_7_. First, RE_2_O_3_ powders (>99.9%, Guangzhou Jianfeng Rare Earth Co. Ltd., China) and SiO_2_ powders (99.7%, Sinopharm Chemical Reagent Co. Ltd., China) were mixed in a molar ratio of nRE_2_O_3_:SiO_2_ = 1:2, with each RE_2_O_3_ added in equal molar proportions. In order to compensate for SiO_2_ volatilization at elevated temperatures, 2% excess SiO_2_ was added. The mixed powders were ball-milled at 70 r/min for 2 h in a high-throughput polyethylene jar, using agate balls and anhydrous ethanol as the milling medium. After being dried at 80 °C for 6 h, the powders were sieved through a 60-mesh screen. The fine powders were sintered at 1,700 °C for 3 h (heating rate: 5 °C/min; cooling rate: 2 °C/min) to obtain high-entropy RE_2_Si_2_O_7_ in a muffle furnace. The high-entropy RE_2_Si_2_O_7_ was further ball-milled using the same method as above. The as-prepared powders were uniaxially pressed under a uniform pressure of 5 MPa using a high-throughput mold capable of simultaneously fabricating up to 32 samples, followed by cold isostatic pressing at 200 MPa for 15 min. Finally, the bulk samples were sintered at 1,600 °C for 20 h (heating rate: 5 °C/min) to obtain dense high-entropy RE_2_Si_2_O_7_ ceramics.

### Characterizations

The density of the sintered bulk samples was determined by the Archimedes’ method. The phase composition was characterized by XRD (Bruker D8 ADVANCE, Germany). The surface grain morphology was observed using a scanning electron microscope (Hitachi SU5000, Japan). The dielectric constant and the dielectric loss tangent of the samples were measured using a vector network analyzer (Keysight N5227B PNA, America) combined with the waveguide method, with sample dimensions of 16 mm × 8 mm × 2 mm. The Vickers hardness was determined using a Vickers hardness tester (Veiyee HV-30AT, China). The thermal expansion coefficient was measured using a pushrod dilatometer (NETZSCH DIL 402 CLASSIC, Germany). The thermal diffusivity was determined using a laser flash analyzer (NETZSCH LFA 427, Germany) with the sample dimensions of a diameter of 6 mm and a thickness of 1 mm. The thermal conductivity (*κ*) can be obtained using the following formula [46]:

$$\kappa =a\times C_{p}\times \rho$$ (2)

where *ρ* is the density and *C_p_* represents the heat capacity. *C_p_* was calculated according to the Neumann–Kopp law.

### Theoretical calculations

The high-entropy structures were generated using the special quasi-random structure (SQS) method implemented in the Alloy Theoretic Automated Toolkit [47,48]. A 5 × 1 × 1 supercell was constructed for calculations. The electronic structure calculations were performed through first-principles simulations within DFT using the Vienna Ab initio Simulation Package (VASP) [49]. The projector augmented wave (PAW) pseudopotentials method was employed to describe the electron–ion interactions. For the exchange and correlation interactions, the generalized gradient approximation (GGA) with the Perdew–Burke–Ernzerhof (PBE) functional was used [50]. The Brillouin zone was sampled using the Monkhorst–Pack scheme with a k-point spacing of 0.3 Å^−1^ [51]. The cutoff energy for the plane wave basis set was set to 520 eV [52]. To ensure convergence, the energy criterion was set to 10^−6^ eV. The charge distribution and transfer in the optimized structures were analyzed using the CDD and ELF. The Young’s modulus (*E*) required for the Clarke model was derived using the energy-strain method. The theoretical minimum thermal conductivity (*κ*_min_) was then calculated according to the Clarke model [53]:

$$\kappa_{\min}=0.87k_{B}{\left(\frac{M}{\mathrm{n\rho}N_{A}}\right)}^{-\frac{2}{3}}{\left(\frac{E}{\rho }\right)}^{\frac{1}{2}}$$ (3)

where *k*_B_ is the Boltzmann constant, *N*_A_ is Avogadro’s constant, *n* is the number of atoms in the primitive cell, *ρ* is the density, *E* is the Young’s modulus, and *M* is the molecular weight.

### Machine learning framework

A comprehensive machine learning approach was developed using Python and the scikit-learn library to predict phase composition and material properties [54,55]. Before model training, the mean and difference values of the considered fundamental physical parameters from the atomic level and the monolithic level were calculated as follows:

$$\mathrm{pam}=\sum_{i=1}^{n}c_{i}{\mathrm{pam}}_{i}$$ (4)

$$\text{δpam}=\sqrt{\sum_{i=1}^{n}c_{i}\left[{\left(1-\frac{{\mathrm{pam}}_{i}}{\sum_{i=1}^{n}c_{i}{\mathrm{pam}}_{i}}\right)}^{2}\right]}$$ (5)

where pam*_i_* and *c_i_* are the considered fundamental physical parameters and the ratios of the *i*th constituents, respectively. Meanwhile, all features were subjected to min–max normalization to scale the values into the range of [0,1], which was calculated as follows:

$$x_{i}^{\mathrm{norm}}=\frac{x_{i}-x_{\min}}{x_{\max}-x_{\min}}$$ (6)

where $x_{i}^{\mathrm{norm}}$ is the *i*th normalized value of the input feature x. $x_{\max}$, $x_{\min}$, and $x_{i}$ are the maximum, minimum, and *i*th value of the input feature x, respectively. In order to reduce potential overfitting introduced by strongly correlated features, the Pearson correlation coefficient (*r*) between features was calculated as:

$$r=\frac{\sum \left(x_{i}-\bar{x}\right)\left(y_{i}-\bar{y}\right)}{\sqrt{\sum {\left(x_{i}-\bar{x}\right)}^{2}\sum {\left(y_{i}-\bar{y}\right)}^{2}}}$$ (7)

where $x_{i}$ and $y_{i}$ are the *i*th values of 2 different input features, respectively. $\bar{x}$ and $\bar{y}$ are the mean values of the 2 different input features, respectively. The prediction framework consisted of multiple machine learning algorithms. For phase formation ability, the implemented algorithms included GBDT, RF, SVM with different kernels (linear, poly, and rbf), and KNN. The training dataset for phase prediction was constructed from 66 synthesized high-entropy samples, comprising 48 single-phase and 18 multiphase samples. For the classification task, the phase structures, namely, β, β+γ, γ, and γ+δ were categorically encoded as “0”, “1”, “2”, and “3”, respectively. The classification performance of these models was assessed by calculating the predictive accuracy via LOOCV. For our multiclass phase classification problem, the overall accuracy (Acc) can be mathematically expressed as:

$$\mathrm{Acc}=\frac{1}{N}\sum {\mathrm{Acc}}_{i}$$ (8)

where *N* and ${\mathrm{Acc}}_{i}$ are the number of folds and the validation accuracy of each fold, respectively. The dataset was split into *N* folds, and the number of folds was equal to the dataset size. In each fold, only one sample was included in the validation set, while the rest belonged to the training set. Thus, Acc*_i_* strictly equals 1 for a correct prediction and 0 for an incorrect one. Furthermore, to account for the inherent class imbalance in the dataset, the weighted precision was also calculated for the optimal model. For dielectric and thermal properties, Linear, Ridge, Lasso, and Elastic Net were applied, and their predictive performance was evaluated using the RMSE derived from LOOCV. Rather than enforcing a consistent model across both properties, we evaluated multiple algorithms and strictly selected the optimal one for each target based on achieving the lowest RMSE. Ultimately, feature importance analysis was conducted across both the classification and regression models, aiming to elucidate the underlying physical mechanisms.

## Data Availability

The data that support the findings of this study are available from the corresponding authors on reasonable request.
