# Supplementary material for: Data-Driven Discovery of Composition–Structure–Property Relationship in Novel Wave-Transparent High-Entropy Rare Earth Disilicate
Source: Research (Wash D C). 2026 Jun 1;9:1308. doi: 10.34133/research.1308 (PMC13224110; doi:10.34133/research.1308)
Supplement: Supplementary 1 — Figs. S1 to S28 Tables S1 to S8 [file research.1308.f1.zip › SupplementaryInformation-0508.pdf]

SUPPLYMENTARY MATERIALS

Data-Driven Discovery of Composition-Structure-Property Relationship in Novel Wave-Transparent High-Entropy Rare Earth Disilicate

Shuping Wen, Zhilin Tian*, Yuhong Du, Lin Chi, Zhilin Chen, Liya Zheng*, and Bin Li*

School of Materials, Shenzhen Campus of Sun Yat-sen University, Shenzhen, 518107, China

*Corresponding authors: Zhilin Tian, Liya Zheng, and Bin Li
E-mail addresses: tianzhlin@mail.sysu.edu.cn (Z.L. Tian); zhengly26@mail.sysu.edu.cn (L.Y. Zheng); libin75@mail.sysu.edu.cn (B. Li)

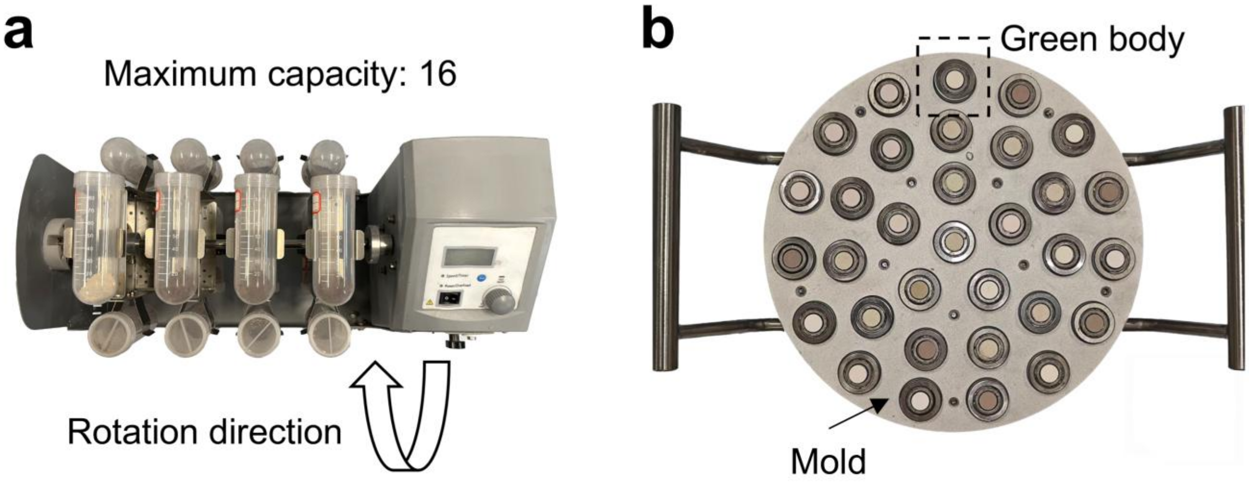

Fig. S1 High-throughput (a) mixing machine, (b) compressing machine.

Table S1 Phase compositions of the as-synthesized 66 samples, with the six formation ability descriptors. (Note: Sample abbreviations such as ErTmYbLuSc represent equimolar $(Er_{1/5}Tm_{1/5}Yb_{1/5}Lu_{1/5}Sc_{1/5})_2Si_2O_7$.)

Index	Sample	IR (Å)	EN	δEN	IE1 (kJ/mol)	δIE1	IE3 (kJ/mol)	Phase
1	ErTmYbLuSc	0.8488	1.244	0.0476	589.2	0.0446	2261.38	β
2	HoTmYbLuSc	0.8510	1.242	0.0496	587.54	0.0481	2263.38	β
3	HoErYbLuSc	0.8530	1.240	0.0484	586.06	0.0462	2245.18	β
4	DyErYbLuSc	0.8552	1.238	0.0504	584.46	0.0496	2244.38	β
5	TbTmYbLuSc	0.8554	1.236	0.0557	584.5	0.0545	2245.38	β
6	HoErTmLuSc	0.8554	1.270	0.0283	584.72	0.0444	2218.78	β
7	DyHoYbLuSc	0.8574	1.236	0.0511	582.8	0.0487	2246.38	β
8	DyErTmLuSc	0.8576	1.268	0.0297	583.12	0.0478	2217.98	β
9	TbHoYbLuSc	0.8596	1.232	0.0539	581.36	0.0508	2229.18	β
10	DyHoTmLuSc	0.8598	1.266	0.031	581.46	0.046	2219.98	β
11	TbErTmYbSc	0.8612	1.230	0.052	597.66	0.0276	2279.72	β
12	DyHoTmYbSc	0.8612	1.232	0.0474	597.44	0.0279	2298.92	β
13	DyHoErLuSc	0.8618	1.264	0.0323	579.98	0.0438	2201.78	β
14	TbHoTmLuSc	0.8620	1.262	0.0336	580.02	0.0488	2202.78	β
15	GdHoYbLuSc	0.8626	1.232	0.0539	586.88	0.0472	2204.38	β
16	DyHoErYbSc	0.8632	1.230	0.0455	595.96	0.0299	2280.72	β
17	TbHoTmYbSc	0.8634	1.228	0.0508	596	0.0303	2281.72	β
18	TbHoErYbSc	0.8640	1.226	0.0496	594.52	0.0319	2263.52	β
19	TbDyTmLuSc	0.8642	1.26	0.0349	578.42	0.0505	2201.98	β
20	GdDyYbLuSc	0.8648	1.23	0.0553	585.28	0.0506	2203.58	β
21	GdHoTmLuSc	0.8650	1.262	0.0336	585.54	0.0455	2177.98	β
22	HoErTmYbLu	0.8800	1.218	0.0388	578.78	0.0382	2224.46	β
23	DyErTmYbLu	0.8822	1.216	0.0382	577.18	0.0401	2223.66	β
24	TbErTmYbLu	0.8844	1.212	0.0409	575.74	0.0432	2206.46	β
25	DyHoTmYbLu	0.8844	1.214	0.0376	575.52	0.0379	2225.66	β
26	DyHoErYbLu	0.8864	1.212	0.037	574.04	0.0359	2207.46	β
27	TbHoTmYbLu	0.8866	1.21	0.0397	574.08	0.041	2208.46	β+γ
28	GdErTmYbLu	0.8874	1.212	0.0409	581.26	0.0397	2181.66	β+γ
29	TbHoErYbLu	0.8886	1.208	0.0384	572.6	0.039	2190.26	β+γ
30	TbDyTmYbLu	0.8888	1.208	0.0384	572.48	0.0389	2207.66	β+γ
31	DyHoErTmLu	0.8888	1.242	0.0116	572.7	0.0344	2181.06	β+γ
32	GdHoTmYbLu	0.8896	1.210	0.0397	579.6	0.0387	2183.66	β+γ
33	TbDyErYbLu	0.8908	1.206	0.0371	571	0.0369	2189.46	β+γ
34	TbHoErTmLu	0.8910	1.238	0.0149	571.26	0.0373	2163.86	β+γ
35	GdHoErYbLu	0.8916	1.208	0.0384	578.12	0.0378	2165.46	γ
36	GdDyTmYbLu	0.8918	1.208	0.0384	578	0.0412	2182.86	γ
37	TbDyHoYbLu	0.8930	1.204	0.0359	569.34	0.0347	2191.46	γ
38	TbDyErTmLu	0.8932	1.236	0.0168	569.66	0.0351	2163.06	γ
39	GdDyErYbLu	0.8938	1.206	0.0371	576.52	0.0392	2164.66	γ
40	GdTbTmYbLu	0.8940	1.204	0.0372	576.56	0.0443	2165.66	γ
41	GdHoErTmLu	0.8940	1.238	0.0149	576.78	0.0369	2139.06	γ
42	TbDyHoTmLu	0.8954	1.234	0.0169	568	0.0329	2165.06	γ
43	GdTbErYbLu	0.8960	1.202	0.0353	575.08	0.0423	2147.46	γ
44	GdDyHoYbLu	0.896	1.204	0.0359	574.86	0.037	2166.66	γ
45	GdDyErTmLu	0.8962	1.236	0.0168	575.18	0.0375	2138.26	γ
46	TbDyHoTmYb	0.8968	1.200	0.0333	583.98	0.022	2244	γ

47	TbDyHoErLu	0.8974	1.232	0.0149	566.52	0.0309	2146.86	γ
48	GdTbHoYbLu	0.8982	1.200	0.0333	573.42	0.0401	2149.46	γ
49	GdTbErTmLu	0.8984	1.232	0.0208	573.74	0.0406	2121.06	γ
50	GdDyHoTmLu	0.8984	1.234	0.0169	573.52	0.0352	2140.26	γ
51	TbDyHoErYb	0.8988	1.198	0.0327	582.5	0.019	2225.8	γ
52	GdDyHoErLu	0.9004	1.232	0.0149	572.04	0.0339	2122.06	γ
53	TbDyHoErTm	0.9012	1.228	0.0117	581.16	0.0163	2199.4	γ
54	GdTbHoTmYb	0.9020	1.196	0.0321	588.06	0.0199	2202	γ
55	GdTbDyTmLu	0.9028	1.228	0.0208	570.48	0.0362	2122.26	γ
56	GdTbHoErYb	0.9040	1.194	0.0315	586.58	0.018	2183.8	γ+δ
57	GdDyHoErTm	0.9042	1.228	0.0117	586.68	0.0132	2174.6	γ+δ
58	GdTbDyErLu	0.9048	1.226	0.0189	569	0.0342	2104.06	γ+δ
59	GdTbDyTmYb	0.9062	1.194	0.0315	586.46	0.0233	2201.2	γ+δ
60	GdTbDyErYb	0.9062	1.192	0.0309	584.98	0.0213	2183	γ+δ
61	GdTbHoErTm	0.9064	1.224	0.0157	585.24	0.0162	2157.4	γ+δ
62	GdTbDyHoLu	0.9070	1.224	0.017	567.34	0.032	2106.06	γ+δ
63	GdTbDyHoYb	0.9084	1.19	0.0303	583.32	0.0207	2185	γ+δ
64	GdTbDyErTm	0.9086	1.222	0.0151	583.64	0.0195	2156.6	γ+δ
65	GdTbDyHoTm	0.9108	1.22	0.0131	581.98	0.018	2158.6	γ+δ
66	GdTbDyHoEr	0.9128	1.218	0.0118	580.5	0.0153	2140.4	γ+δ

Table S2 Classification precision of the GBDT model.

	β	β+γ	γ	γ+δ	Weighted average
Precision	96.43%	100.00%	100.00%	100.00%	98.54%

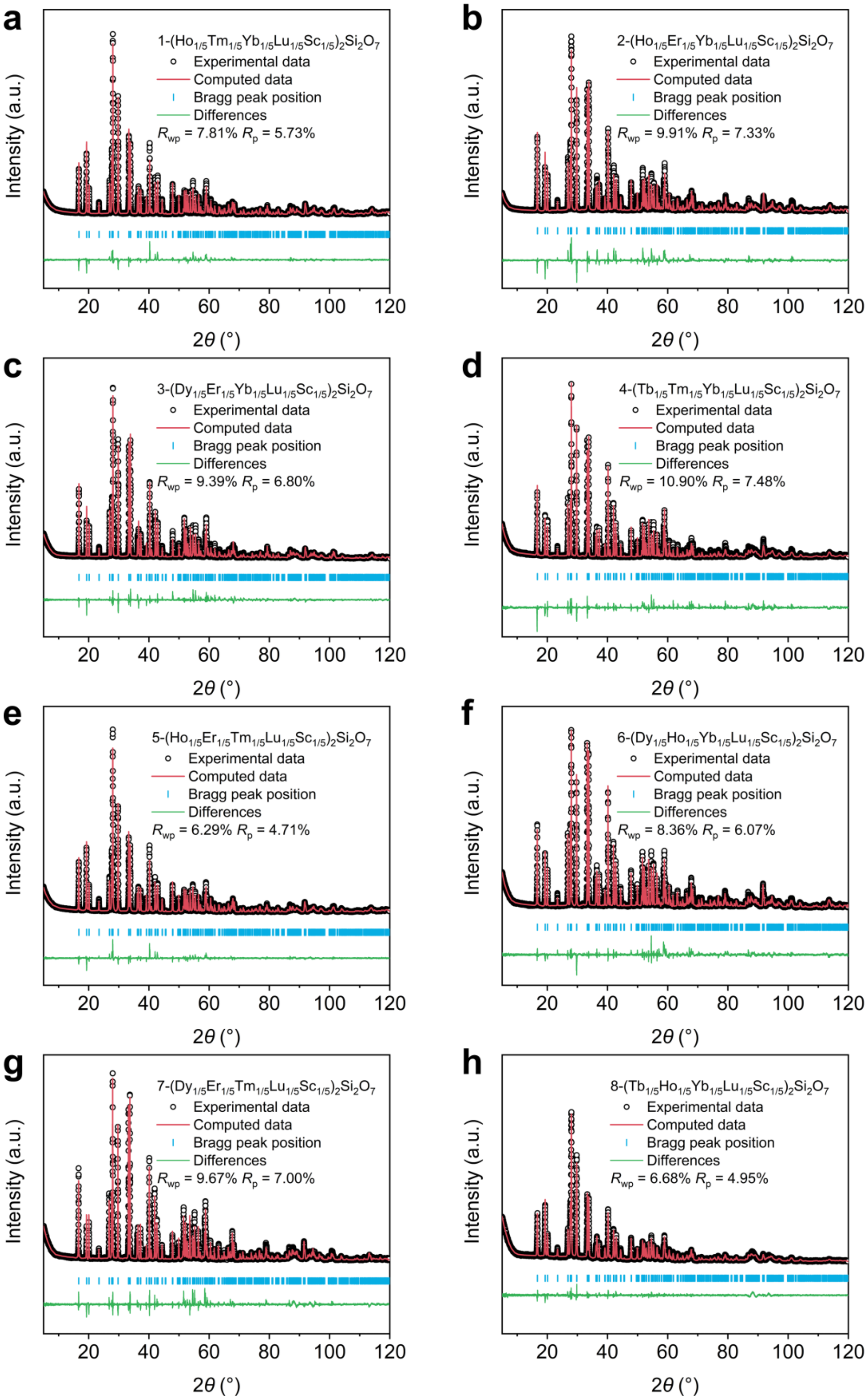

Fig. S2 XRD patterns with Rietveld refinement of $(5RE_{1/5})_2Si_2O_7$ (Samples 1-8).

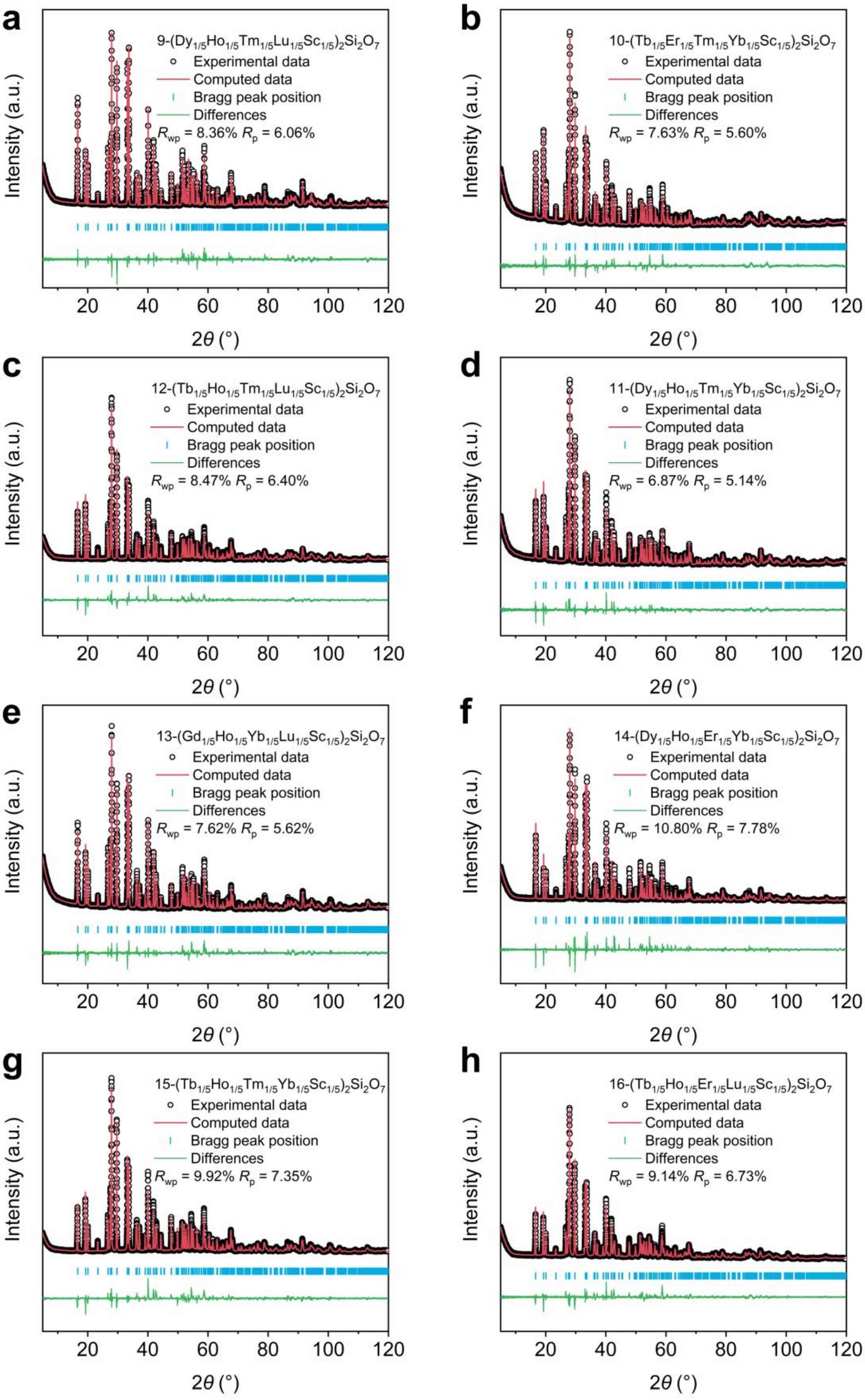

Fig. S3 XRD patterns with Rietveld refinement of $(5RE_{1/5})_2Si_2O_7$ (Samples 9-16).

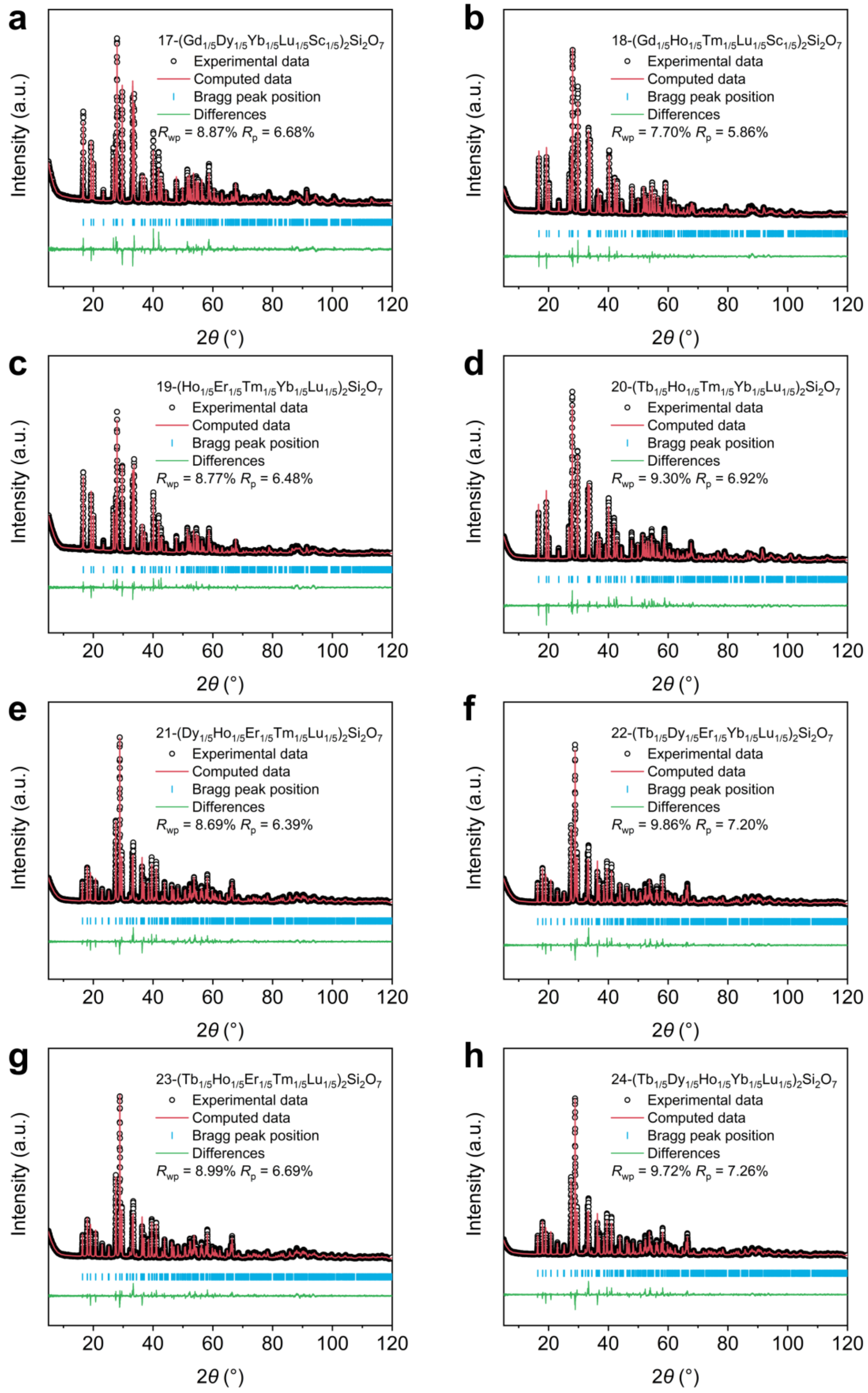

Fig. S4 XRD patterns with Rietveld refinement of $(5RE_{1/5})_2Si_2O_7$ (Samples 17-24).

Table S3 Lattice parameters (a, b, and c) of the as-synthesized 24 bulk samples from XRD and DFT calculations. (Note: Sample abbreviations such as HoTmYbLuSc represent equimolar $(Ho_{1/5}Tm_{1/5}Yb_{1/5}Lu_{1/5}Sc_{1/5})_2Si_2O_7$.)

Index	Sample	a_{XRD} (Å)	a_{cal} (Å)	b_{XRD} (Å)	b_{cal} (Å)	c_{XRD} (Å)	c_{cal} (Å)
1	HoTmYbLuSc	6.765	6.767	8.814	8.860	4.701	4.759
2	HoErYbLuSc	6.771	6.773	8.821	8.869	4.701	4.759
3	DyErYbLuSc	6.766	6.780	8.814	8.877	4.699	4.760
4	TbTmYbLuSc	6.781	6.781	8.823	8.875	4.699	4.759
5	HoErTmLuSc	6.775	6.780	8.826	8.875	4.701	4.759
6	DyHoYbLuSc	6.785	6.786	8.832	8.884	4.701	4.761
7	DyErTmLuSc	6.798	6.786	8.842	8.882	4.699	4.760
8	TbHoYbLuSc	6.781	6.793	8.828	8.892	4.702	4.762
9	DyHoTmLuSc	6.799	6.793	8.849	8.888	4.700	4.760
10	TbErTmYbSc	6.786	6.799	8.827	8.897	4.698	4.761
11	DyHoTmYbSc	6.792	6.798	8.841	8.896	4.699	4.761
12	TbHoTmLuSc	6.801	6.800	8.845	8.894	4.703	4.761
13	GdHoYbLuSc	6.798	6.801	8.844	8.900	4.702	4.762
14	DyHoErYbSc	6.793	6.805	8.838	8.905	4.698	4.763
15	TbHoTmYbSc	6.809	6.806	8.855	8.904	4.703	4.762
16	TbHoErLuSc	6.785	6.806	8.832	8.906	4.701	4.763
17	GdDyYbLuSc	6.805	6.808	8.850	8.908	4.703	4.763
18	GdHoTmLuSc	6.762	6.808	8.809	8.901	4.699	4.761
19	HoErTmYbLu	6.803	6.823	8.847	8.942	4.702	4.769
20	TbHoTmYbLu	6.797	6.843	8.845	8.965	4.702	4.771
21	DyHoErTmLu	4.682	4.746	10.816	10.793	5.570	5.577
22	TbDyErYbLu	4.683	4.746	10.818	10.807	5.573	5.581
23	TbHoErTmLu	4.685	4.746	10.819	10.805	5.574	5.582
24	TbDyHoYbLu	4.686	4.746	10.821	10.817	5.576	5.586

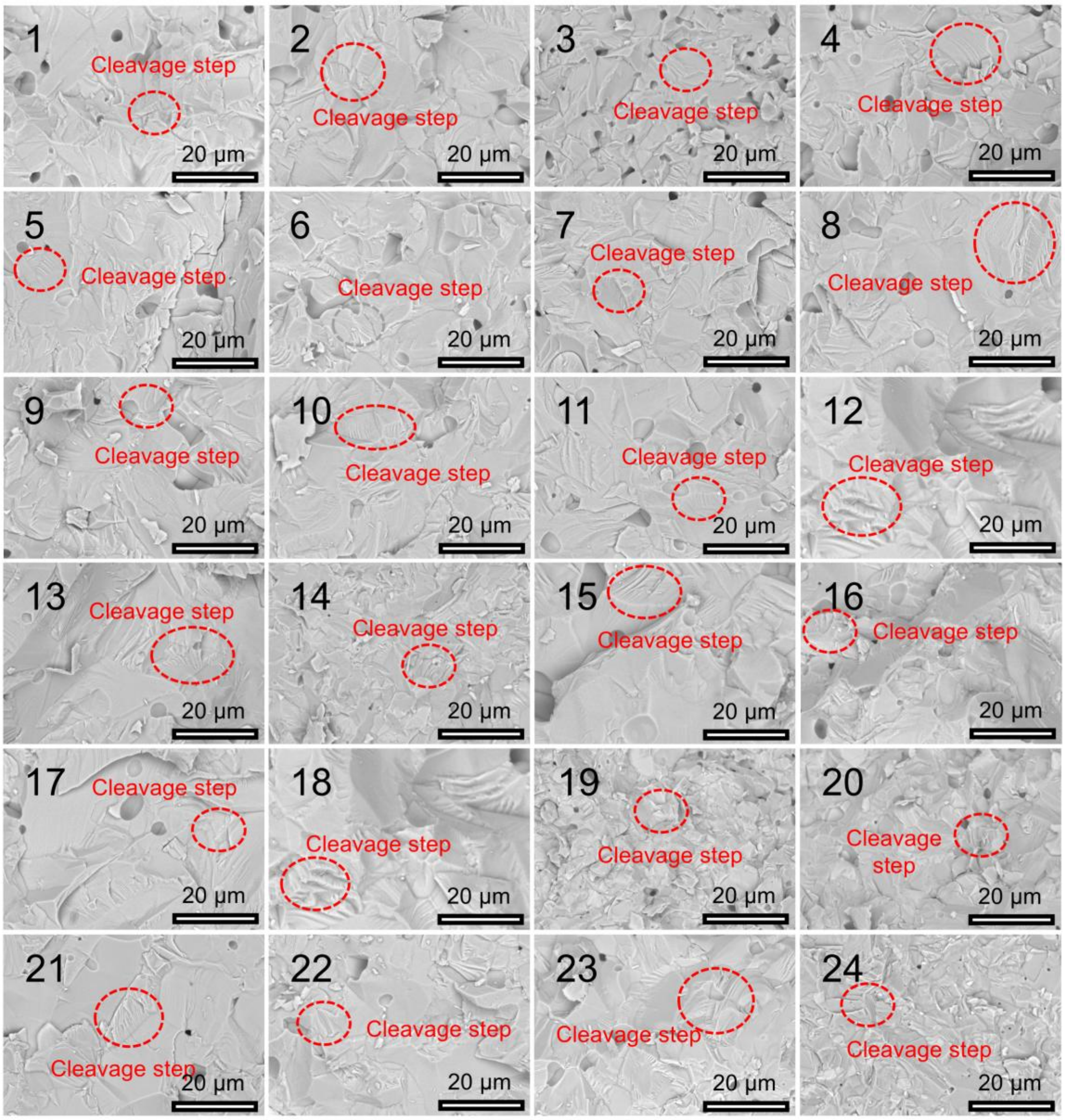

Fig. S5 Fracture surfaces SEM images of bulk $(5RE_{1/5})_2Si_2O_7$.

Table S4 Atomic ratios of RE elements (in at. %) of $(Ho_{1/5}Er_{1/5}Yb_{1/5}Lu_{1/5}Sc_{1/5})_2Si_2O_7$ and $(Dy_{1/5}Ho_{1/5}Er_{1/5}Tm_{1/5}Lu_{1/5})_2Si_2O_7$.

Sample	Dy	Ho	Er	Tm	Yb	Lu	Sc
$(Ho_{1/5}Er_{1/5}Yb_{1/5}Lu_{1/5}Sc_{1/5})_2Si_2O_7$	\	21.34	22.50	\	18.45	15.74	21.95
$(Dy_{1/5}Ho_{1/5}Er_{1/5}Tm_{1/5}Lu_{1/5})_2Si_2O_7$	19.41	17.97	21.10	22.47	\	19.03	\

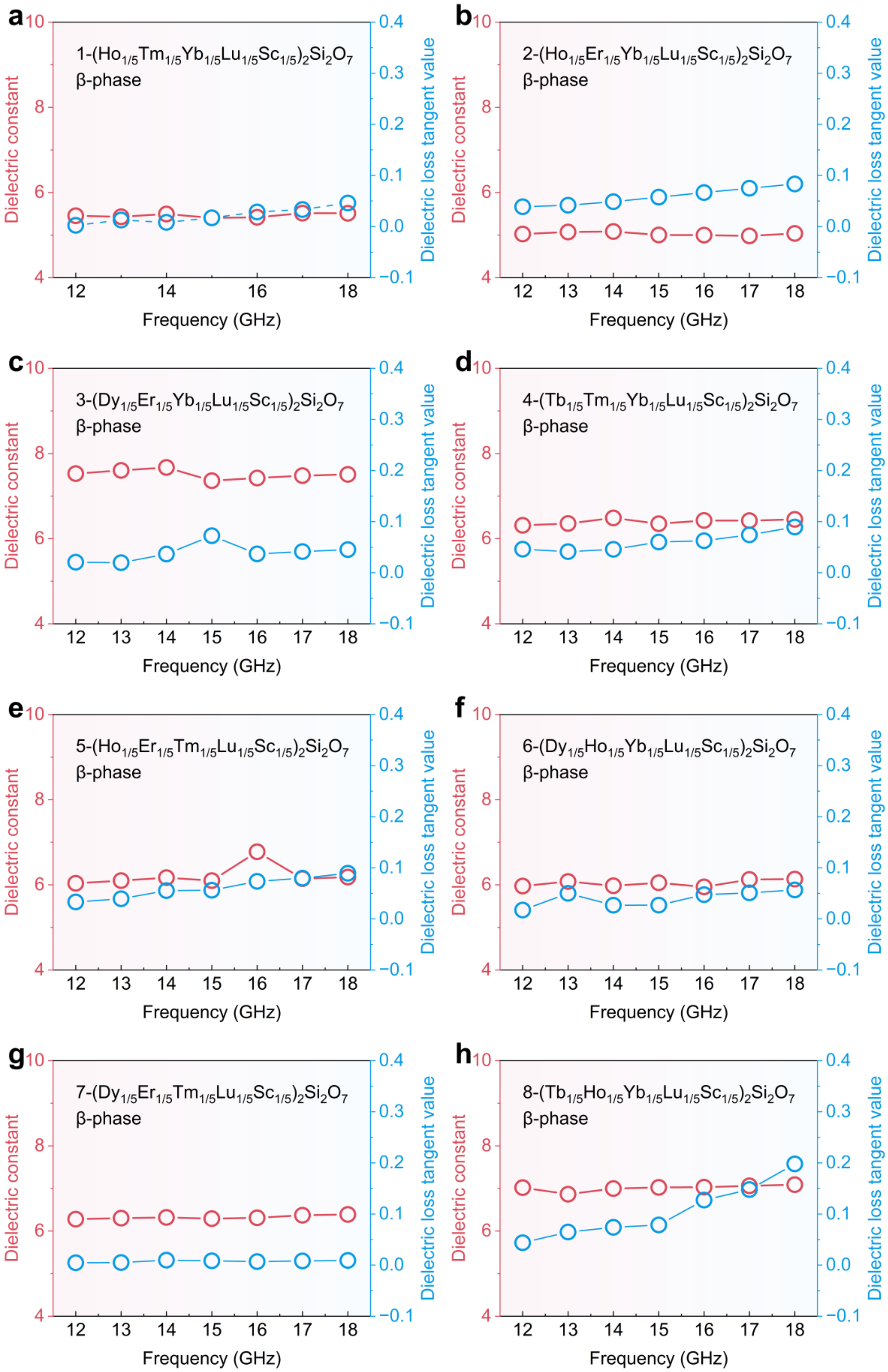

Fig. S6 Dielectric properties of $(5RE_{1/5})_2Si_2O_7$ (Samples 1-8).

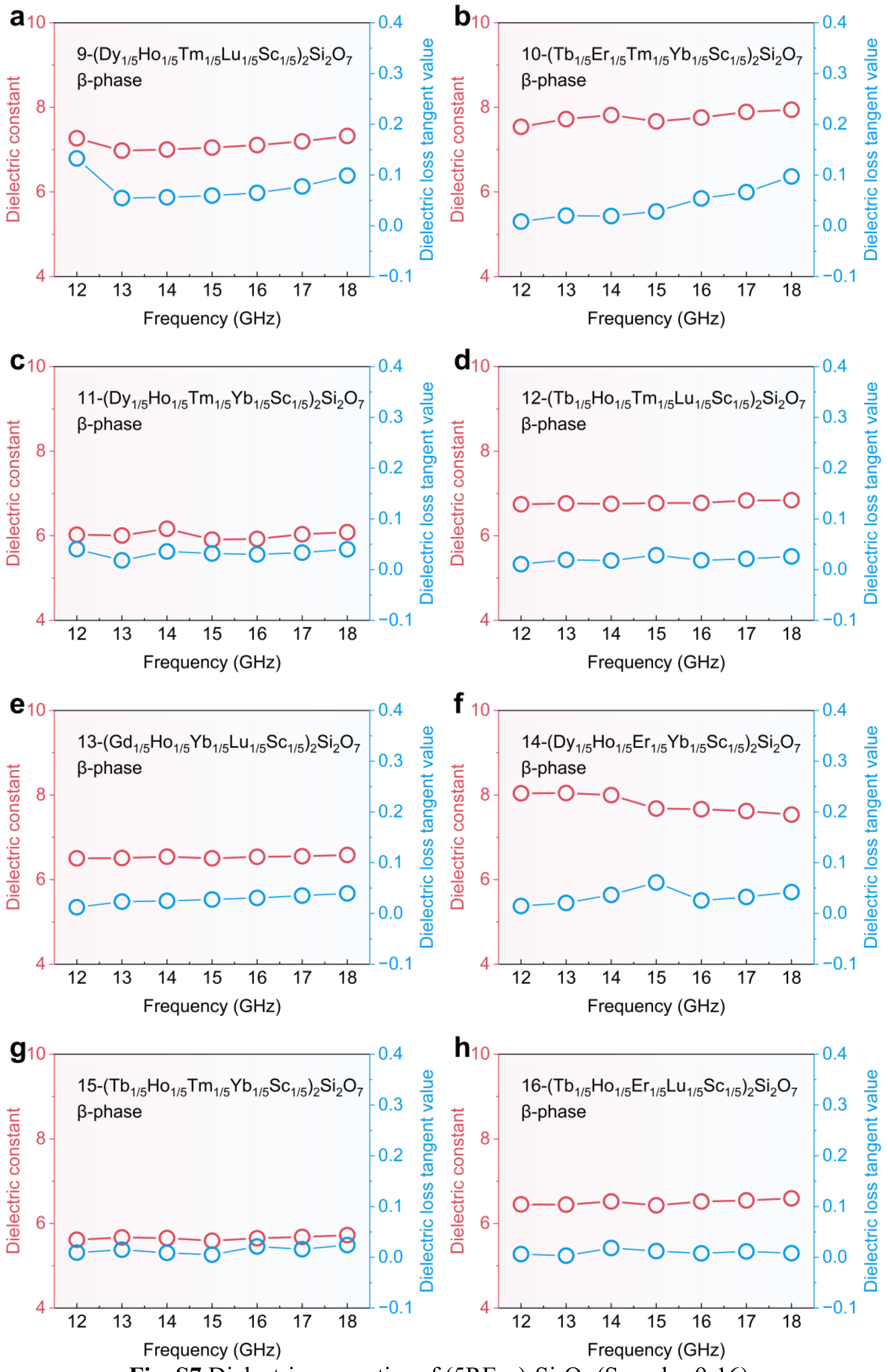

Fig. S7 Dielectric properties of $(5RE_{1/5})_2Si_2O_7$ (Samples 9-16).

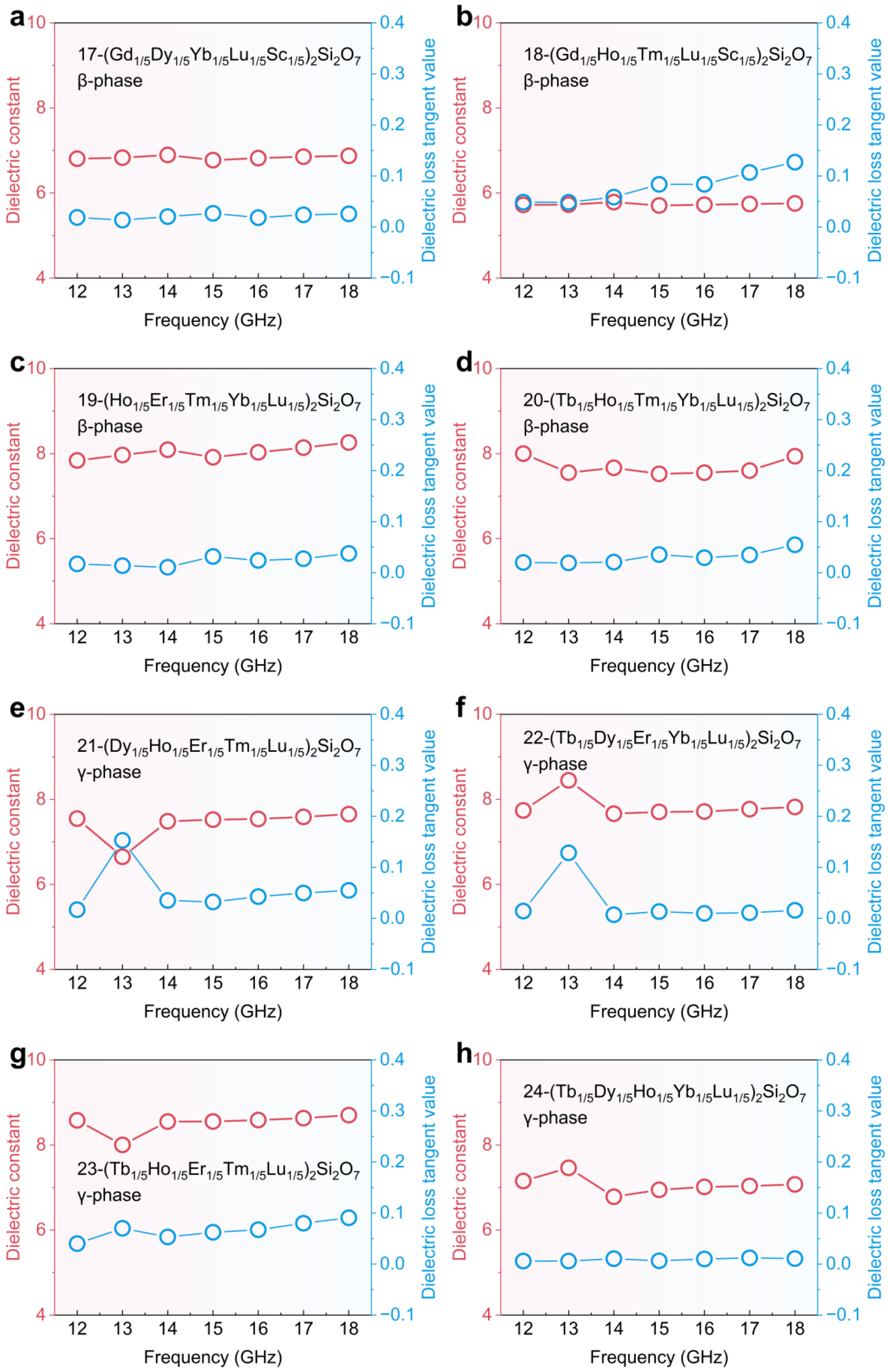

Fig. S8 Dielectric properties of $(5RE_{1/5})_2Si_2O_7$ (Samples 17-24).

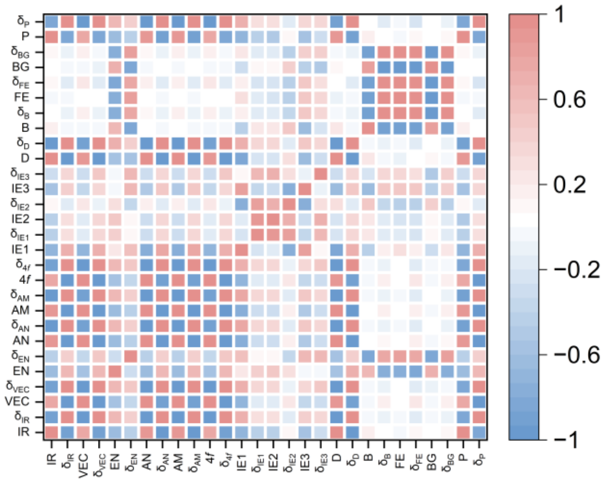

Fig. S9 Pearson correlation coefficient map of the initial 28 features from 24 bulk samples.

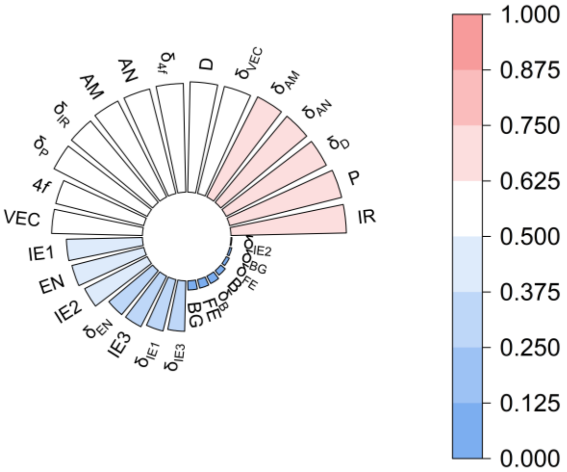

Fig. S10 The correlation analysis between descriptors and dielectric constants.

Table S5 Dielectric constants (ε) at 15 GHz of the as-synthesized 24 bulk samples, with the 6 descriptors. (Note: Sample abbreviations such as HoTmYbLuSc represent equimolar $(Ho_{1/5}Tm_{1/5}Yb_{1/5}Lu_{1/5}Sc_{1/5})_2Si_2O_7$.)

Index	Sample	IR (Å)	VEC	EN	δEN	IE1 (kJ/mol)	IE2 (kJ/mol)	ε
1	HoTmYbLuSc	0.8510	12.8	1.242	0.0496	587.54	1209.96	5.40
2	HoErYbLuSc	0.8530	12.6	1.240	0.0484	586.06	1207.96	5.00
3	DyErYbLuSc	0.8552	12.4	1.238	0.0504	584.46	1205.96	7.36
4	TbTmYbLuSc	0.8554	12.4	1.236	0.0557	584.50	1203.96	6.35
5	HoErTmLuSc	0.8554	12.4	1.270	0.0283	584.72	1205.00	6.10
6	DyHoYbLuSc	0.8574	12.2	1.236	0.0511	582.80	1203.96	6.05
7	DyErTmLuSc	0.8576	12.2	1.268	0.0297	583.12	1203.00	6.29
8	TbHoYbLuSc	0.8596	12.0	1.232	0.0539	581.36	1199.96	7.02
9	DyHoTmLuSc	0.8598	12.0	1.266	0.0310	581.46	1201.00	7.05
10	TbErTmYbSc	0.8612	11.8	1.230	0.0520	597.66	1165.96	7.67
11	DyHoTmYbSc	0.8612	11.8	1.232	0.0474	597.44	1167.96	5.91
12	TbHoTmLuSc	0.8620	11.8	1.262	0.0336	580.02	1197.00	6.77
13	GdHoYbLuSc	0.8626	11.8	1.232	0.0539	586.88	1211.36	6.50
14	DyHoErYbSc	0.8632	11.6	1.228	0.0455	595.96	1165.96	7.68
15	TbHoTmYbSc	0.8634	11.6	1.228	0.0508	596.00	1163.96	5.59
16	TbHoErLuSc	0.8640	11.6	1.260	0.0349	578.54	1195.00	6.43
17	GdDyYbLuSc	0.8648	11.6	1.230	0.0553	585.28	1209.36	6.77
18	GdHoTmLuSc	0.8650	11.6	1.262	0.0336	585.54	1208.4	5.70
19	HoErTmYbLu	0.8800	15.0	1.218	0.0388	578.78	1192.96	7.91
20	TbHoTmYbLu	0.8866	14.4	1.210	0.0397	574.08	1184.96	7.52
21	DyHoErTmLu	0.8886	14.2	1.208	0.0384	572.60	1182.96	7.52
22	TbDyErYbLu	0.8908	14.0	1.206	0.0371	571.00	1180.96	7.70
23	TbHoErTmLu	0.8910	14.0	1.238	0.0149	571.26	1180.00	8.55
24	TbDyHoYbLu	0.8930	13.8	1.204	0.0359	569.34	1178.96	6.95

Table S6 RE-O bond length of the as-synthesized 24 bulk samples from DFT calculations. (Note: Sample abbreviations such as HoTmYbLuSc represent equimolar $(Ho_{1/5}Tm_{1/5}Yb_{1/5}Lu_{1/5}Sc_{1/5})_2Si_2O_7$.)

**Index**	**Sample**	**Average bond length (Å)**
1	HoTmYbLuSc	2.225
2	HoErYbLuSc	2.228
3	DyErYbLuSc	2.230
4	TbTmYbLuSc	2.231
5	HoErTmLuSc	2.230
6	DyHoYbLuSc	2.233
7	DyErTmLuSc	2.233
8	TbHoYbLuSc	2.236
9	DyHoTmLuSc	2.235
10	TbErTmYbSc	2.238
11	DyHoTmYbSc	2.237
12	TbHoTmLuSc	2.238
13	GdHoYbLuSc	2.239
14	DyHoErYbSc	2.240
15	TbHoTmYbSc	2.240
16	TbHoErLuSc	2.241
17	GdDyYbLuSc	2.241
18	GdHoTmLuSc	2.241
19	HoErTmYbLu	2.248
20	TbHoTmYbLu	2.256
21	DyHoErTmLu	2.261
22	TbDyErYbLu	2.264
23	TbHoErTmLu	2.264
24	TbDyHoYbLu	2.266

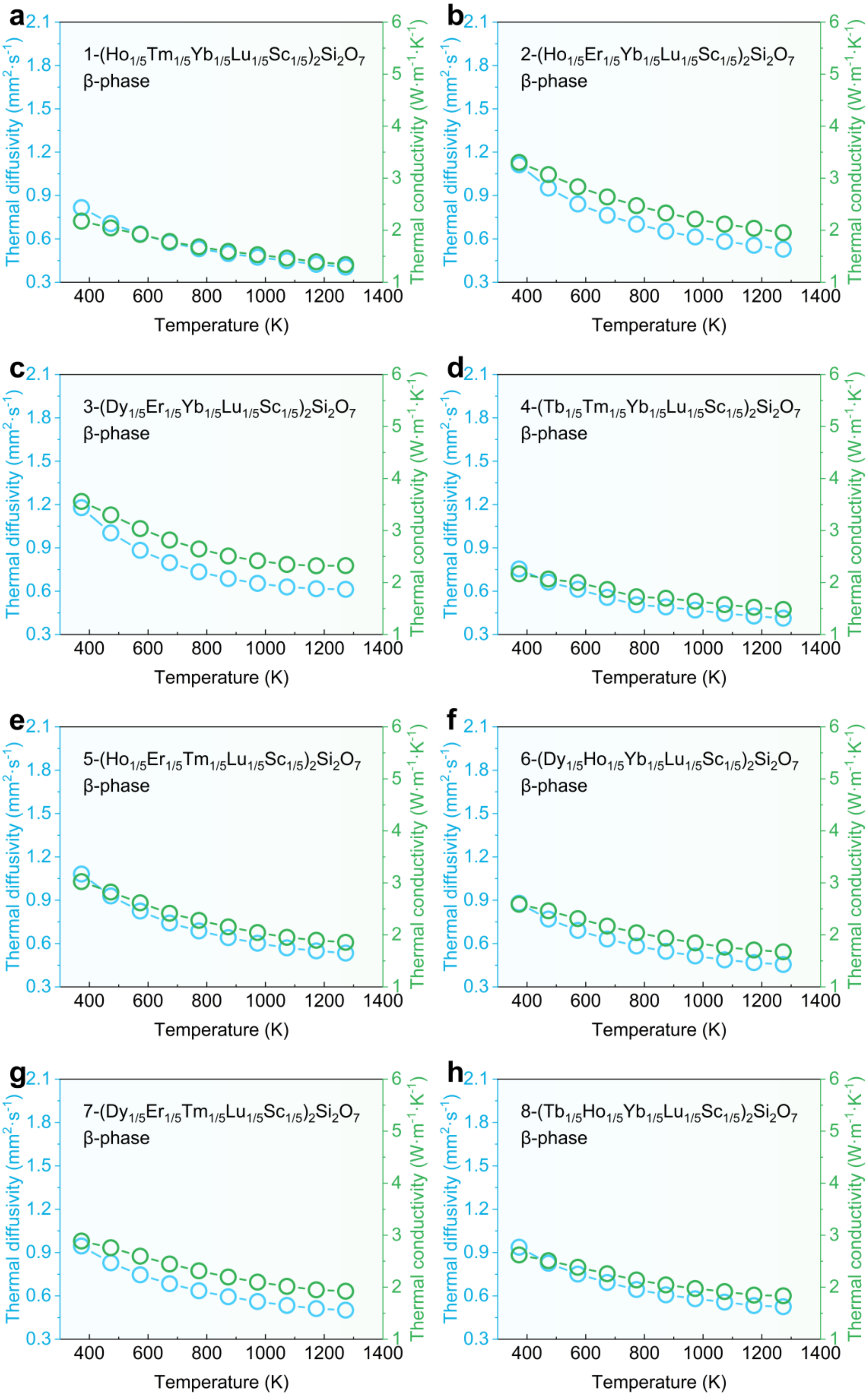

Fig. S11 Thermal diffusivities and conductivities of $(5RE_{1/5})_2Si_2O_7$ (Samples 1-8).

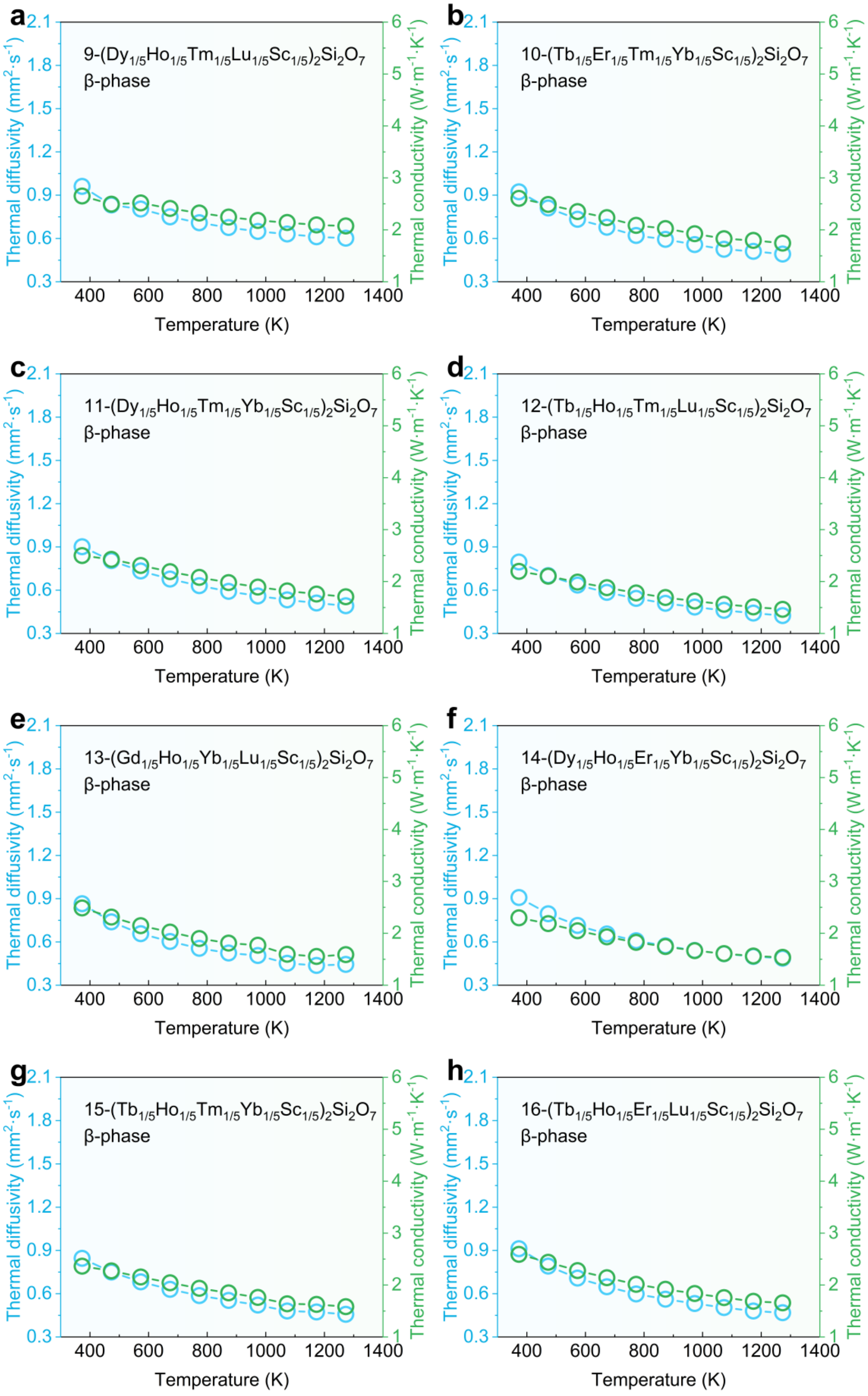

Fig. S12 Thermal diffusivities and conductivities of $(5RE_{1/5})_2Si_2O_7$ (Samples 9-16).

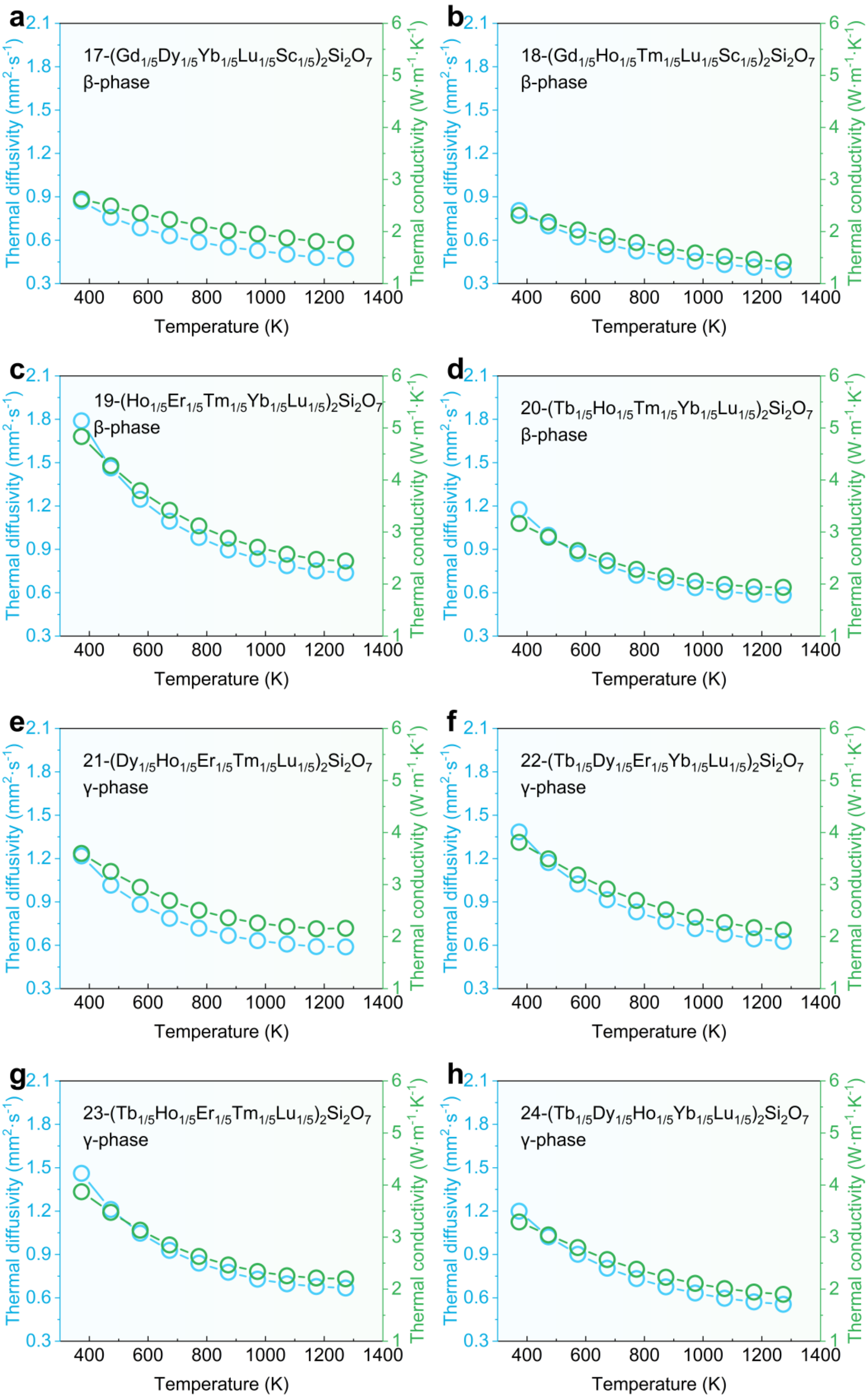

Fig. S13 Thermal diffusivities and conductivities of $(5RE_{1/5})_2Si_2O_7$ (Samples 17-24).

Table S7 Parameters of the Clarke model.

Parameter	*M* (g/mol)	*n*	*ρ* (g/cm^3)	*E* (GPa)
Value	458.9	11	5.46	175

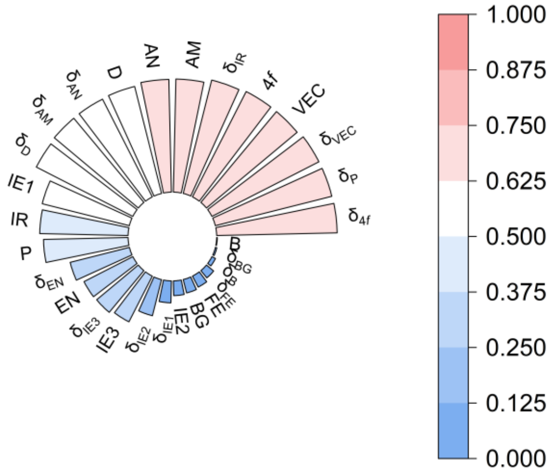

Fig. S14 The correlation analysis between descriptors and thermal conductivities.

Table S8 Thermal conductivities (κ) at 1273 K of the as-synthesized 24 bulk samples, with the 5 descriptors. (Note: Sample abbreviations such as HoTmYbLuSc represent equimolar $(Ho_{1/5}Tm_{1/5}Yb_{1/5}Lu_{1/5}Sc_{1/5})_2Si_2O_7$.)

Index	Sample	δIR	EN	δEN	IE1 (kJ/mol)	δIE3	κ (W· m^{-1}· K^{-1})
1	HoTmYbLuSc	5.471	1.242	0.0496	587.54	0.0531	1.34
2	HoErYbLuSc	5.590	1.240	0.0484	586.06	0.0562	1.95
3	DyErYbLuSc	5.793	1.238	0.0504	584.46	0.0565	2.33
4	TbTmYbLuSc	5.925	1.236	0.0557	584.50	0.0631	1.48
5	HoErTmLuSc	5.675	1.270	0.0283	584.72	0.0426	1.86
6	DyHoYbLuSc	5.940	1.236	0.0511	582.80	0.0557	1.67
7	DyErTmLuSc	5.865	1.268	0.0297	583.12	0.0429	1.92
8	TbHoYbLuSc	6.155	1.232	0.0539	581.36	0.0623	1.83
9	DyHoTmLuSc	6.002	1.266	0.0310	581.46	0.0421	2.07
10	TbErTmYbSc	6.091	1.230	0.0520	597.66	0.0441	1.74
11	DyHoTmYbSc	6.011	1.232	0.0474	597.44	0.0361	1.71
12	TbHoTmLuSc	6.206	1.262	0.0336	580.02	0.0489	1.46
13	GdHoYbLuSc	6.484	1.232	0.0539	586.88	0.0720	1.59
14	DyHoErYbSc	6.082	1.228	0.0455	595.96	0.0428	1.54
15	TbHoTmYbSc	6.210	1.228	0.0508	596.00	0.0430	1.58
16	TbHoErLuSc	6.276	1.260	0.0349	578.54	0.0426	1.68
17	GdDyYbLuSc	6.628	1.230	0.0553	585.28	0.0723	1.78
18	GdHoTmLuSc	6.522	1.262	0.0336	585.54	0.0631	1.42
19	HoErTmYbLu	1.446	1.218	0.0388	578.78	0.0455	2.44
20	TbHoTmYbLu	2.270	1.210	0.0397	574.08	0.0516	1.94
21	DyHoErTmLu	2.247	1.208	0.0384	572.60	0.0446	2.16
22	TbDyErYbLu	2.406	1.206	0.0371	571.00	0.0443	2.13
23	TbHoErTmLu	2.072	1.238	0.0149	571.26	0.0354	2.20
24	TbDyHoYbLu	2.439	1.204	0.0359	569.34	0.0450	1.90

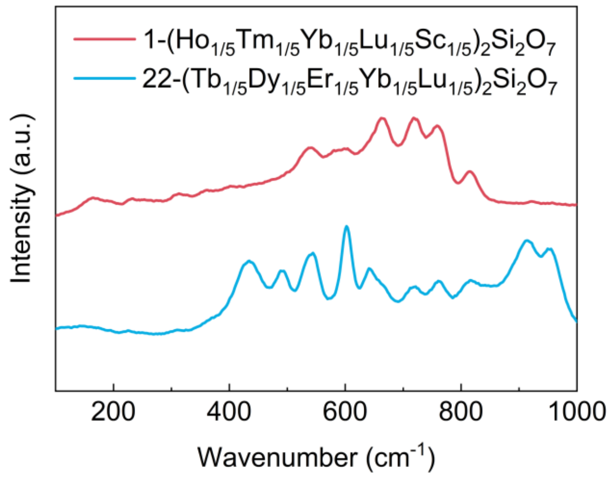

Fig. S15 Raman spectra of Sc-doped and Sc-free compositions.

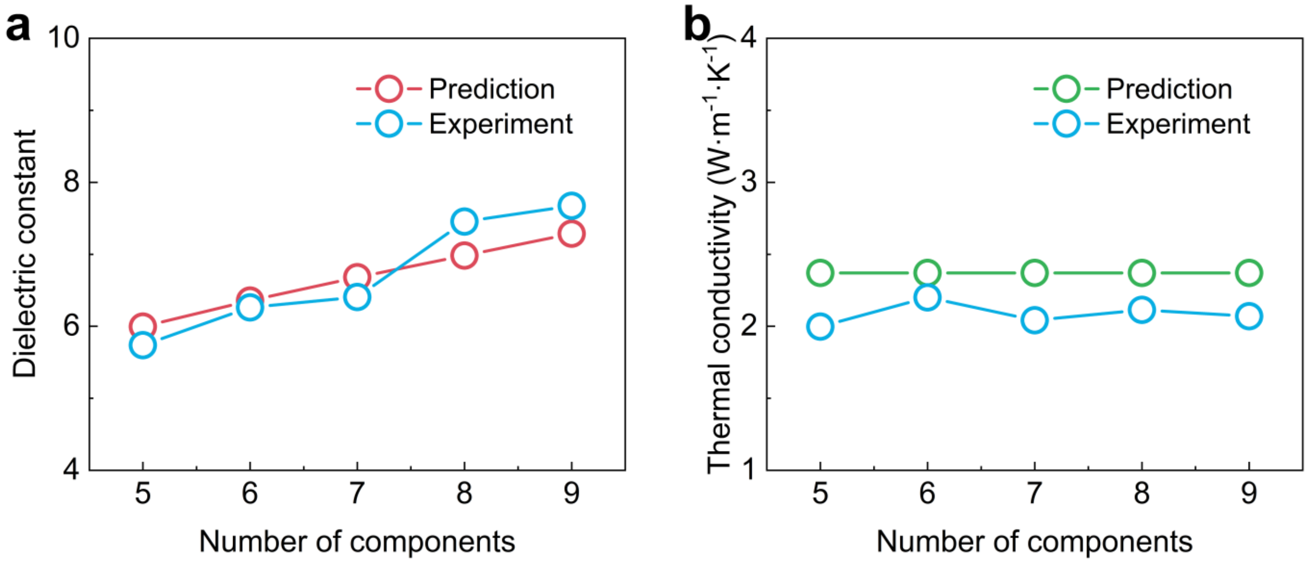

Fig. S16 Comparison of predicted and experimental values for (a) dielectric constant and (b) thermal conductivity in $(Er_{1/5}Tm_{1/5}Yb_{1/5}Lu_{1/5}Sc_{1/5})_2Si_2O_7$, $(Ho_{1/6}Er_{1/6}Tm_{1/6}Yb_{1/6}Lu_{1/6}Sc_{1/6})_2Si_2O_7$, $(Dy_{1/7}Ho_{1/7}Er_{1/7}Tm_{1/7}Yb_{1/7}Lu_{1/7}Sc_{1/7})_2Si_2O_7$, $(Tb_{1/8}Dy_{1/8}Ho_{1/8}Er_{1/8}Tm_{1/8}Yb_{1/8}Lu_{1/8}Sc_{1/8})_2Si_2O_7$, and $(Gd_{1/9}Tb_{1/9}Dy_{1/9}Ho_{1/9}Er_{1/9}Tm_{1/9}Yb_{1/9}Lu_{1/9}Sc_{1/9})_2Si_2O_7$.

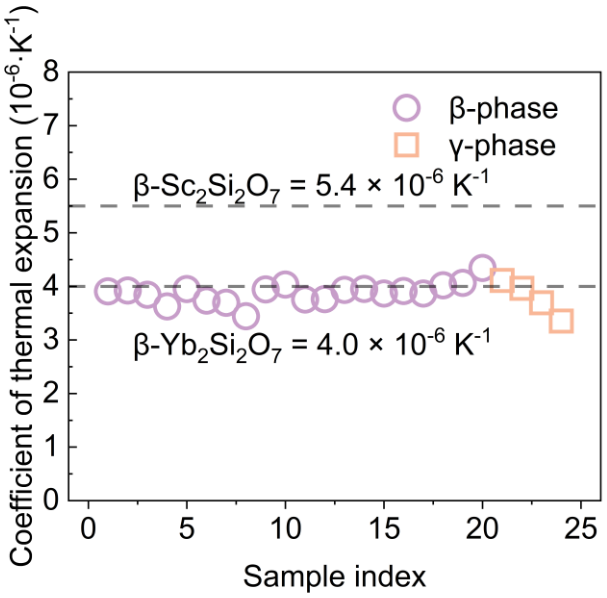

Fig. S17 Coefficients of thermal expansion of $(5RE_{1/5})_2Si_2O_7$.

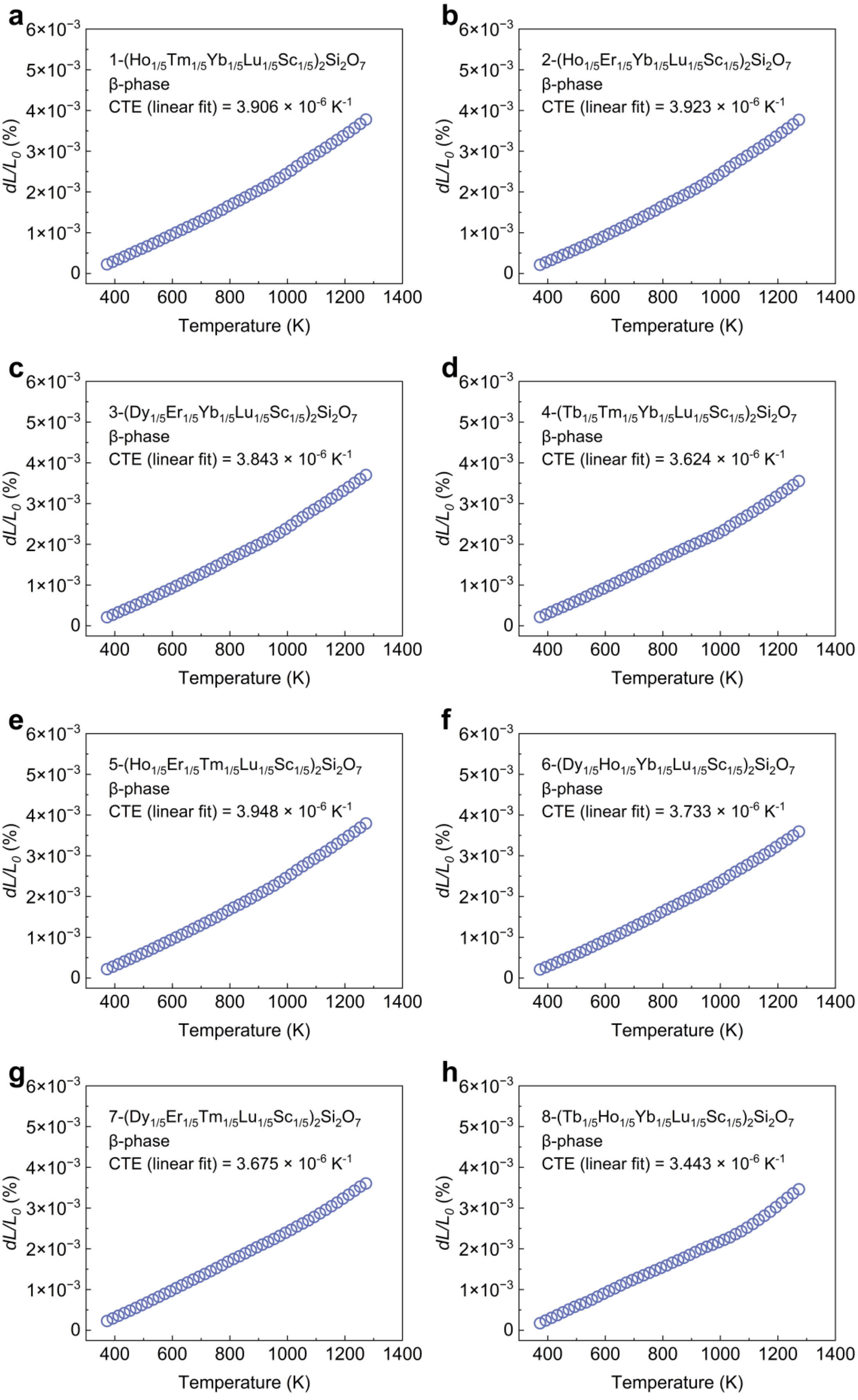

Fig. S18 Linear expansion rates of $(5RE_{1/5})_2Si_2O_7$ (Samples 1-8).

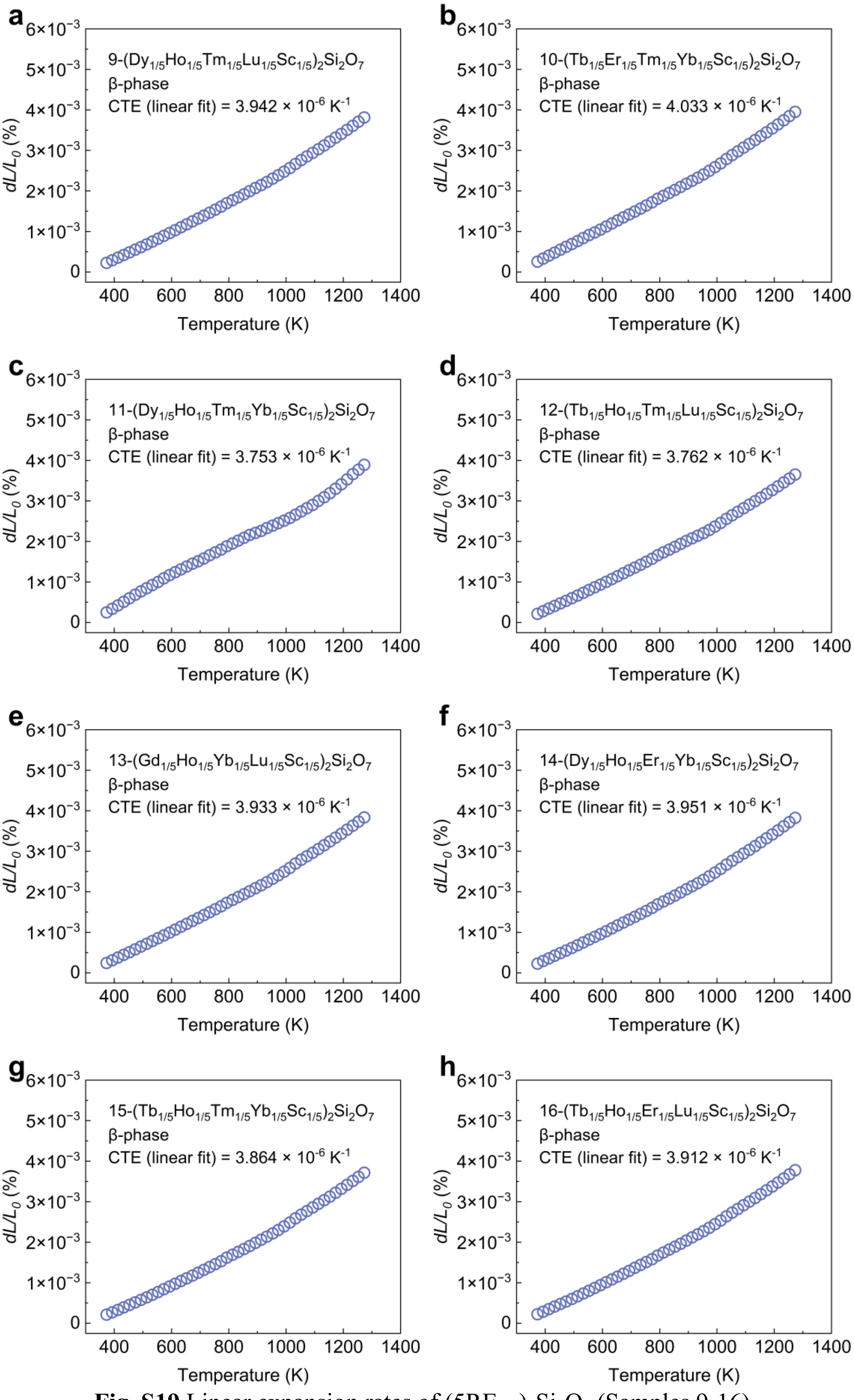

Fig. S19 Linear expansion rates of $(5RE_{1/5})_2Si_2O_7$ (Samples 9-16).

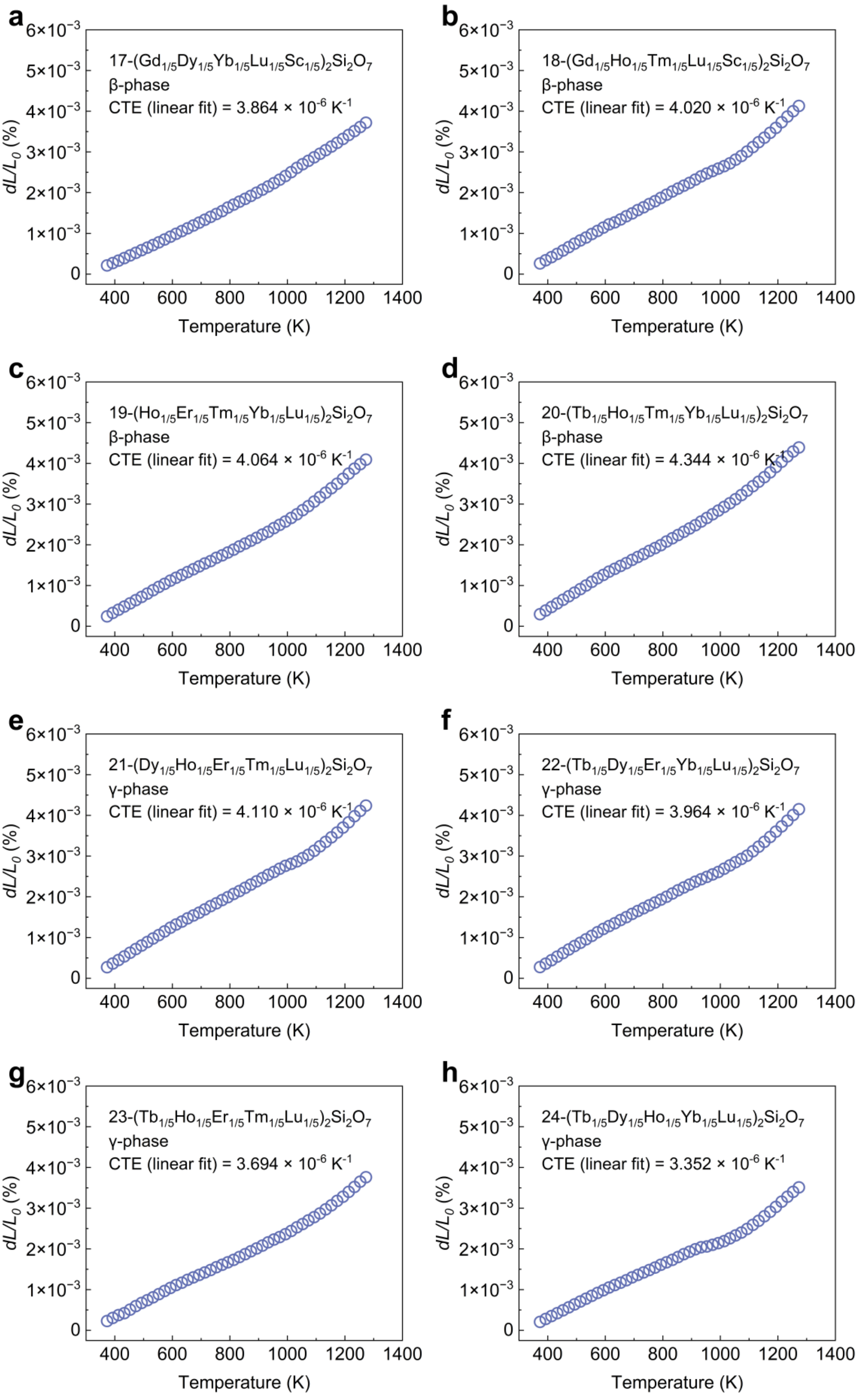

Fig. S20 Linear expansion rates of $(5RE_{1/5})_2Si_2O_7$ (Samples 17-24).

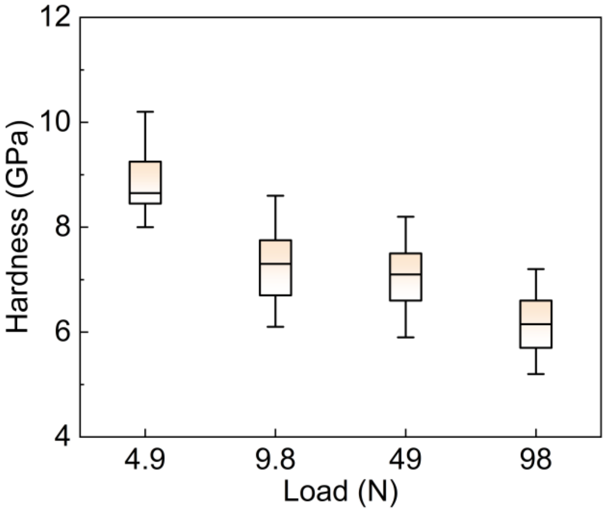

Fig. S21 Hardness of $(5RE_{1/5})_2Si_2O_7$.

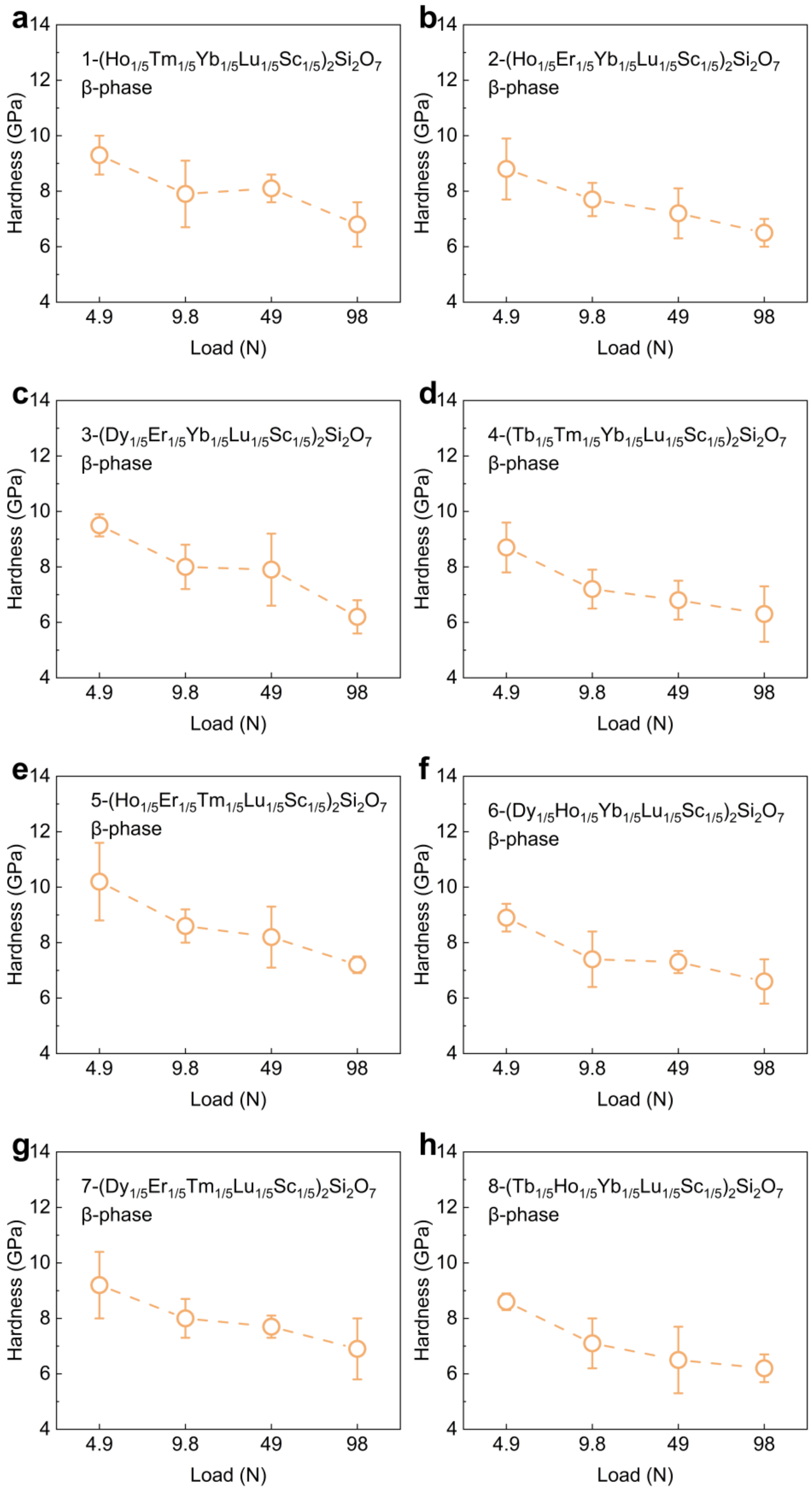

Fig. S22 Hardness of $(5RE_{1/5})_2Si_2O_7$ (Samples 1-8).

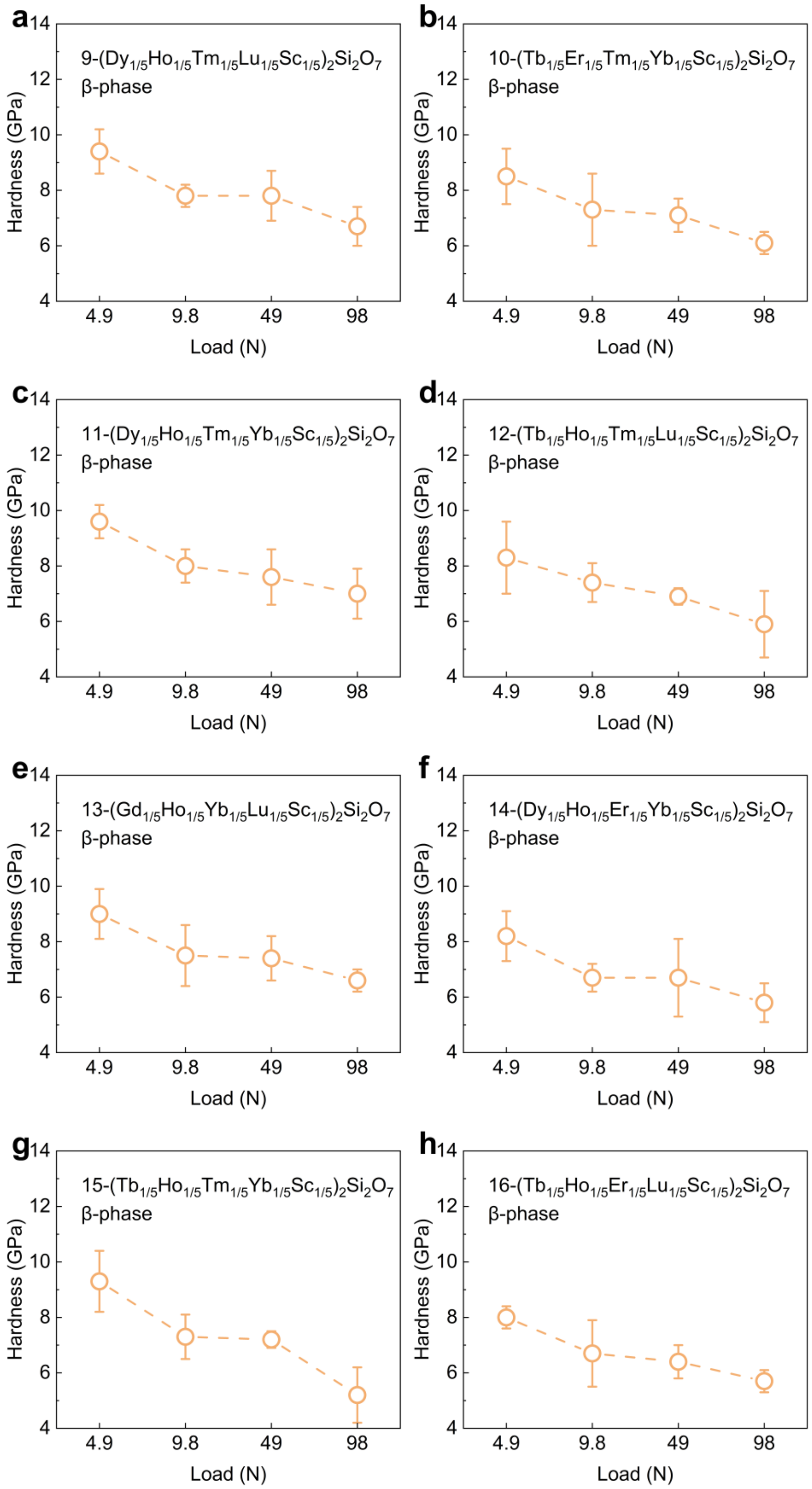

Fig. S23 Hardness of $(5RE_{1/5})_2Si_2O_7$ (Samples 9-16).

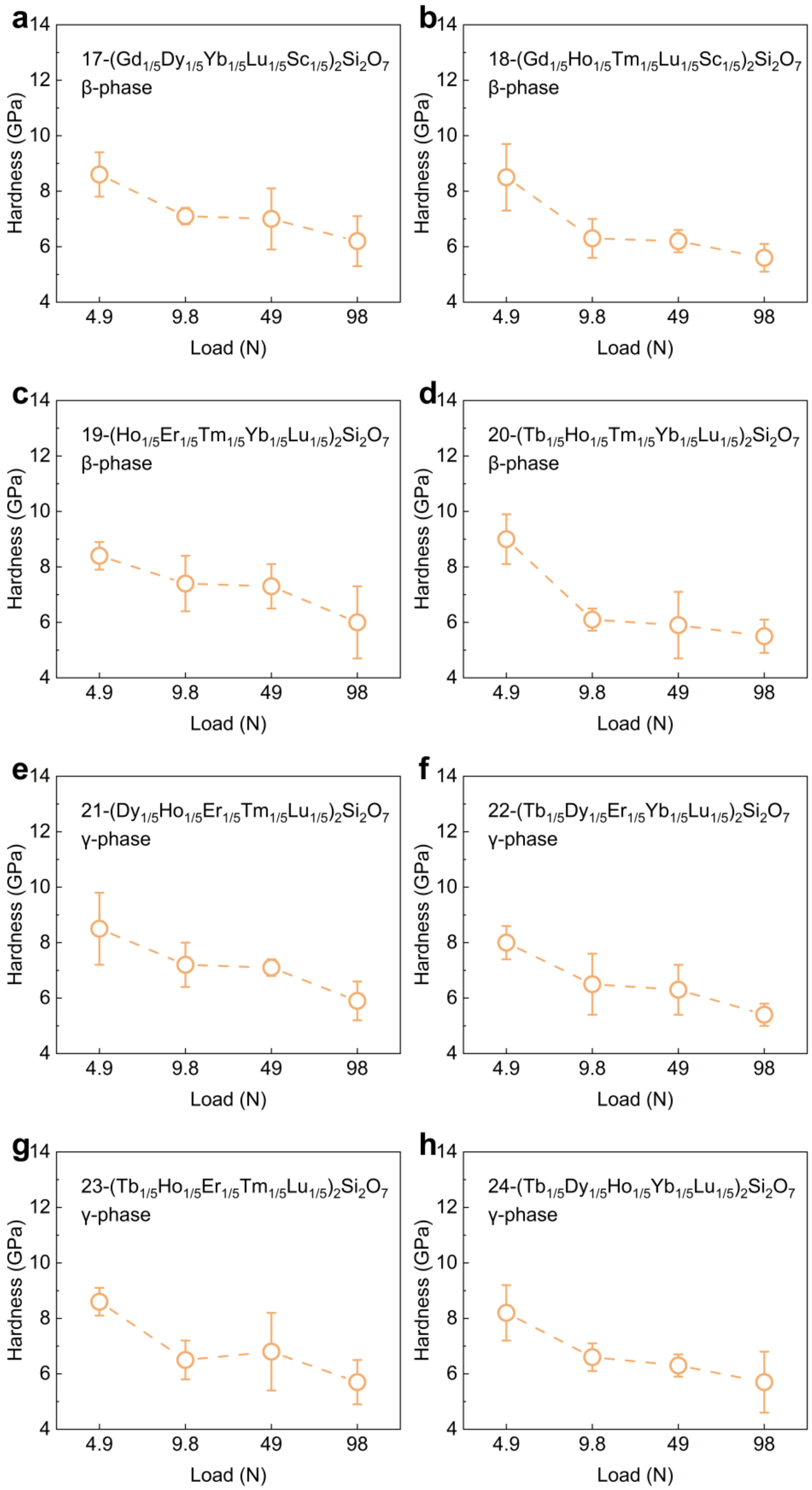

Fig. S24 Hardness of $(5RE_{1/5})_2Si_2O_7$ (Samples 17-24).

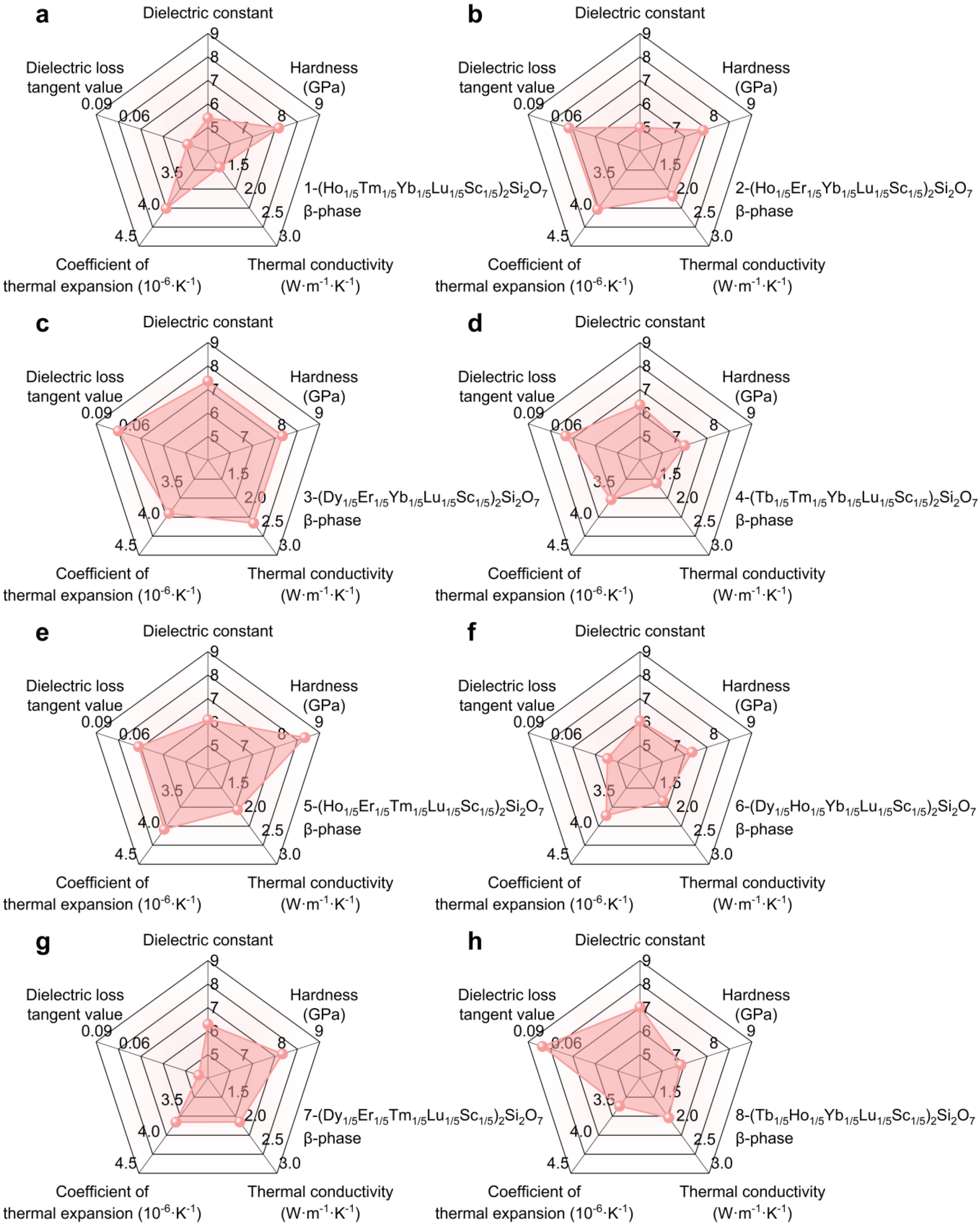

Fig. S25 Radar charts of $(5RE_{1/5})_2Si_2O_7$ (Samples 1-8).

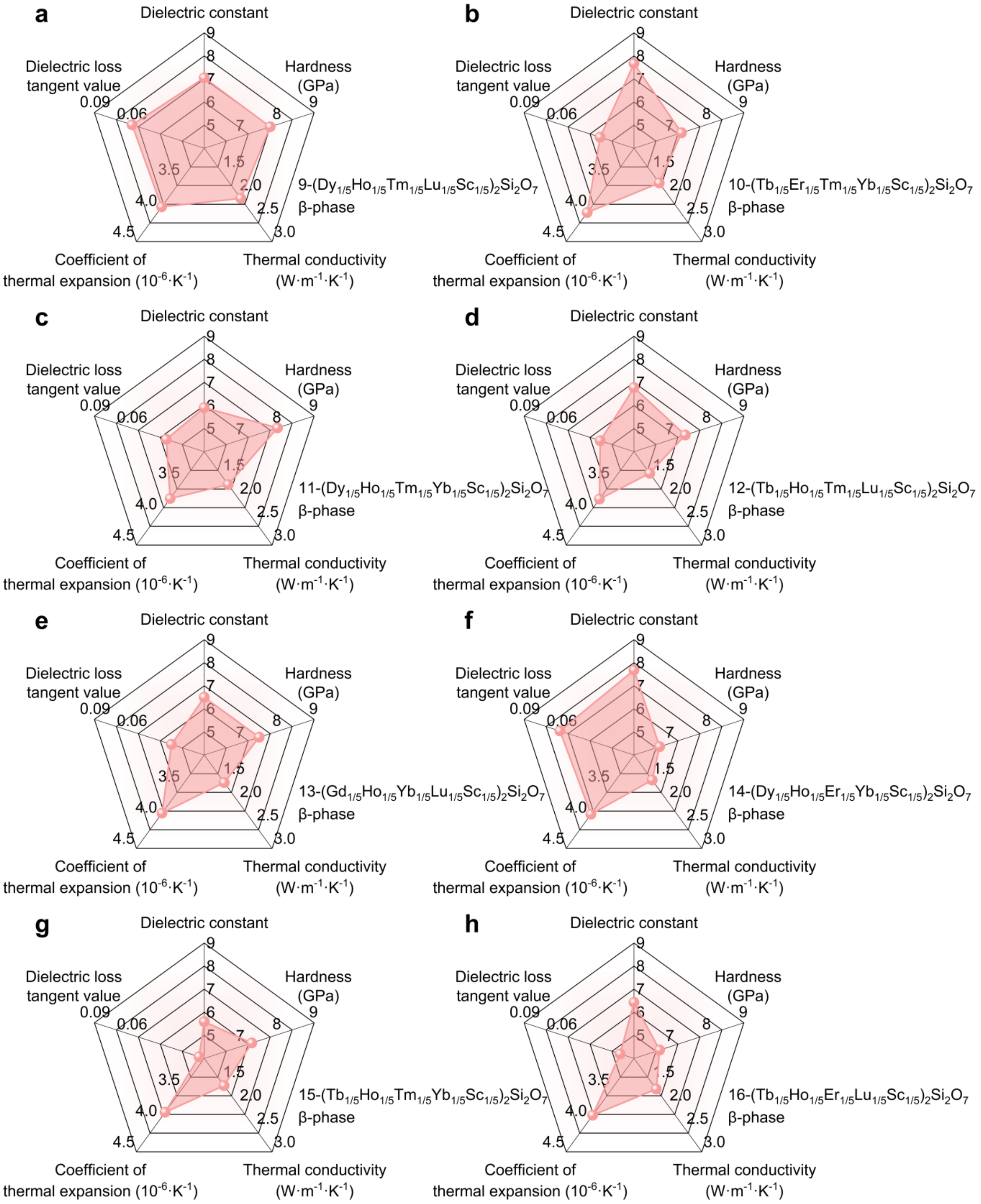

Fig. S26 Radar charts of $(5RE_{1/5})_2Si_2O_7$ (Samples 9-16).

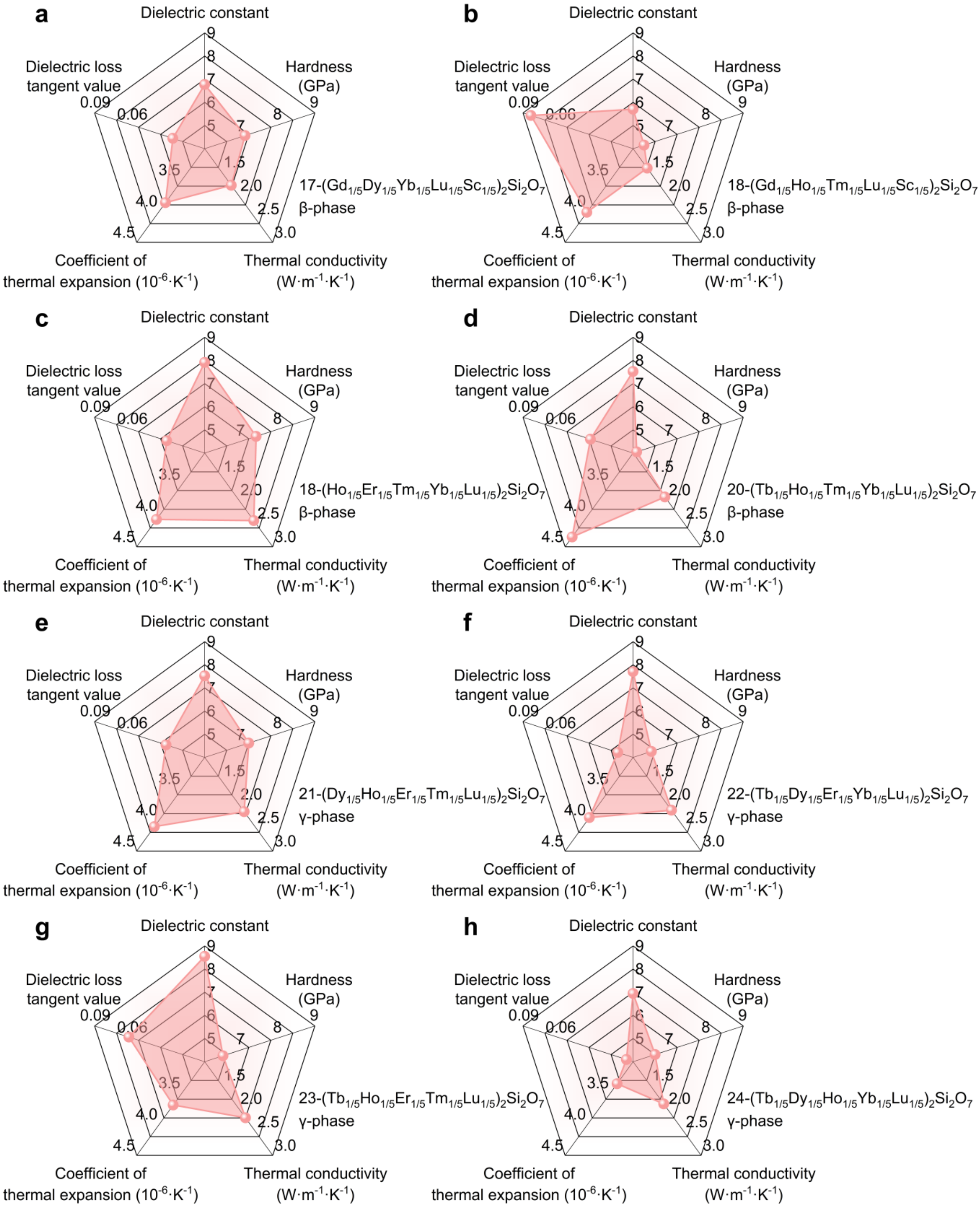

Fig. S27 Radar charts of $(5RE_{1/5})_2Si_2O_7$ (Samples 17-24).

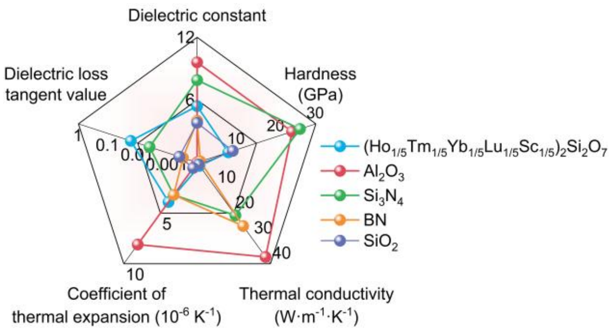

Fig. S28 Radar chart of $(Ho_{1/5}Tm_{1/5}Yb_{1/5}Lu_{1/5}Sc_{1/5})_2Si_2O_7$ and mainstream wave-transparent ceramics (e.g., Al_2O_3, Si_3N_4, BN, and SiO_2).
