# Supplementary figures and images for: Data-Driven Discovery of Composition–Structure–Property Relationship in Novel Wave-Transparent High-Entropy Rare Earth Disilicate
Source: Research (Wash D C). 2026 Jun 1;9:1308. doi: 10.34133/research.1308 (PMC13224110; doi:10.34133/research.1308)

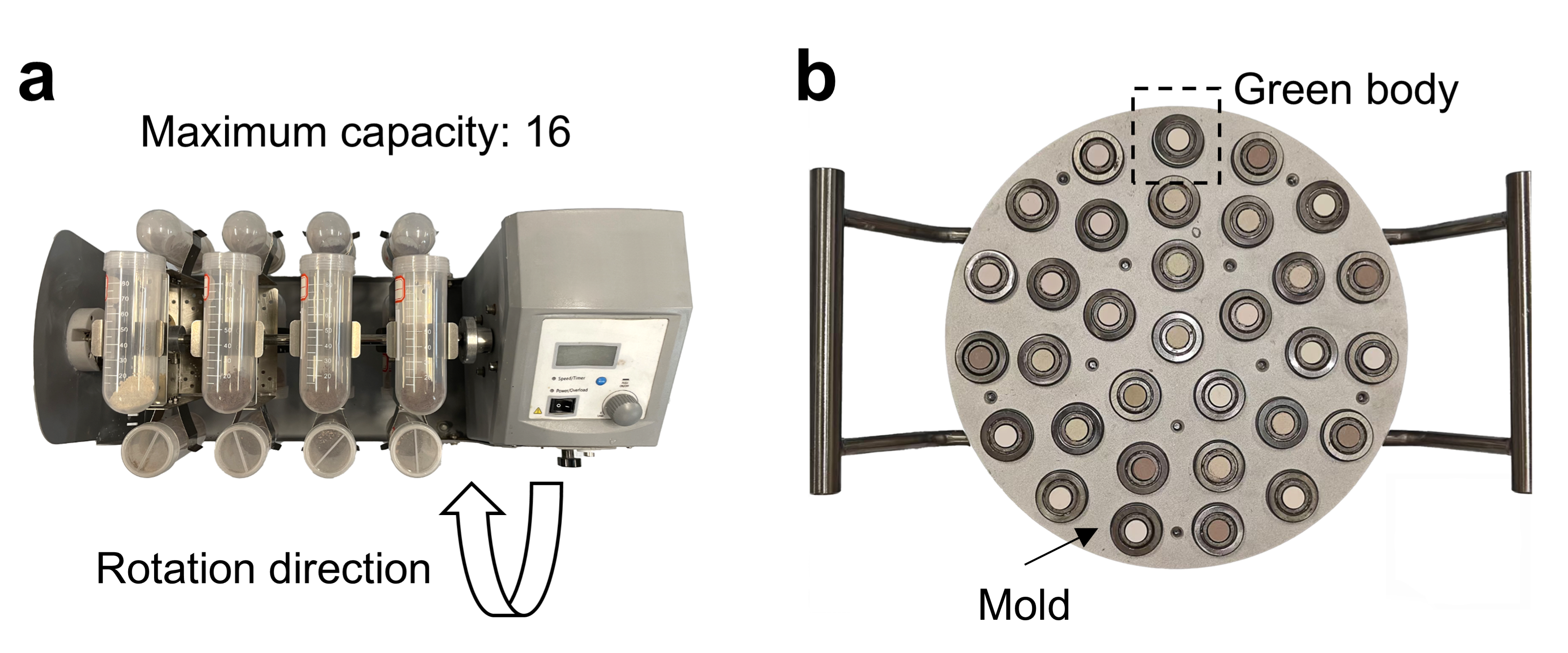

Supplement: Supplementary 1 — Figs. S1 to S28 Tables S1 to S8 [file research.1308.f1.zip › Fig_S1.tif]

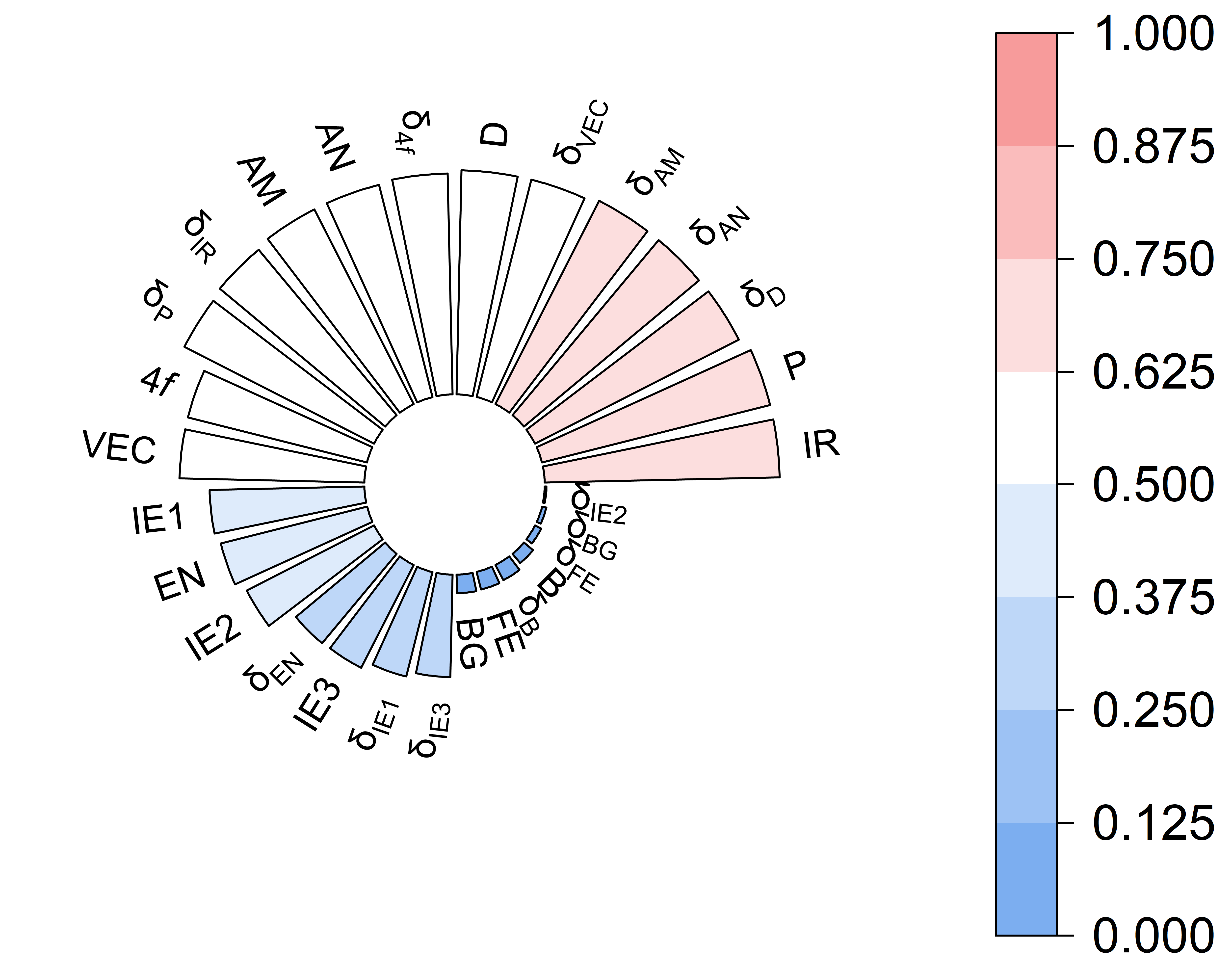

Supplement: Supplementary 1 — Figs. S1 to S28 Tables S1 to S8 [file research.1308.f1.zip › Fig_S10.tif]

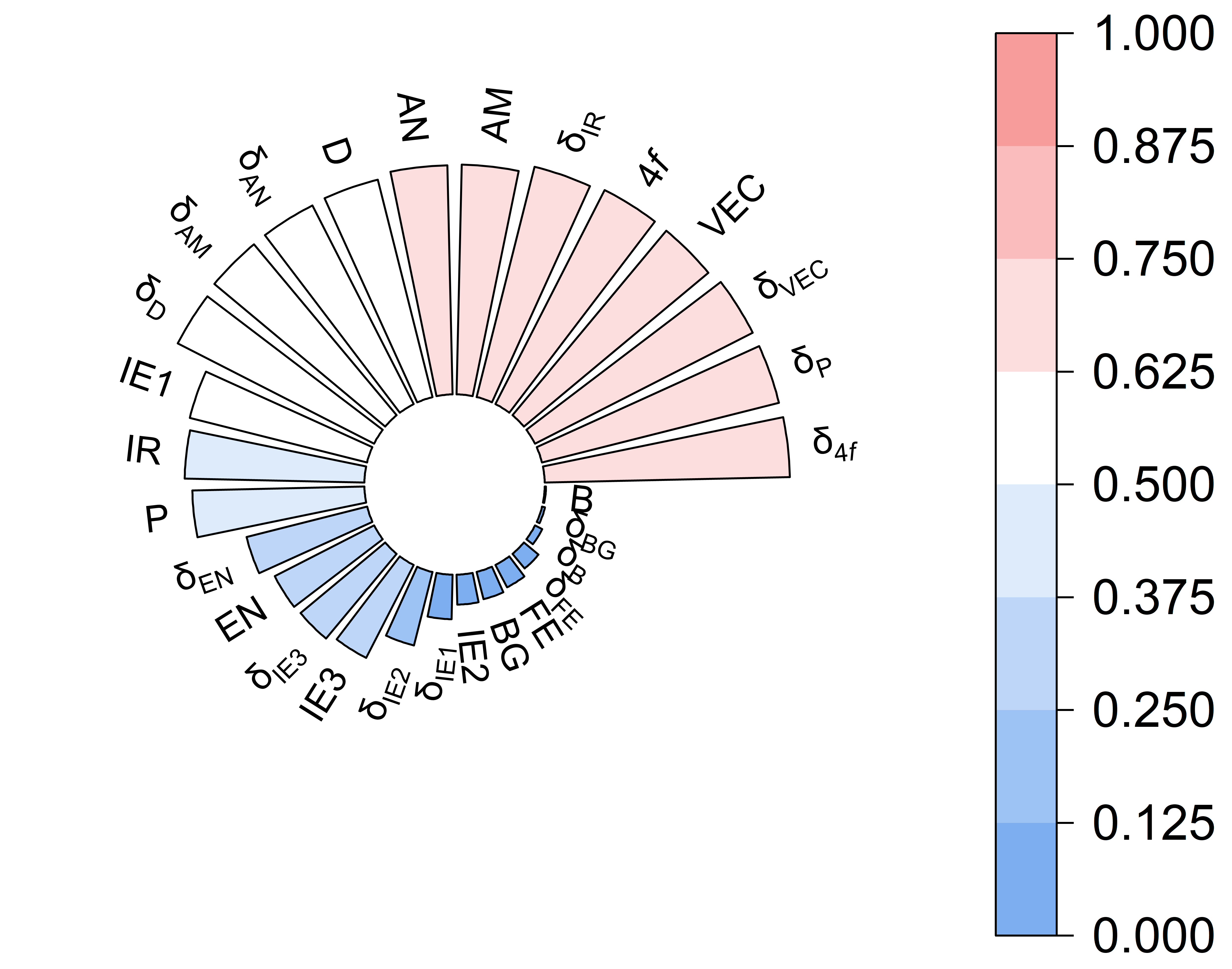

Supplement: Supplementary 1 — Figs. S1 to S28 Tables S1 to S8 [file research.1308.f1.zip › Fig_S14.tif]

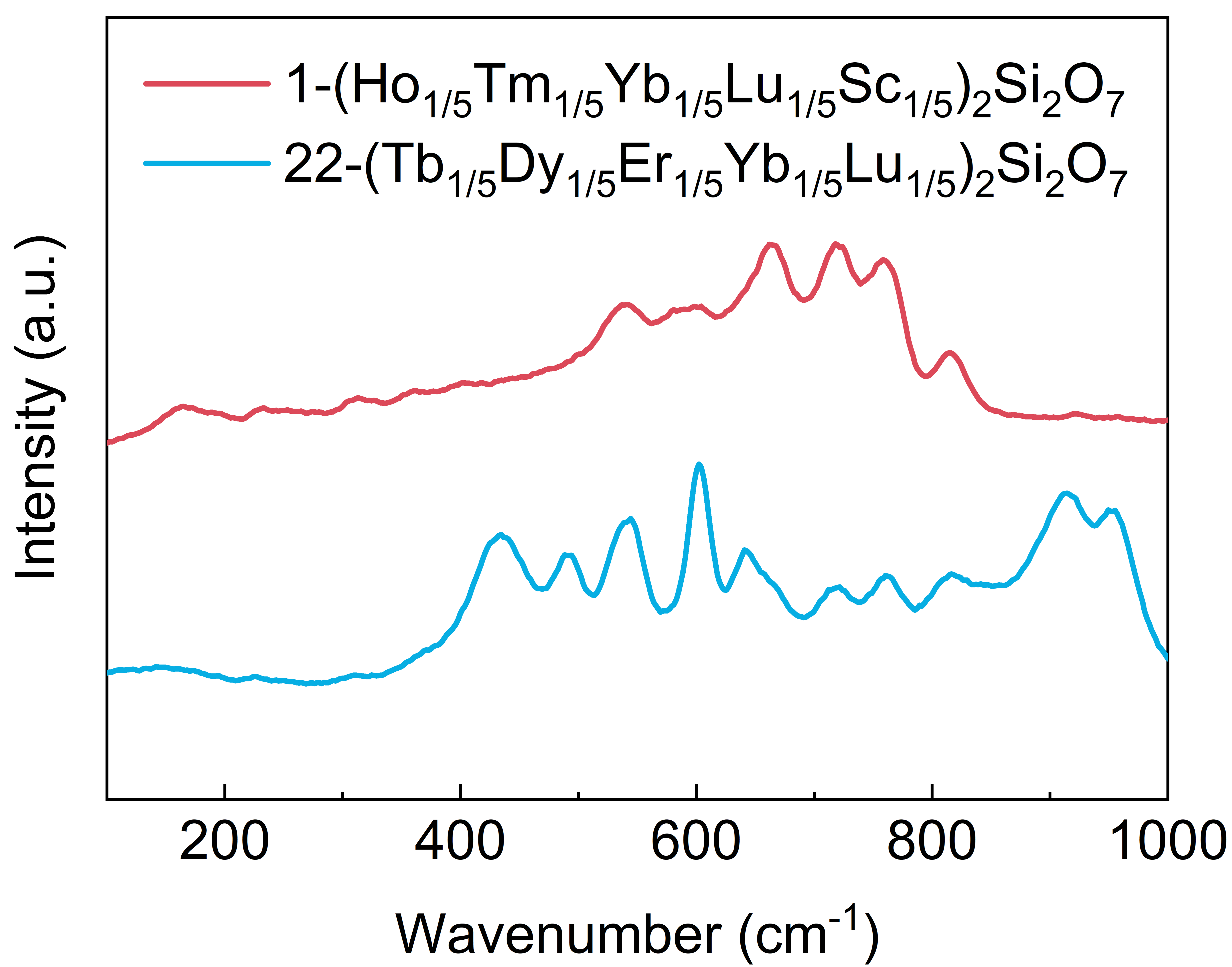

Supplement: Supplementary 1 — Figs. S1 to S28 Tables S1 to S8 [file research.1308.f1.zip › Fig_S15.tif]

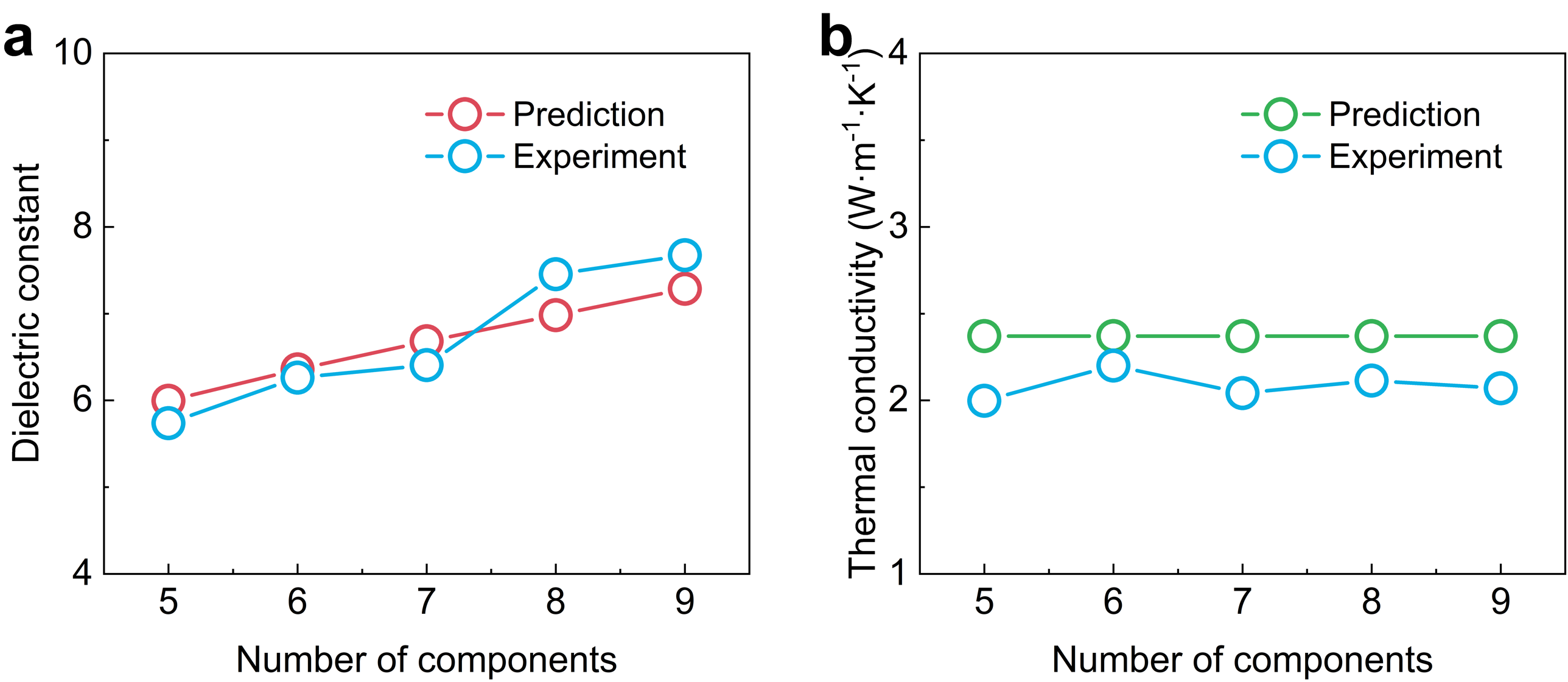

Supplement: Supplementary 1 — Figs. S1 to S28 Tables S1 to S8 [file research.1308.f1.zip › Fig_S16.tif]

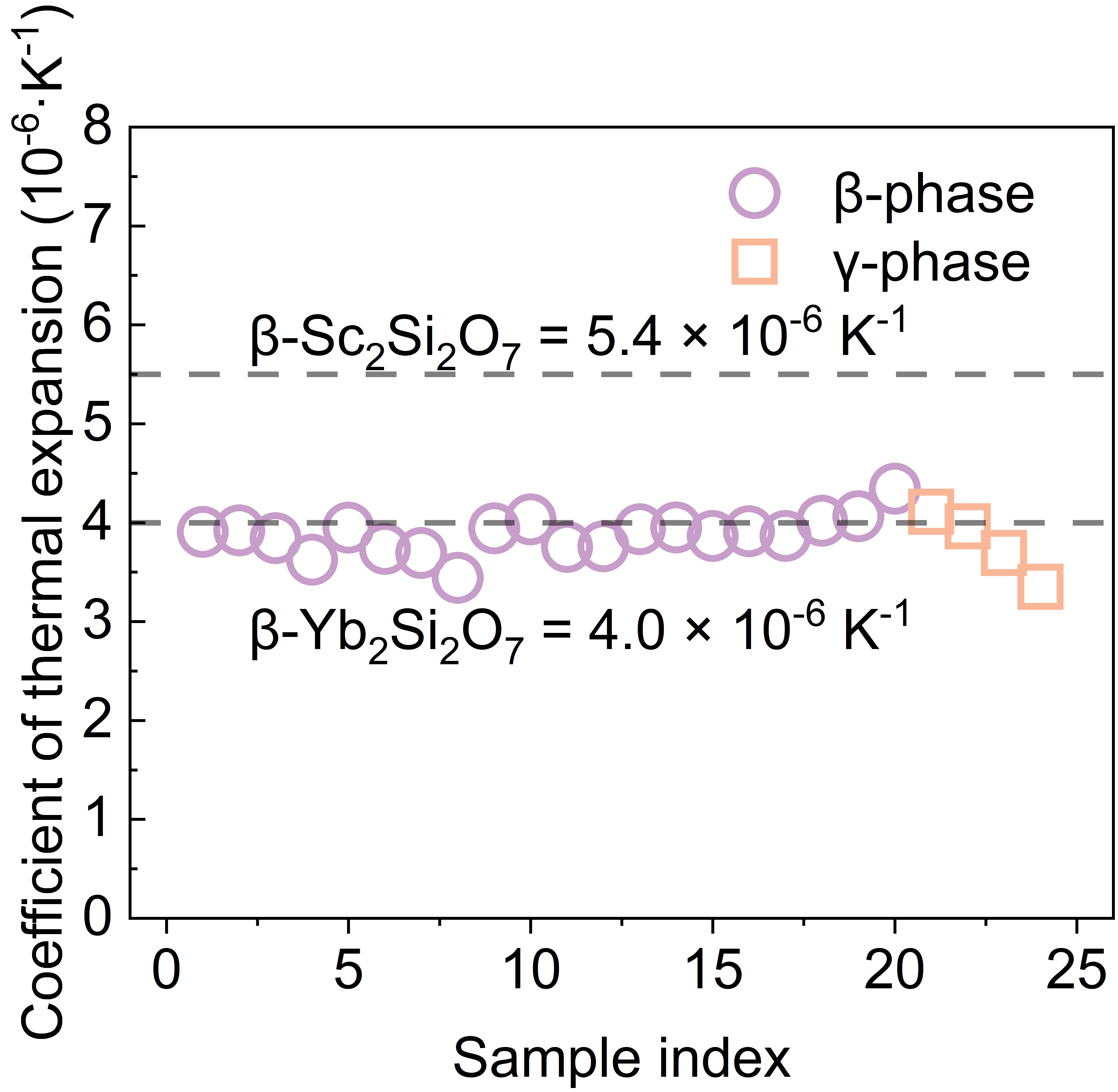

Supplement: Supplementary 1 — Figs. S1 to S28 Tables S1 to S8 [file research.1308.f1.zip › Fig_S17.tif]

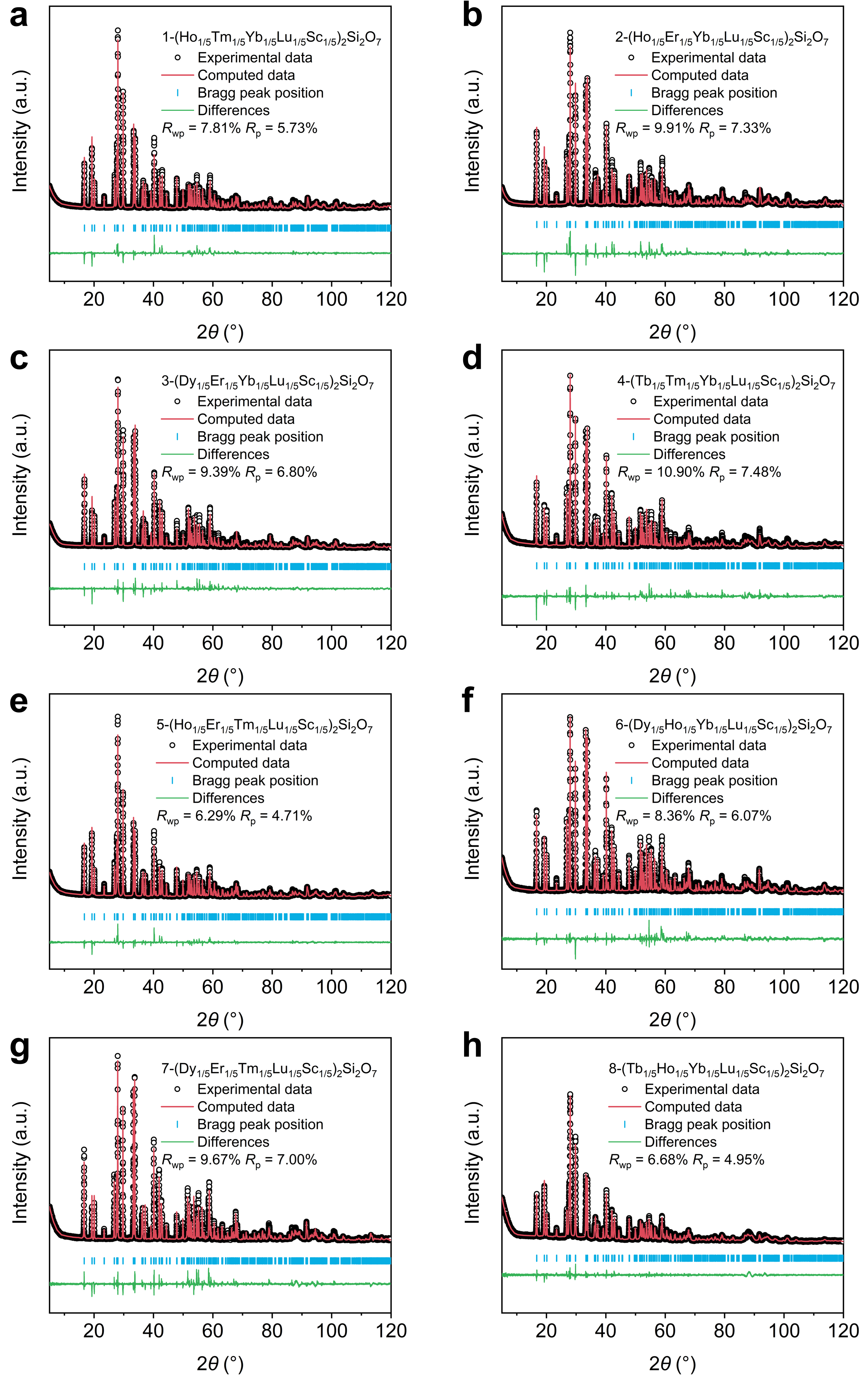

Supplement: Supplementary 1 — Figs. S1 to S28 Tables S1 to S8 [file research.1308.f1.zip › Fig_S2.tif]

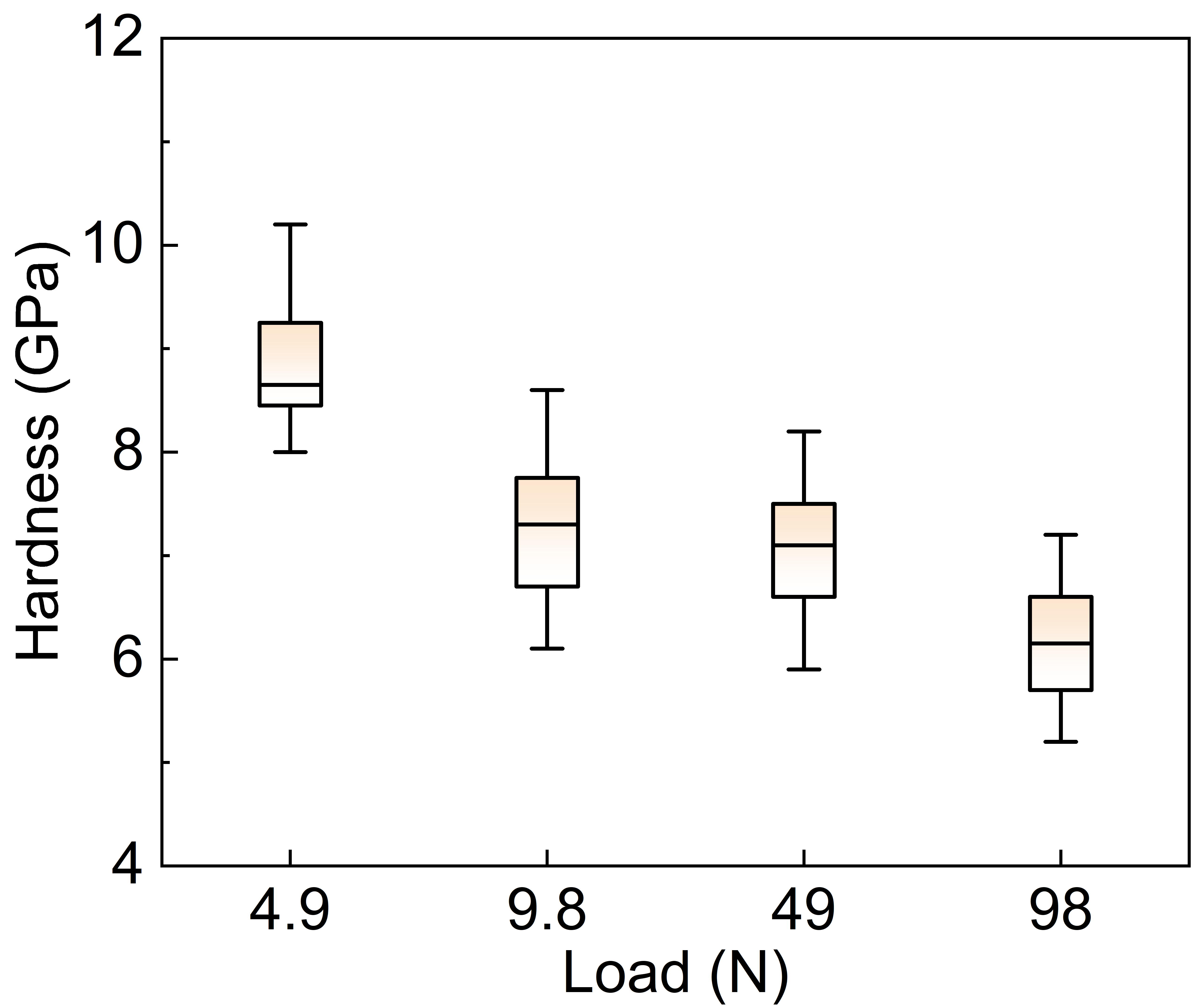

Supplement: Supplementary 1 — Figs. S1 to S28 Tables S1 to S8 [file research.1308.f1.zip › Fig_S21.tif]

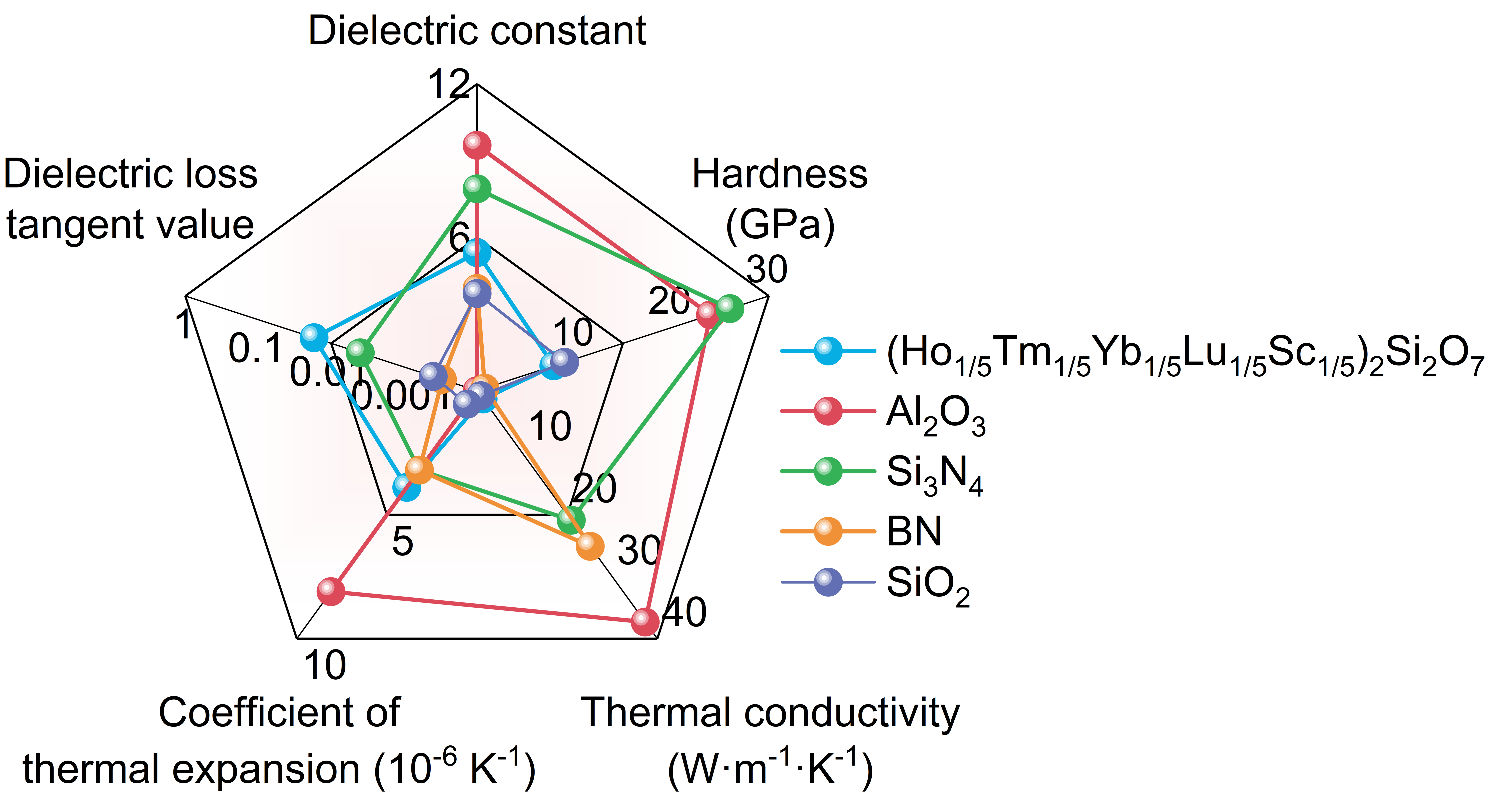

Supplement: Supplementary 1 — Figs. S1 to S28 Tables S1 to S8 [file research.1308.f1.zip › Fig_S28.tif]

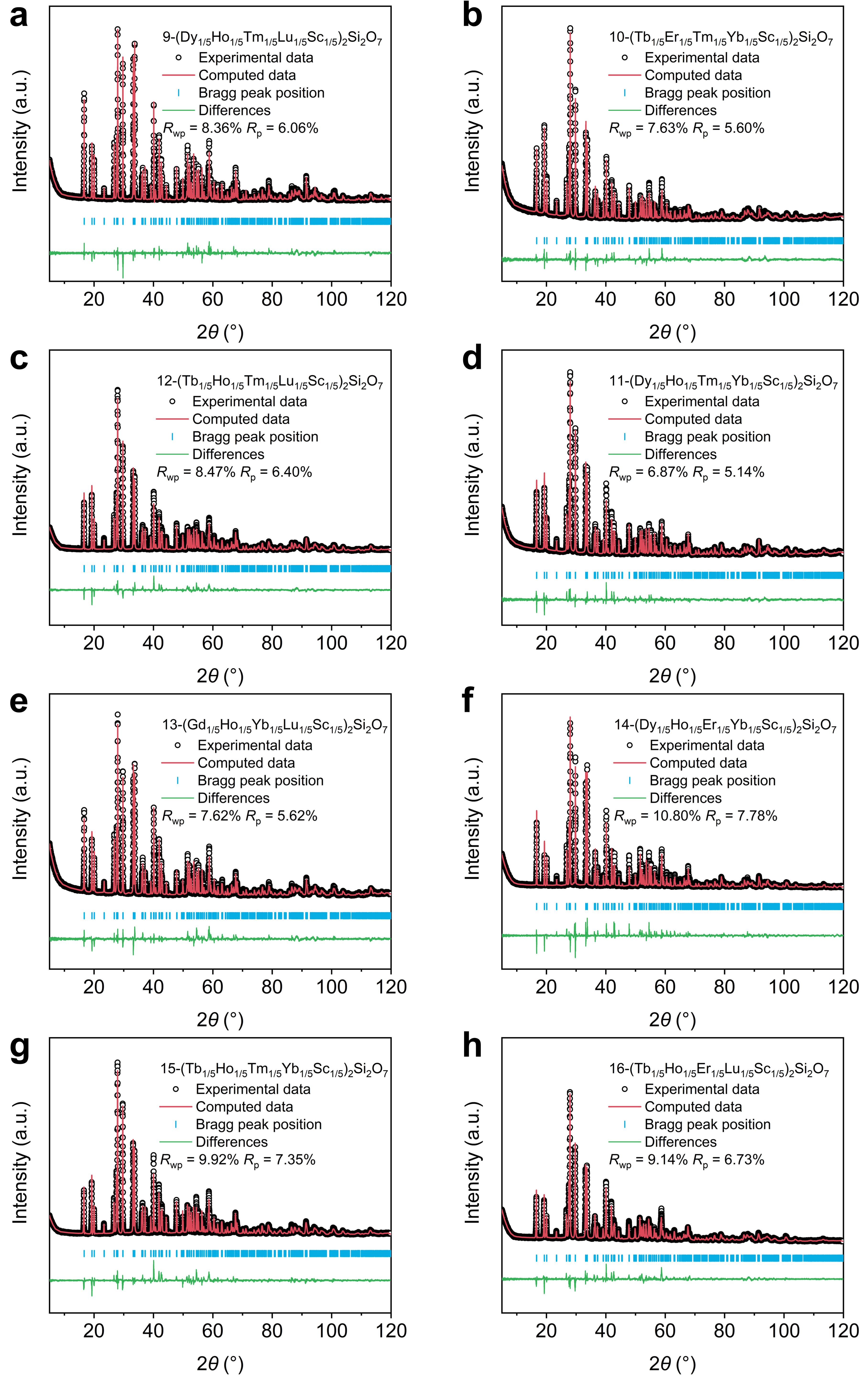

Supplement: Supplementary 1 — Figs. S1 to S28 Tables S1 to S8 [file research.1308.f1.zip › Fig_S3.tif]

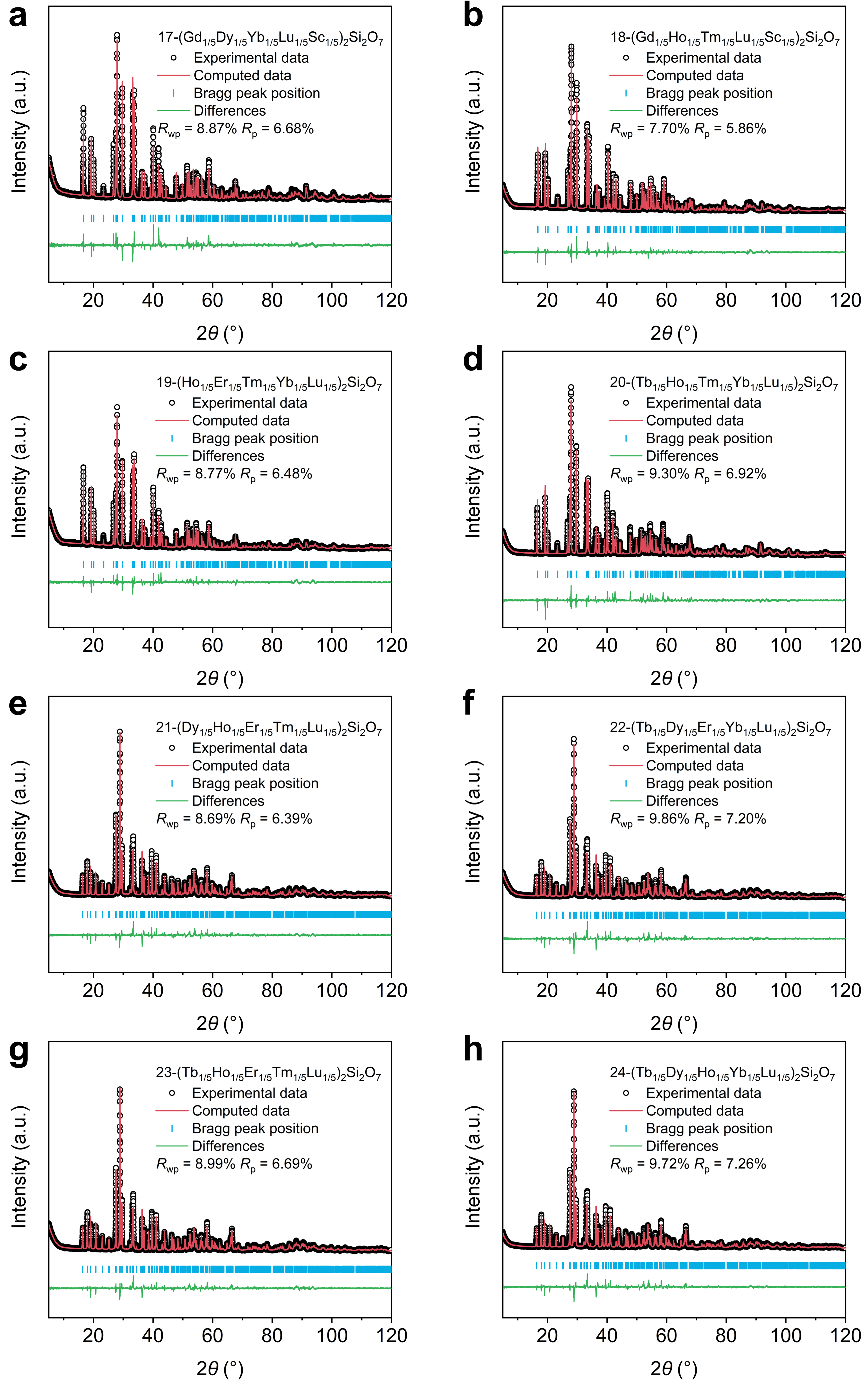

Supplement: Supplementary 1 — Figs. S1 to S28 Tables S1 to S8 [file research.1308.f1.zip › Fig_S4.tif]

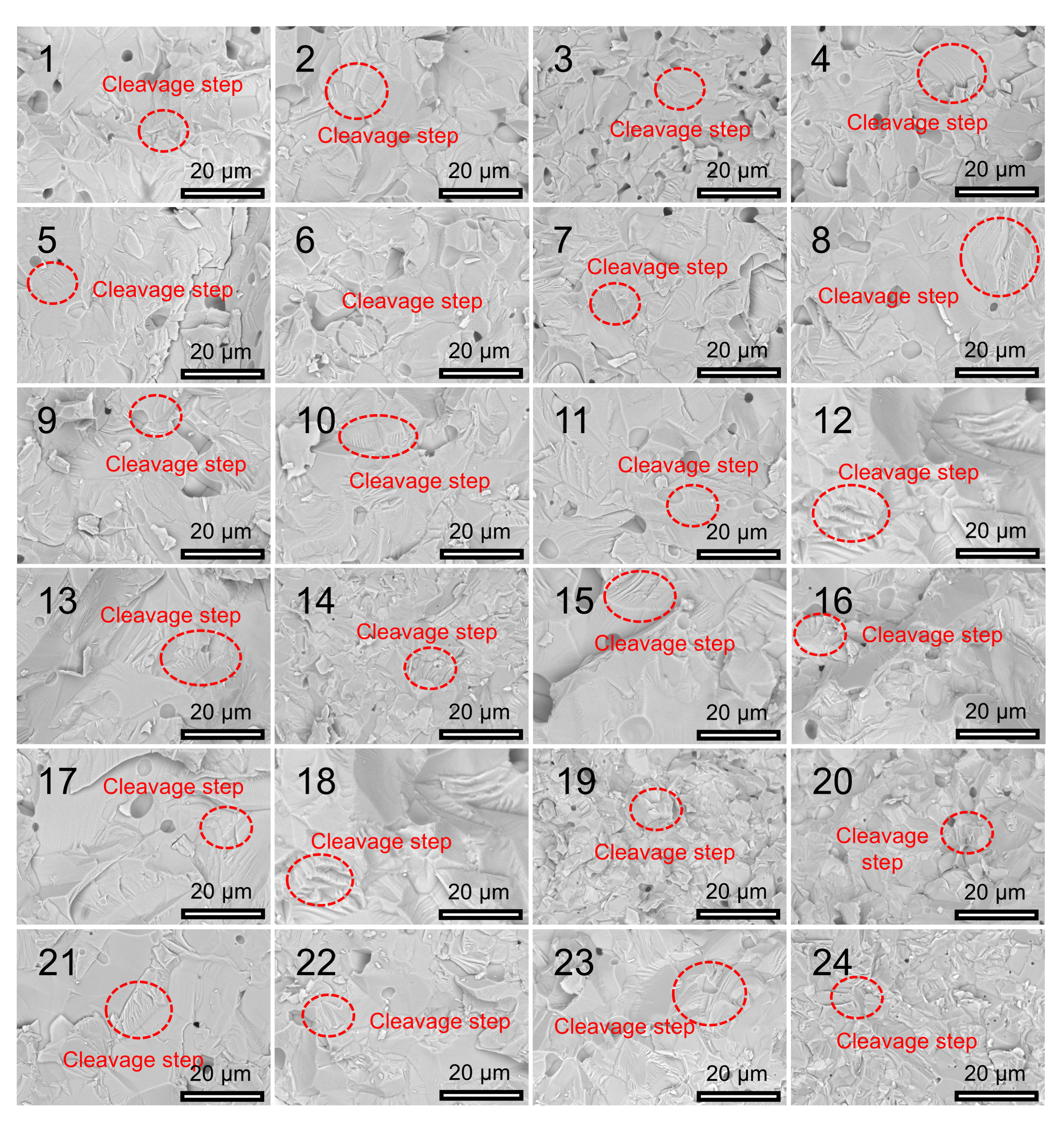

Supplement: Supplementary 1 — Figs. S1 to S28 Tables S1 to S8 [file research.1308.f1.zip › Fig_S5.tif]

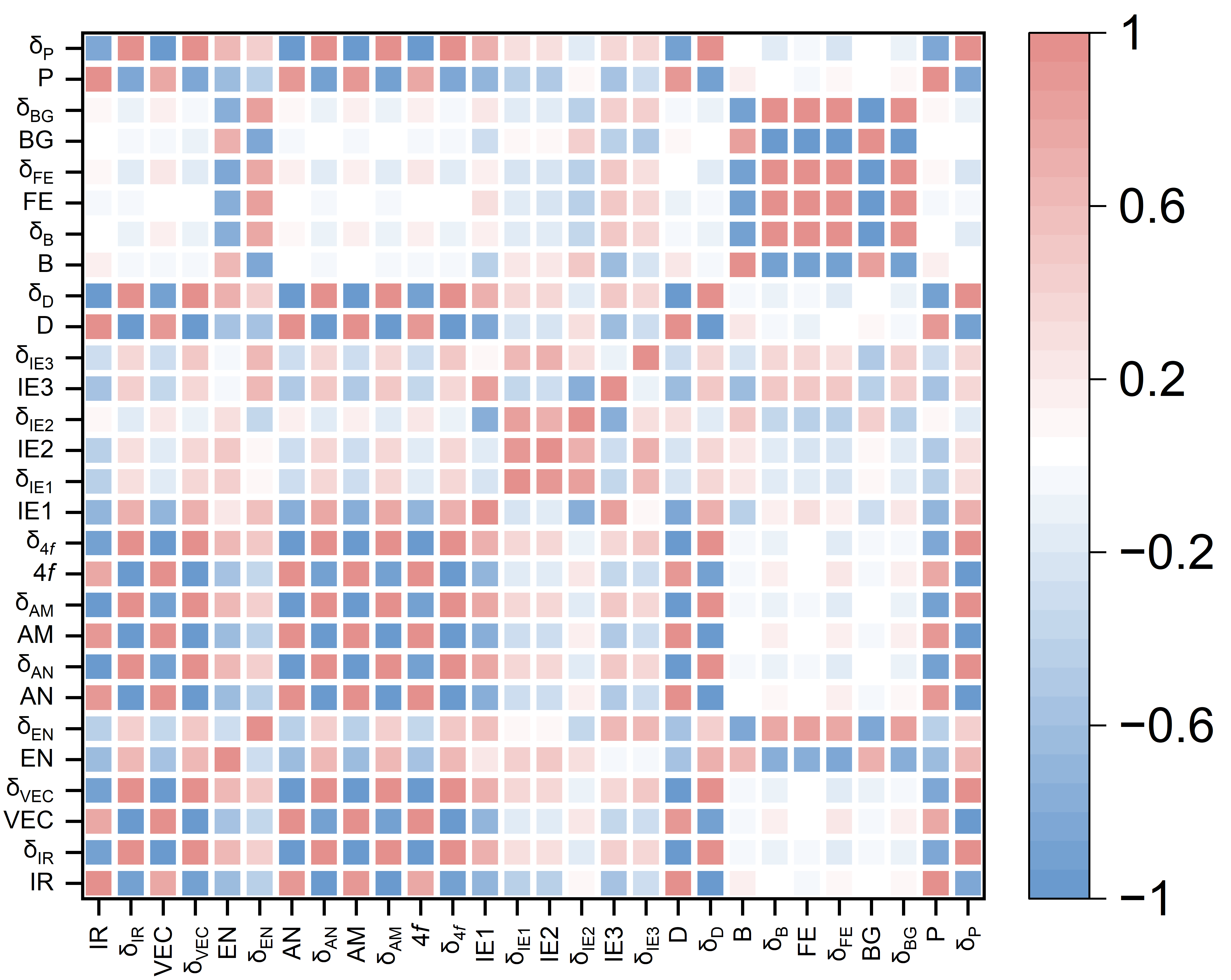

Supplement: Supplementary 1 — Figs. S1 to S28 Tables S1 to S8 [file research.1308.f1.zip › Fig_S9.tif]
